# Catalytic thiolation-depolymerization-like decomposition of oxyphenylene-type super engineering plastics *via* selective carbon–oxygen main chain cleavages

**DOI:** 10.1038/s42004-024-01120-7

**Published:** 2024-02-20

**Authors:** Yasunori Minami, Sae Imamura, Nao Matsuyama, Yumiko Nakajima, Masaru Yoshida

**Affiliations:** 1 Interdisciplinary Research Center for Catalytic Chemistry (IRC3), National Institute of Advanced Industrial Science and Technology (AIST), Tsukuba Central 5, 1-1-1 Higashi, Tsukuba, Ibaraki 305-8565 Japan; 2 PRESTO, Japan Science and Technology Agency (JST), 1-1-1 Higashi, Tsukuba, Ibaraki 305-8565 Japan

**Keywords:** Sustainability, Synthetic chemistry methodology, Polymer chemistry

## Abstract

As the effective use of carbon resources has become a pressing societal issue, the importance of chemical recycling of plastics has increased. The catalytic chemical decomposition for plastics is a promising approach for creating valuable products under efficient and mild conditions. Although several commodity and engineering plastics have been applied, the decompositions of stable resins composed of strong main chains such as polyamides, thermoset resins, and super engineering plastics are underdeveloped. Especially, super engineering plastics that have high heat resistance, chemical resistance, and low solubility are nearly unexplored. In addition, many super engineering plastics are composed of robust aromatic ethers, which are difficult to cleave. Herein, we report the catalytic depolymerization-like chemical decomposition of oxyphenylene-based super engineering plastics such as polyetheretherketone and polysulfone using thiols via selective carbon–oxygen main chain cleavage to form electron-deficient arenes with sulfur functional groups and bisphenols. The catalyst combination of a bulky phosphazene base P_4_-*t*Bu with inorganic bases such as tripotassium phosphate enabled smooth decomposition. This method could be utilized with carbon- or glass fiber-enforced polyetheretherketone materials and a consumer resin. The sulfur functional groups in one product could be transformed to amino and sulfonium groups and fluorine by using suitable catalysts.

## Abstract (web summary)

Although depolymerization methods for various commodity plastics and several engineering plastics have been developed, such methods for robust super engineering plastics that have very high heat and chemical resistance are nearly unexplored. Here, the authors report the catalytic depolymerization-like chemical decomposition of oxyphenylene-based super engineering plastics such as polyetheretherketone, polysulfone, and polyetherimide using thiols via selective carbon–oxygen main chain cleavage to form monomer-type molecules, namely electron-deficient arenes with sulfur functional groups and bisphenols.

## Introduction

Organic materials and products, from commodity plastics to engineering plastics and stable super engineering plastics are indispensable for society and are utilized in a variety of fields from general-purpose products to advanced materials. However, since the organic resources that comprise them are naturally finite, future societies will be required to reuse and recycle them once consumed, rather than simply dispose of them. One of the methodologies to achieve this goal is chemical recycling, i.e., the conversion of organic products into raw materials by means of organic reactions^1–14^. In this scenario, gasification chemical recycling of waste plastics to produce methanol, propylene, olefins, and so on is a promising method. However, gasification requires high-temperature conditions, and the resulting products must be converted back into organic raw substrates. Thus, chemical decomposition methodologies that convert plastics directly into raw organic compounds such as monomers at lower temperatures are becoming increasingly important. Especially, plastics and polymers having relatively cleavable main chains such as an ester group are useful for this purpose and are being developed. For example, chemical recycling of polyethylene terephthalate (PET) has been extensively studied, giving usable low-weight molecules^15–20^.

**Fig. 1:**
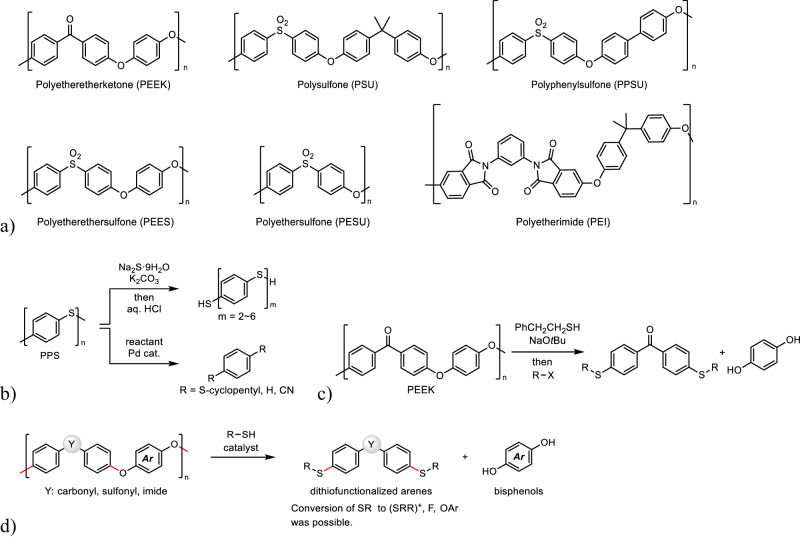
Chemical decomposition of super engineering plastics. **a** Examples of super engineering plastics. **b** Decomposition of PPS. **c** Previous work: PEEK decomposition using sulfur nucleophiles. **d** This work: Catalytic depolymerization-like chemical decomposition using thiols to afford dithiofunctionalized arenes and bisphenols. R–SH, organic thiol. R–X, organic halide. Ar, aryl.

As mentioned above, many studies have developed the methodologies of the chemical decomposition of various resins, and recently, the focus is on catalytic decomposition for highly stable resins composed of strong main chains such as polyamides, polyurethanes, polyureas, thermoset resins, and super engineering plastics. For example, Nylon-6 was found to undergo decomposition in the presence of a dimethylaminopyridine^21–24^ or lanthanide^25^ catalyst to form ε-caprolactam. Catalytic hydrogenolysis was applicable to the decomposition of polyamides to produce amino alcohols^26,27^. Polyurethanes^28,29^ and polyureas^30,31^ were also subjected to catalytic hydrogenation to afford anilines, polyols, and amines. Decomposition of epoxy resins was developed using catalytic main-chain cleavage to provide the corresponding monomers^32–34^. Thus, the catalytic approach has the potential to achieve the decomposition of such stable resins to form useful low-weight molecules such as monomers. Among these stable resins, super engineering plastics are known for their excellent stability such as heat resistance and chemical resistance. Based on their high stability, these resins are indispensable to industries such as the automotive medical, aerospace, and other industries. However, catalytic decomposition of super engineering plastics remains nearly unexplored. A few catalytic decompositions of polyphenylenesulfide (PPS) composed of phenyl–sulfur bonds were reported to give low-molecular-weight molecules such as 1,4-dicyclopentylthiobenzene, benzene, and 1,4-dicyanobenzene (Fig. 1b)^35–38^. This scarcity of reports emphasizes the difficulty of catalytic decomposition of super engineering plastics. In addition, many super engineering plastics are composed of stable aromatic ethers, which are not easily cleaved.

Recently, we demonstrated that thiolate reagents are highly effective for the depolymerization-like chemical decomposition of PEEK using sulfur nucleophiles, giving monomer-like products, dithiofunctionalized benzophenones and hydroquinone (Fig. 1c)^39^. The electron-deficient carbonyl group in the PEEK main chain enhances the reactivity of the carbon-oxygen bond at the *para* position such that the highly nucleophilic thiolate reagents cleave this bond selectively. We applied this system to the chemical decomposition of PSU, PESU, and PEEK using stoichiometric amounts of CsOH·H_2_O and CaH_2_ to form the corresponding bisphenols^40^. We expected that these stoichiometric methods have the potential to be applied to base-catalyzed chemical decomposition of various super engineering plastics. Since the previous reactions proceeded smoothly under a moderate reaction temperature (150 °C), the proposed catalytic strategy is expected to enable equally mild transformation to provide monomer-like products in high yields. Herein, we report the catalytic depolymerization-like chemical decomposition of oxyphenylene-based super engineering plastics using thiols to form monomer-like products, dithiofunctionalized arenes, and bisphenols (Fig. 1d). This method was applicable to PEEK, PSU, PPSU, and PEI. Inorganic bases and phosphazene bases were effective catalysts for this decomposition. Since the sulfur functional group acts as a leaving group for the substitution reaction under appropriate conditions^41,42^, the produced dithiofunctionalized arenes could be converted into sulfonium cations followed by fluorination or aryloxylation reactions.

## Results and Discussion

### Optimization of the reaction conditions

**Fig. 2:**
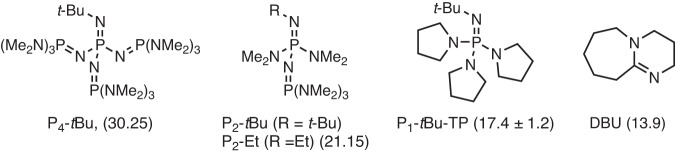
Structure of used organic bases. These p*K*_a_ values in DMSO are shown in parentheses. Bases such as P_2_-*t*Bu and P_4_-*t*Bu with high basicity showed catalytic activity.

We examined the chemical decomposition of insoluble polyetheretherketone (PEEK) powder (*M*_w_ ~ 20800 and *M*_n_ ~ 10300 as catalog specifications) (**1**) with 2-ethyl-1-hexanethiol (**2a**) (2 equiv. relative to monomer unit) in 1,3-dimethyl-2-imidazolidinone (DMI) under various conditions (Table 1). The decomposition was first performed using KOH, K_3_PO_4_, KO*t*Bu, and Cs_2_CO_3_ as catalysts (10 mol% relative to monomer unit) at 150 °C to form the corresponding decomposed products, dithiobenzophenone **4a**, 1,4-hydroquinone (**5**) and a benzophenone-hydroquinone-type dimer intermediate **3** (Table 1, Entries 1-4). The use of Cs_2_CO_3_ was especially effective to form the final decomposition monomers, **4a** and **5**, in good yields (Table 1, Entry 4), indicating that large counter cation sizes as well as basicity promote the decomposition. Encouraged by these results, we expected that bulky and strongly basic organic phosphazene bases such as P_4_-*t*Bu (p*K*_BH+_ 30.25 in dimethylsulfoxide (DMSO))^43–48^ would be promising catalysts for this decomposition (Fig. 2), which enhances the nucleophilicity of the counteranions^49–63^. For example, Shigeno, Korenaga, and Kondo recently reported that P_4_-*t*Bu activates an alkanethiol (p*K*_a_ of *n*-BuSH: 17.0 in DMSO)^64^. In this study, highly basic phosphazene bases P_4_-*t*Bu and P_2_-*t*Bu (p*K*_BH+_ of P_2_-Et: 21.15 in DMSO) exhibited good catalytic activity in comparison with weaker bases such as DBU (p*K*_BH+_ 13.9 in DMSO) and P_1_-*t*Bu-TP (p*K*_BH+_ 17.4 ± 1.2 in DMSO) (Table 1, Entries 5-8). Thus, the basicity and size of the catalysts are important for this reaction to enhance the nucleophilicity of the counter anion. Increasing the amount of **2a** from 2 equiv. to 2.5 equiv. enhanced the yield of **4a** and **5** (Table 1, Entry 9). On the other hand, high loading of P_4_-*t*Bu (20 mol%) had little effect (see Supplementary Information, Table S1, Entry 11), suggesting that increasing the amount of P_4_-*t*Bu does not directly lead to an increase in yields of **4a** and **5**. The P_4_-*t*Bu catalyst loading was successfully reduced to 5 mol%, albeit with slightly decreased yield (Table 1, Entry 10). The reaction at lower temperatures (120 and 100 °C) decreased the yield (Table 1, Entries 11 and 12). As mentioned above, PEEK is insoluble in organic solvents, but previous studies^39,40^ showed that solvents affect the reactivity of the decomposition. So, we checked the solvent effects for this decomposition in detail. As a result, *N*,*N*-dimethylacetamide (DMAc) was effective in the conditions whereas other solvent such as *N*,*N*-dimethylformamide (DMF), benzonitrile (PhCN), diethylene glycol diethyl ether ((C_2_H_5_OCH_2_CH_2_)_2_O), and xylene decreased the yield (Table 1, Entries 13-17). P_4_-*t*Bu dissolves in these solvents so that the decomposition reactivity may be affected by the polarity of the solvents^53^. Finally, we found that the catalyst combination of P_4_-*t*Bu (10 mol%) and K_3_PO_4_ (5 mol%) enhanced the reactivity of the present decomposition and gave **4a** and **5** in excellent yields in DMAc solvent (Table 1, Entry 18, see Method and section 7-1 in Supplementary Methods).

**Table 1:** Optimization of catalytic chemical decomposition to form monomer-like products^a^


Entry	2a (equiv.)	Catalyst	Solvent	Temp. (ºC)	3 (%)	4a (%)	5 (%)
1	2	KOH	DMI	150	17	41	41
2	2	K_3_PO_4_	DMI	150	12	54	56
3	2	KO*t*Bu	DMI	150	12	62	62
4	2	Cs_2_CO_3_	DMI	150	13	67	67
5	2	DBU	DMI	150	10	3	3
6	2	P_1_-*t*Bu-TP	DMI	150	7	2	1
7	2	P_2_-*t*Bu	DMI	150	14	61	66
8	2	P_4_-*t*Bu	DMI	150	16	67	72
9	2.5	P_4_-*t*Bu	DMI	150	7	74	73
10	2.5	P_4_-*t*Bu (5 mol%)	DMI	150	12	58	58
11	2.5	P_4_-*t*Bu	DMI	120	11	50	45
12	2.5	P_4_-*t*Bu	DMI	100	12	37	31
13	2.5	P_4_-*t*Bu	DMAc	150	-	95	84
14	2.5	P_4_-*t*Bu	DMF	150	3	65	52
15	2.5	P_4_-*t*Bu	PhCN	150	28	32	12
16	2.5	P_4_-*t*Bu	(EtOCH_2_CH_2_)_2_O	150	19	10	3
17	2.5	P_4_-*t*Bu	Xylene	150	19	11	2
18	2.5	P_4_-*t*Bu + K_3_PO_4_ (5 mol%)	DMAc	150	-	>99 (85)	>99 (61)

^a^ A mixture of **1** (powder, 0.1 mmol relative to the molecular weight of the monomer), **2a** (0.2 mmol for entries 1-8, 0.25 mmol for entries 9-18), catalyst (0.01 mmol), and solvent (0.2 mL) was stirred for 16 h. Yields were determined by ^1^H NMR. Numbers in parentheses are isolated yields.

### Experimental mechanistic studies

**Fig. 3:**
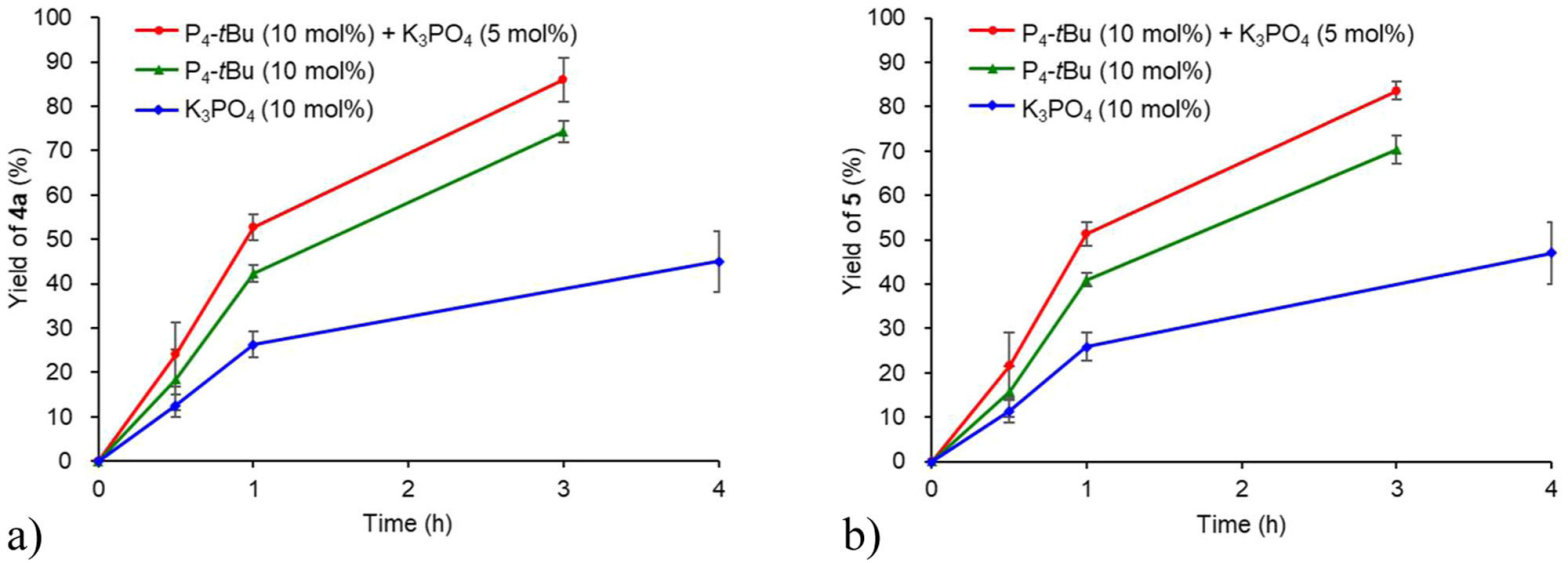
PEEK decomposition time course. Reaction conditions: PEEK (0.1 mmol relative to the molecular weight of monomer), **2a** (2.5 equiv.) at 150 °C in the presence of P_4_-*t*Bu (10 mol%) and K_3_PO_4_ (5 mol%) (red line), P_4_-*t*Bu (10 mol%) (green line), and K_3_PO_4_ (10 mol%) (blue line). **a** Yields of **4a** under various conditions are plotted as the average of the three runs with standard errors. **b** Yields of **5** under various conditions are plotted as the average of the three runs with standard errors.

To evaluate the present catalytic decomposition reactivity, we monitored the yields of **4a** and **5** during the reaction of PEEK powder **1** with **2a** catalyzed by P_4_-*t*Bu (10 mol%) and K_3_PO_4_ (5 mol%), P_4_-*t*Bu (10 mol%), and K_3_PO_4_ (10 mol%) (Fig. 3, see section 9-1 and Table S5 in Supplementary Methods). Under the three conditions, **4a** and **5** were formed after 30 minutes. Moreover, high yields of **4a** and **5** were obtained after 3 h under the conditions using P_4_-*t*Bu and K_3_PO_4_. These observations indicate that the decomposition proceeded rapidly. When P_4_-*t*Bu catalyst was only used, decomposition proceeded faster than when K_3_PO_4_ catalyst was used. The catalyst combination of P_4_-*t*Bu and K_3_PO_4_ increased the rate of formation of **4a** and **5** compared to the use of P_4_-*t*Bu alone. These results indicate that the use of the P_4_-*t*Bu catalyst allowed for rapid decomposition. The K_3_PO_4_ assisted this catalytic activity of P_4_-*t*Bu.

**Fig. 4:**
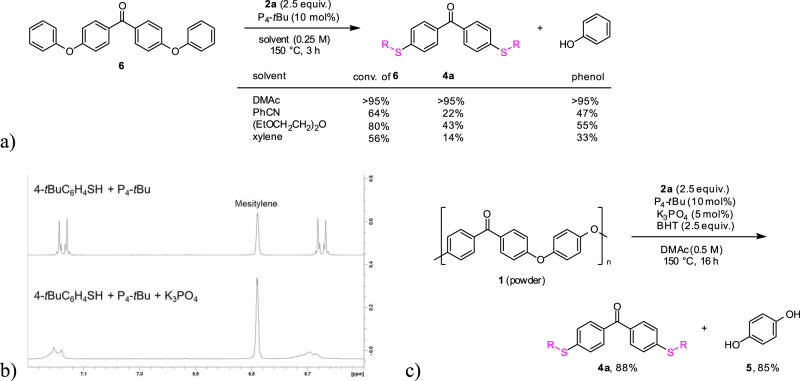
Mechanistic studies. **a** Examination of the solvent effect using a model substrate **6** under P_4_-*t*Bu catalyst. Yields of the products were determined by ^1^H NMR. **b** ^1^H NMR spectra indicating formation of reactive thiolate by reaction of 4-*tert*-butylphenylthiol (0.02 mmol) and P_4_-*t*Bu (0.02 mmol) in the presence or absence of K_3_PO_4_ (0.02 mmol) in DMF-*d*_7_. Mesitylene was used as an internal standard. **c** Examination of decomposition of PEEK in the presence of 3,5-di-*tert*-butyl-4-hydroxytoluene (BHT). The radical inhibitor does not affect the decomposition.

To understand the solvent effect for the decomposition of PEEK, we examined the swelling behavior of PEEK resins. PEEK granules or plates were heated in solvents such as DMAc, DMF, PhCN, (EtOCH_2_CH_2_)_2_O, and xylene at 150 °C for 19 h (See section 9-2, Table S6, and Fig. S1 in Supplementary Methods). As a result, these solvents increased the mass of the PEEK granules and plates (105~109 wt%) whereas the resins were apparently unchanged. These observations suggested that the swelling effect of PEEK does not affect the decomposition reactivity. Then we examined the reaction of 4,4’-diphenoxy-benzophenone (**6**) as a PEEK model compound with 2.5 equiv. of **2a** and 10 mol% of P_4_-*t*Bu at 150 °C for 3 h (Fig. 4a). The reaction using DMAc formed **4a** and phenol in an excellent yield. On the other hand, use of other solvents such as PhCN, (C_2_H_5_OCH_2_CH_2_)_2_O, and xylene decreased the yield of **4a**. In these cases, the reactions were not complete even after 22 h. Thus, DMAc as the high polar solvent enhanced the reactivity of the thiolate generated by the combination of the thiol and P_4_-*t*Bu and probably promoted the cleavage of the carbon–oxygen bonds and the decomposition^45^.

Next, we carried out NMR experiments to shed light on the combination of the thiol, P_4_-*t*Bu, and K_3_PO_4_. The reaction of 4-*tert*-butylphenylthiol (0.02 mmol) and P_4_-*t*Bu (0.02 mmol) in the presence of K_3_PO_4_ (0.02 mmol) was examined in DMF*-d*_7_ (0.5 mL) at 25 °C (see section 9-3 in Supplementary Methods). As a result, a ^31^P{^1^H} NMR spectrum suggested the formation of [P_4_-*t*Bu-H]^+^ (Fig. 4b, see Supplementary Fig. S5 compared with Fig. S2) and mass peaks were also observed at *m/z* 634 in ESI-TOF-(+)-MS and *m/z* 165 in ESI-TOF-(-)-MS mass spectra, confirming the generation of [P_4_-*t*Bu-H]^+^·[S(C_6_H_4_-*t*Bu)]^-^. The same results were observed in the absence of K_3_PO_4_ (see Supplementary Fig. S3). On the other hand, in ^1^H NMR spectrum, the resonances for the aryl doublets (δ 6.69 and δ 7.14) were broadened in comparison with the case in the absence of K_3_PO_4_ (Fig. 4b). In addition, these signals were different from the combination of the thiol and K_3_PO_4_ (see Supplementary Fig. S4). These results indicated that [P_4_-*t*Bu-H]^+^·[S(C_6_H_4_-*t*Bu)]^-^ was initially formed and the [S(C_6_H_4_-*t*Bu)] anion coordinated to K_3_PO_4_ in the equilibrium state. Density functional theory (DFT) calculations suggested that the NBO charge of the phenylthiolate coordinating to K_3_PO_4_ is more nucleophilic than the non-coordinating one (see section 9-4 in Supplementary Methods and Supplementary Data 2). We assumed that this catalyst combination activates the thiol for the smooth decomposition of PEEK.

Aromatic nucleophilic substitution with thiolate anions is known to proceed via the S_N_Ar or S_RA_1 mechanism^65–67^. In the S_RA_1 mechanism, thiyl radicals are thought to be involved. However, this catalytic decomposition of PEEK gives hydroquinone which inhibits the generation of free radicals. We examined the decomposition with **2a** under P_4_-*t*Bu/K_3_PO_4_ catalyst with 3,5-di-*tert*-butyl-4-hydroxytoluene (BHT, 2.5 equiv.), a radical inhibitor, at 150 °C for 16 h and observed the formation of **4a** and **5** in high yields (Fig. 4c). These results ruled out the possibility of a radical pathway for the decomposition. Of note, 2,2,6,6-tetramethylpiperidine 1-oxyl (TEMPO) as a typical radical scavenger was not suitable for this experiment, which converted **2a** into the corresponding disulfide in the absence of PEEK (see section 9-5 and Table S7, S8 in Supplementary Methods)^68^.

### Proposed mechanism

**Fig. 5:**
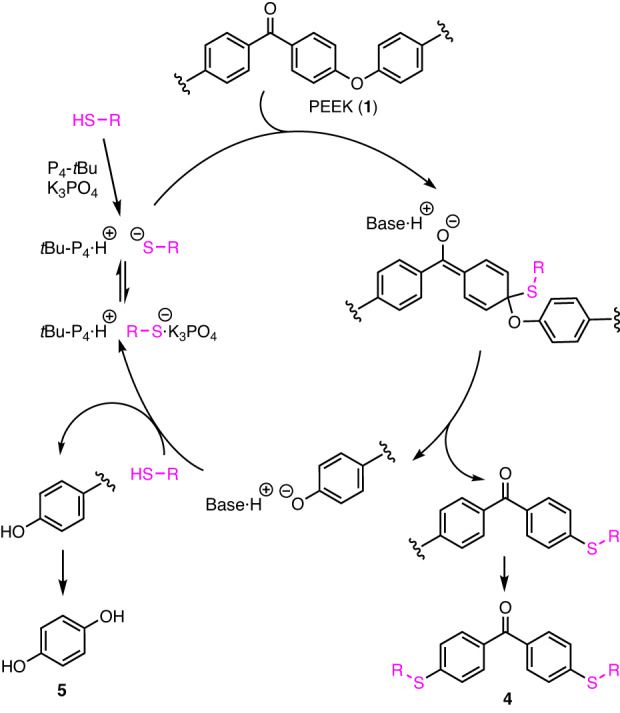
Plausible pathway for chemical decomposition of PEEK. Cleavage of carbon-oxygen main chains by organo thiolate generated from the reaction of thiol and catalysts.

A plausible pathway for the chemical decomposition catalyzed by P_4_-*t*Bu and K_3_PO_4_ is shown in Fig. 5. The thiol is initially activated by P_4_-*t*Bu to form a thiolate that interacts with K_3_PO_4_ in the equilibrium state. The sulfur center of the thiolate attacks the *ipso*-carbon bound to oxygen in the benzophenone unit in PEEK to form an anionic intermediate. The aryloxy anion is released to complete carbon–sulfur bond formation. K_3_PO_4_ may enhance the reactivity of the thiolate and assist in the release of the aryloxy anion. The generated aryloxy anion activates the thiol to form the organic thiolate and arenols. In fact, the basicity of arenols (p*K*_a_ in DMSO of PhOH: 18.0; *p*-MeC_6_H_4_OH: 18.9)^69^ is higher than that of thiols (p*K*_a_ in DMSO of *n*-BuSH: 17.0; PhSH: 10.3)^64^. This series of processes occurs repeatedly to generate the dithiobenzophenone **4** and hydroquinone (**5**).

### Substrate scope

With the optimum conditions using both P_4_-*t*Bu and K_3_PO_4_ in hand, we examined the chemical decomposition of other super engineering plastics such as polysulfone (PSU), polyetherethersulfone (PEES), polyphenylsulfone (PPSU), polyethersulfone (PESU), and polyetherimide (PEI) which were analyzed by high-temperature GPC analysis prior to use (see section 9-6 and Table S9 in Supplementary Methods). These resins have cleavable aryl-oxygen bonds affected by electron-withdrawing groups in a manner similar to PEEK. PSU is composed of diphenylsulfone and bisphenol A. Since thiolate anions can cleave aryl-SO_2_ bonds^70–74^, we were concerned that the present catalytic method may cleave the aryl-SO_2_ bond in the diphenylsulfone unit as well as the target C–O main chain. However, we found that polysulfone (PSU) pellets **7** (purchased from Sigma-Aldrich) and **7’** (purchased from Acros Organics) with different *M*_w_ (*M*_w_ 35000 and *M*_w_ 60000) in each of the catalog specifications underwent the decomposition with **2a** via selective C–O bond cleavage^38^ to furnish the corresponding 4,4’-dialkylthiobenzosulfone (**8a**) and bisphenol A (**9**) in high yields (Table 2, Entries 1 and 2). In addition, there was no clear difference in the reaction rate between **7** and **7’** (see Supplementary Table S2). In the same way, PEES pellets (**10**) or PPSU powder (**11**) could be converted into **8a** and hydroquinone (**5**) or 4,4’-dihydroxybiphenyl (**12**) in high yields (Table 2, Entries 3 and 4). In the case of PESU (**13**) consisting of repeating oxy-diphenylsulfone units, three products **8a**, 4-alkylthio-4’-hydroxy-diphenylsulfone **14**, and bisphenol S (**15**) were obtained (Table 2, Entry 5). PEI is composed of repeating structures of phenylene-1,3-bisphthalimide and bisphenol A. In this case, imide bonds in the phthalimide units may be cleaved by sulfur nucleophiles^75^. Nevertheless, the C–O main chains were successfully cleaved selectively in the decomposition of PEI pellets **16** with **2a**, giving dithiofunctionalized phenylene-1,3-bisphthalimide **17** and **9** in good yields (Table 2, Entry 6).

**Table 2:**
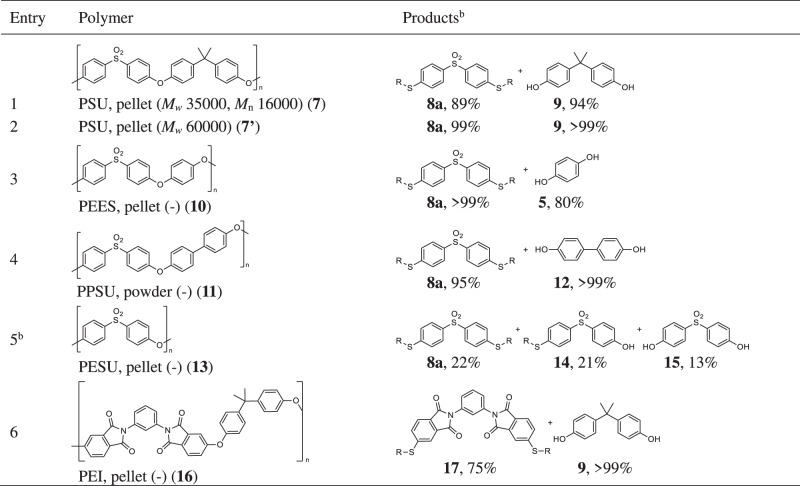
Scope of super engineering plastics for the chemical decomposition with 2-ethylhexanethiol in the presence of P_4_-*t*Bu and K_3_PO_4_ in DMAc^a^

^a^ A mixture of polymer (0.1 mmol relative to the molecular weight of the monomer), **2a** (0.25 mmol), P_4_-*t*Bu (0.01 mmol), K_3_PO_4_ (0.005 mmol) and DMAc (0.2 mL) was stirred for 16 h at 150 ºC. Isolated yields are shown. ^b^ 4-((4-(4-((4-((2-Ethylhexyl)thio)phenyl)sulfonyl)phenoxy)phenyl)sulfonyl)phenol was obtained in 15% yield.

**Fig. 6:**
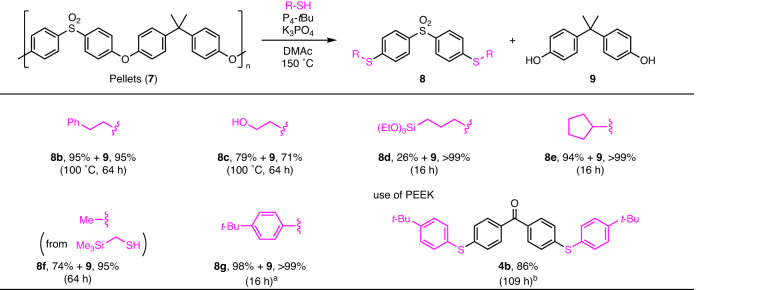
Scope of thiols for the chemical decomposition of PSU. Reaction conditions: a mixture of polymer (0.1 mmol relative to the molecular weight of monomer), thiol (0.25 mmol), P_4_-*t*Bu (0.01 mmol), K_3_PO_4_ (0.005 mmol), and DMAc (0.2 mL) was stirred for the time shown at 150 °C. Isolated yields are reported. ^a^ NaO*t*Bu (0.02 mmol) only was used as the catalyst. ^b^ Cs_2_CO_3_ (0.01 mmol) and DMI (0.2 mL) were used instead of K_3_PO_4_ and DMAc.

We then explored the scope of thiols under the catalytic decomposition of PSU pellets **7** (Fig. 6). 2-Phenylethanethiol or 2-mercaptoethanol underwent decomposition at 100 °C to form **8b** (see section 7-2 in Supplementary Methods) or **8c** and bisphenol A (**9**) in good yields. Triethoxysilyl-substituted propanethiol and cyclopentanethiol were used in the decomposition and the corresponding decomposition products **8d** and **8e** were obtained. Trimethylsilylmethylthiol gave 4,4’-dimethylthiodiphenylsulfone **8f** and **9** in high yields via desilylation. Not only alkanethiols but also 4-*tert*-butylbenzenethiol could be utilized for decomposition with only NaO*t*Bu catalyst (20 mol%) to form the corresponding monomer **8g** in 98% yield together with **9** quantitatively (see Supplementary Table S3 and section 7-3 in Supplementary Methods)). Instead of PSU, we attempted the decomposition of PEEK powder with 4-*tert*-butylbenzenethiol under the P_4_-*t*Bu/K_3_PO_4_ or NaO*t*Bu catalyst in DMAc but the yield of the product, 4,4’-di(arylthiol)benzophenone **4b**, was low (see Supplementary Table S4 and section 7-4 in Supplementary Methods). At that time, a suspension containing precipitated **4b** and its intermediates were obtained. Considering that the poor solubility of the products may have decreased the reactivity, we modified the conditions using a P_4_-*t*Bu/Cs_2_CO_3_ catalytic combination in DMI to enhance the solubility. As a result, **4b** was obtained in high yield, albeit with a long reaction time.

### Utility of the decomposition method

**Fig. 7:**
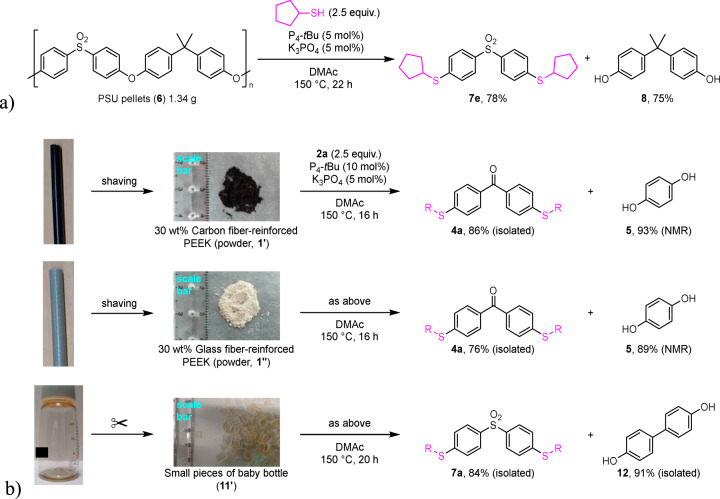
Utility of this decomposition method. **a** Gram-scale decomposition of PSU pellets (1.34 g) with cyclopentane thiol under P_4_-*t*Bu (5 mol%) and K_3_PO_4_ (5 mol%) catalysts in DMAc at 150 ºC. **b** Decomposition of 30% carbon fiber- or 30% glass fiber-reinforced PEEK or a baby bottle made up of PPSU with 2-ethylhexanethiol under the catalytic conditions using P_4_-*t*Bu (10 mol%) and K_3_PO_4_ (5 mol%) in DMAc at 150 ºC.

To demonstrate the scalability of the decomposition method, a gram-scale reaction of PSU pellets (**7**) with cyclopentanethiol catalyzed by 5 mol% of P_4_-*t*Bu and K_3_PO_4_ was carried out. The desired products **8e** and **9** were isolated in 78% and 75% yields, respectively (Fig. 7a, see section 7-5 in Supplementary Methods). It is worth noting that this catalytic method was applicable to composite materials. Shaved powder of 30 wt% carbon-fiber reinforced PEEK (**1’**) underwent the decomposition with **2a** to form **4a** and **5** in good yields comparable to those obtained from neat PEEK powder (Fig. 7b, see section 7-6 in Supplementary Methods). 30 wt% Glass-fiber reinforced PEEK (**1’’’**) was converted into **4a** and **5** in the same way. In addition, small pieces of a baby bottle made up of PPSU (**11’**) as a representative consumer resin were transformed into products, **8a** and **12**, in high yields (see section 7-7 in Supplementary Methods).

### Utility of products

**Fig. 8:**
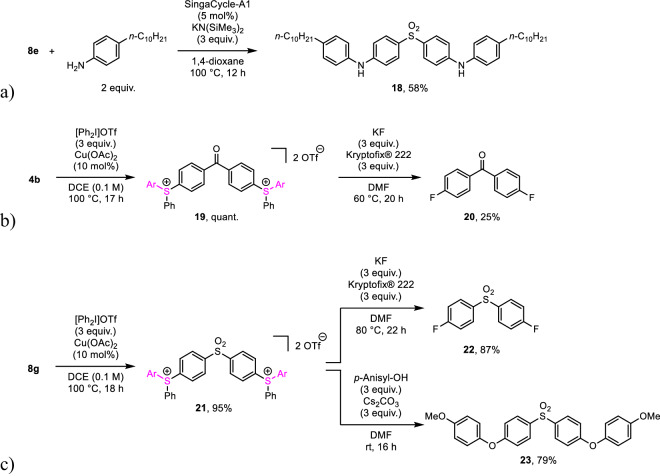
Functionalization of products. **a** Cross-coupling of **8e** with 4-decylaniline to give 4,4'-sulfonylbis(*N*-(4-decylphenyl)aniline) (**18**). **b** Conversion of **4b** into benzophenone-based disulfonium salt **19** followed by fluorination to form 4,4’-difluorobenzophenone (**20**). **c** Conversion of **8g** into diphenylsulfone-based disulfonium salt **21** followed by fluorination to form di(4-fluorophenyl)sulfone (**22**) or etherification to form 4,4’-bis(aryloxy)diphenylsulfone **23**. SingaCycle-A1: Chloro[[1,3-bis(2,6-diisopropylphenyl)imidazol-2-ylidene](*N*,*N*-dimethylbenzylamine)palladium(II)]. DCE: 1,2-dichloroethane. Kryptofix® 222: 4,7,13,16,21,24-hexaoxa-1,10-diazabicyclo[8.8.8]hexacosane. DMF: *N*,*N*-dimethylformamide. *p*-Anisyl: 4-methoxyphenyl.

Sulfur functional groups in the products can be utilized in various transformations to yield functional molecules. For example, **8e** was applicable to the double cross-coupling with 4-decylaniline under palladium-catalyzed conditions^76^ to give the corresponding double amination product **18** (Fig. 8a, see section 7-8 in Supplementary Methods). Double phenylation of **4b** using diphenyliodonium trifluoromethanesulfonate and copper acetate catalyst in 1,2-dichloroethane at 100 °C, based on a reported method^77^, gave benzophenone 4,4′-bis(diarylsulfonium) salt **19** in excellent yield (Fig. 8b, see section 7-9 in Supplementary Methods). Such sulfonium groups are more reactive leaving groups than their parent sulfur functional groups. Thus, the sulfonium groups in **19** could be converted into fluorine by potassium fluoride and Kryptofix® 222 (4,7,13,16,21,24-hexaoxa-1,10-diazabicyclo[8.8.8]hexacosane) in *N*,*N*-dimethylformamide at 60 °C (see section 7-10 in Supplementary Methods)^78^. Of note, the product, 4,4’-difluorobenzophenone (**20**), is used as a monomer for PEEK^79,80^. PSU-depolymerized product **8g** was also applicable to this transformation sequence. Double phenylation of **8g** afforded diphenylsulfone 4,4′-bis(diarylsulfonium) salt **21** (Fig. 8c, see section 7-11 in Supplementary Methods). Subsequent fluorination of **21** gave bis(4-fluorophenyl)sulfone (**22**) in 87% yield (see section 7-12 in Supplementary Methods), which is a monomer of diphenylsulfone-based polymers such as PSU^81,82^, PPSU^83–90^, PESU^91,92^, and PEES^93^. In addition, **21** reacted with *p*-methoxyphenol in the presence of Cs_2_CO_3_ to give 4,4’-bis(*p*-anisyloxy)diphenylsulfone (**23**) in 79% yield (see section 7-13 in Supplementary Methods).

## Conclusion

In this study, we demonstrated that the depolymerization-like chemical decomposition of robust super engineering plastics such as PEEK, PSU, PEES, PPSU, PESU, and PEI occurred smoothly with thiols at moderate temperature under the catalytic combination of bulky organic super bases, P_4_-*t*Bu, and inorganic bases, K_3_PO_4_ or Cs_2_CO_3_. DMAc solvent also promoted the carbon-oxygen bond cleavages in a low-weight molecule and insoluble PEEK due to its polarity under the conditions. Various thiols were applied to this decomposition to afford monomer-like thiofunctionalized arenes and bisphenols in high yields. In addition, carbon fiber- or glass fiber-reinforced resins and a baby bottle made of PPSU as a representative consumer resin material were utilized in this catalytic decomposition. From a synthetic perspective, thiofunctionalities in the arene products act as leaving groups and can be transformed into various substituents such as amino groups and fluorine. Notably, fluorinated arenes are parent monomers for synthesizing super engineering plastics. This shows that the present catalytic decomposition method can be utilized not only for chemical recycling but also for upcycling. This development will expand the decomposition of other robust polymer materials with various reagents under this effective catalytic system.

## Methods

### General procedure for catalytic chemical decomposition of PEEK

To a mixture of PEEK powder (28.8 mg, 0.100 mmol relative to the molecular weight of the monomer), and potassium phosphate tribasic (1.1 mg, 0.0050 mmol) was added *N*,*N*-dimethylacetamide (0.20 mL), P_4_-*t*Bu phosphazene base in hexane solution (1-*tert*-butyl-4,4,4-tris(dimethylamino)-2,2-bis[tris(dimethylamino)-phosphoranylidenamino]-2λ5,4λ5-catenadi(phosphazene), 0.8 M, 0.0125 mL, 0.010 mmol), and 2-ethylhexanethiol (36.6 mg, 0.250 mmol) in a 3 mL vial under argon atmosphere. The resultant mixture was stirred at 150 °C for 16 h. The reaction mixture was cooled to room temperature. The mixture was analyzed by ^1^H NMR in acetone-*d*_*6*_ to determine the yields of the products, **4a** and hydroquinone (**5**), using 1,4-dioxane as an internal standard. The reaction mixture was concentrated in vacuo. The crude product was purified by column chromatography on silica gel (hexane/ethyl acetate 96:4 to 7:3) to give bis(4-(2-ethylhexylthio)phenyl)methanone (85%, 39.9 mg) and 1,4-hydroquinone (61%, 6.7 mg).

**General information**. See Supplementary Methods, general information (page S3).

**Chemicals**. See Supplementary Methods, chemicals (page S3).

**NMR charts**. See Supplementary Data 1, NMR spectra of obtained chemicals.

### Supplementary information

Peer Review File

Supplementary Information

Description of Additional Supplementary File

Supplementary Data 1

Supplementary Data 2

## Data Availability

The data obtained in this study are available within this article and its supplementary information and are also from the corresponding authors upon reasonable request. Original ^1^H and ^13^C spectra of the compounds obtained in this manuscript are available in Supplementary Data 1. The computed energy values and coordinates are available in Supplementary Data. 2.

## References

[1] Ignatyev IA, Thielemans W, Beke BV (2014). Recycling of polymers: A review. ChemSusChem.

[2] Hong M, Chen EY-X (2017). Chemically recyclable polymers: a circular economy approach to sustainability. Green. Chem..

[3] Rahimi A, García JM (2017). Chemical recycling of waste plastics for new materials production. Nat. Rev. Chem..

[4] Lu Z-B, Liu Y, Zhou H (2018). Learning nature: recyclable monomers and polymers. Chem. Eur. J..

[5] Stadler BM, Wulf C, Werner T, Tin S, de Vries JG (2019). Catalytic approaches to monomers for polymers based on renewables. ACS Catal..

[6] Coates GW, Getzler YDYL (2020). Chemical recycling to monomer for an ideal, circular polymer economy. Nat. Rev. Mater..

[7] Kim JG (2020). Chemical recycling of poly(bisphenol A carbonate). Polym. Chem..

[8] Payne J, Jones MD (2021). The chemical recycling of polyesters for a circular plastics economy: challenges and emerging opportunities. ChemSusChem.

[9] Chen H, Wan K, Zhang Y, Wang Y (2021). Waste to wealth: chemical recycling and chemical upcycling of waste plastics for a great future. ChemSusChem.

[10] Liguori F, Moreno-Marrodán C, Barbaro P (2021). Valorisation of plastic waste via metal-catalysed depolymerization. Beilstein J. Org. Chem..

[11] Kosloski-Oh SC, Wood ZA, Manjarrez Y, de Los Rios JP, Fieser ME (2021). Catalytic methods for chemical recycling or upcycling of commercial polymers. Mater. Horiz..

[12] Fagnani DE (2021). 100th anniversary of macromolecular science viewpoint: redefining sustainable polymers. ACS Macro Lett..

[13] Xu G, Wang Q (2022). Chemically recyclable polymer materials: polymerization and depolymerization cycles. Green. Chem..

[14] Zhang Y, Qi M-Y, Tang Z-R, Xu Y-J (2023). Photoredox-catalyzed plastic waste conversion: nonselective degradation versus selective synthesis. ACS Catal..

[15] Fukushima K (2011). Organocatalytic Depolymerization of Poly(ethylene terephthalate). J. Poly. Sci. A: Poly. Chem..

[16] Tanaka S, Sato J, Nakajima Y (2021). Capturing ethylene glycol with dimethyl carbonate towards depolymerisation of polyethylene terephthalate at ambient temperature. Green. Chem..

[17] Lu H (2022). Machine learning-aided engineering of hydrolases for PET depolymerization. Nature.

[18] Joseph Ng KW (2023). A facile alternative strategy of upcycling mixed plastic waste into vitrimers. Commun. Chem..

[19] Perez-Garcia P (2023). An archaeal lid-containing feruloyl esterase degrades polyethylene terephthalate. Commun. Chem..

[20] Lourenço DL, Fernandes AC (2023). HBpin/MoO_2_Cl_2_(H_2_O)_2_ as an efficient catalytic system for the reduction of esters, lactones and polyester plastic waste. Mol. Catal..

[21] Kamimura A, Yamamoto S (2008). A novel depolymerization of nylons in ionic liquids. Polym. Adv. Technol..

[22] Yamamoto S, Kamimura A (2009). Chem. Lett..

[23] Kamimura K, Shiramatsu Y, Kawamoto T (2019). Green. Energy Environ..

[24] Alberti C, Figueira R, Hofmann M, Koschke S, Enthaler S (2019). Chemical recycling of end-of-Life Polyamide 6via ring closing depolymerization. ChemistrySelect.

[25] Wursthorn L (2023). Selective Lanthanide-organic catalyzed depolymerization of Nylon-6 to ɛ-Caprolactam. Angew. Chem. Int. Ed..

[26] Kumar A (2020). Hydrogenative depolymerization of nylons. J. Am. Chem. Soc..

[27] Zhou W (2021). Depolymerization of technical-grade polyamide 66 and polyurethane materials through hydrogenation. ChemSusChem.

[28] Liua X, Werner T (2021). Indirect reduction of CO_2_ and recycling of polymers by manganese-catalyzed transfer hydrogenation of amides, carbamates, urea derivatives, and polyurethanes. Chem. Sci..

[29] Gausas L (2021). Catalytic hydrogenation of polyurethanes to base chemicals: from model systems to commercial and end-of-life polyurethane materials. JACS Au.

[30] Kumar, A. & Luk, J. Catalytic Hydrogenation of Urea Derivatives and Polyureas. *Eur. J. Org. Chem*. 4546–4550 (2021).

[31] Iwasaki T, Tsuge K, Naito N, Nozaki K (2023). Chemoselectivity change in catalytic hydrogenolysis enabling urea-reduction to formamide/amine over more reactive carbonyl compounds. Nat. Commun..

[32] Nguyen ST, McLoughlin EA, Cox JH, Fors BP, Knowles RR (2021). Depolymerization of hydroxylated polymers via light-driven C−C bond cleavage. J. Am. Chem. Soc..

[33] Nguyen ST (2023). Chemical Recycling of thiol epoxy thermosets via light-driven C–C Bond cleavage. J. Am. Chem. Soc..

[34] Ahrens A (2023). Catalytic disconnection of C–O bonds in epoxy resins and composites. Nature.

[35] Wang SJ, Bian SG, Yan H, Xiao M, Meng YZ (2008). Novel synthesis of macrocyclic disulfides from poly(phenylene sulfide) by depolymerization reaction. J. Appl. Poly. Sci..

[36] Lian Z, Bhawal BN, Yu P, Morandi B (2017). Palladium-catalyzed carbon-sulfur or carbon-phosphorus bond metathesis by reversible arylation. Science.

[37] Minami Y (2021). Catalytic reductive cleavage of poly(phenylene sulfide) using a hydrosilane. Synthesis.

[38] Delcaillau T, Woenckhaus-Alvarez A, Morandi B (2021). Nickel-catalyzed cyanation of aryl thioethers. Org. Lett..

[39] Minami Y (2023). Depolymerization of robust polyetheretherketone to regenerate monomer units using sulfur reagents. Commun. Chem..

[40] Minami Y, Inagaki Y, Tsuyuki T, Sato K, Nakajima Y (2023). Hydroxylation-depolymerization of oxyphenylene-based super engineering plastics to regenerate arenols. JACS Au.

[41] Yorimitsu H (2021). Catalytic transformations of sulfonium salts via C-S bond activation. Chem. Rec..

[42] Tian Z-Y, Hu Y-T, Teng H-B, Zhang C-P (2018). Application of arylsulfonium salts as arylation reagents. Tetrahedron Lett..

[43] Schwesinger R, Schlemper H (1987). Peralkylated polyaminophosphazenes- extremely strong, neutral nitrogen bases. Angew. Chem. Int. Ed..

[44] Schwesinger, R., et al*.* Extremely strong, uncharged auxiliary bases; monomeric and polymer-supported Polyaminophosphazenes (P_2_–P_5_). *Liebigs Ann*. 1055–1081 (1996).

[45] Tshepelevitsh, S., et al. On the basicity of organic bases in different media. *Eur. J. Org. Chem*. 6735–6748 (2019).

[46] Seebach, D., Beck, A. K. & Studer, A. in *Modern Synthetic Methods 1995* (eds Ernst, B. & Leumann, C.) 48−54 (Wiley-VCH, 1995).

[47] Mamdani HT, Hartley RC (2000). Tetrahedron Lett..

[48] Shannon RD (1976). Revised effective ionic radii and systematic studies of interatomic distances in halides and chalcogenides. Acta Cryst..

[49] Pietzonka T, Seebach D (1991). Alkylations of (R,R)-2-t-Butyl-6-methyl-1,3-dioxan-4-ones which are not Possible with Lithium Amides may be Achieved with a Schwesinger P4 Base. Chem. Ber..

[50] Fruchart J-S, Gras-Masse H, Melnyk O (2001). Methyl phenylacetate enolate generated with the P_4_-*t*Bu Schwesinger base: ‘naked’ or not?. Tetrahedron Lett..

[51] Solladié-Cavallo A, Liptaj T, Schmitt M, Solgadi A (2002). *iso*-Propyl phenylacetate: formation of a single enolate with tBuP4 as base. Tetrahedron Lett..

[52] Schwesinger R, Link R, Wenzl P, Kossek S, Keller M (2006). Extremely base-resistant organic phosphazenium cations. Chem. -Eur. J..

[53] Ebisawa M, Ueno M, Oshima Y, Kondo Y (2007). Synthesis of dictyomedins using phosphazene base catalyzed diaryl ether formation. Tetrahedron Lett..

[54] Kolonko KJ, Reich HJ (2008). Stabilization of Ketone and Aldehyde Enols by formation of hydrogen bonds to Phosphazene Enolates and their aldol products. J. Am. Chem. Soc..

[55] Kolonko KJ, Guzei IA, Reich HJ (2010). Structure and dynamics of α-Aryl Amide and Ketone Enolates: THF, PMDTA, TMTAN, HMPA, and Crypt-Solvated Lithium Enolates, and comparison with Phosphazenium analogues. J. Org. Chem..

[56] Boileau S, Illy N (2011). Activation in anionic polymerization: Why phosphazene bases are very exciting promoters. Prog. Polym. Sci..

[57] Kawai H, Yuan Z, Tokunaga E, Shibata N (2013). A sterically demanding organo-superbase avoids decomposition of a naked trifluoromethyl carbanion directly generated from fluoroform. Org. Biomol. Chem..

[58] Jardel D, Davies C, Peruch F, Massip S, Bibal B (2016). Protonated Phosphazenes: Structures and hydrogen-bonding organocatalysts for carbonyl bond activation. Adv. Synth. Catal..

[59] Hong M, Chen EY-X (2016). Towards truly sustainable polymers: a metal-free recyclable polyester from biorenewable non-strained g-Butyrolactone. Angew. Chem., Int. Ed..

[60] Luo C, Bandar JS (2018). J. Am. Chem. Soc..

[61] Shigeno M, Hayashi K, Nozawa-Kumada K, Kondo Y (2019). Organic superbase *t*‑Bu-P_4_ catalyzes amination of methoxy(hetero)arenes. Org. Lett..

[62] Shigeno M, Hayashi K, Korenaga T, Nozawa-Kumada K, Kondo Y (2022). Organic superbase *t*-Bu-P_4_-catalyzed demethylations of methoxyarenes. Org. Chem. Front..

[63] Luo C (2022). J. Am. Chem. Soc..

[64] Bordwell FG, Hughes DL (1982). Thiol Acidities and Thiolate ion reactivities toward Butyl Chloride in Dimethyl Sulfoxide Solution. The question of curvature in Brønsted plots. J. Org. Chem..

[65] Rossi RA, Pierini AB, Peñéñory AB (2003). Nucleophilic substitution reactions by electron transfer. Chem. Rev..

[66] Zhang XM, Yang DL, Liu YC (1993). Effects of electron acceptors and radical scavengers on nonchain radical nucleophilic substitution reactions. J. Org. Chem..

[67] Zhang XM, Yang DL, Jia XQ, Liu YC (1993). Kinetic and mechanistic studies of the nonchain radical nucleophilic substitution reactions. J. Org. Chem..

[68] Orsi DL, Easley BJ, Lick AM, Altman RA (2017). Base catalysis enables access to α,α-Difluoroalkylthioethers. Org. Lett..

[69] Bordwell FG, McCallum RJ, Olmstead WN (1984). Acidities and hydrogen bonding of phenols in dimethyl sulfoxide. J. Org. Chem..

[70] Nove, M., Dell’Erba, C. & Sancassan, F. *ipso-* and tele-substitution pathways in the reactions of 1,3-Dimethyl-2,4-dinitro- and 1.3-Dimethyl-2-nitro-4-phenylsulphonylnaphthalene with Sodium Arenethiolates in Dimethyl Sulphoxide. *J. Chem. Soc. Perkin. Trans. I* 1145–1149 (1983).

[71] Starosotnikov AM, Shevelev SA (2003). Characteristic features of nucleophilic substitution in the series of 4-RSO_2_-6-nitro-1-phenyl-1*H*-indazoles and benzo[*d*]isoxazoles. Russ. Chem. Bull. Int. Ed..

[72] Thompson A (2005). Sulfur-based protecting groups for pyrroles and the facile deprotection of 2-(2,4-Dinitrobenzene)sulfinyl and Sulfonyl Pyrroles. J. Org. Chem..

[73] Zhou P, Yao J, Hu G, Fang J (2016). Naphthalimide scaffold provides versatile platform for selective thiol sensing and protein labeling. ACS Chem. Biol..

[74] Begunov RS, Valyaeva AN, Fakhrutdinov AN, Pirogova SA (2017). Synthesis of a new monomer for sulfonated poly(arylene ether sulfones). Russ. Chem. Bull. Int. Ed..

[75] Vamisetti GB, Meledin R, Nawatha M, Suga H, Brik A (2021). The development of a fluorescence-based competitive assay enabled the discovery of dimeric cyclic peptide modulators of ubiquitin chains. Angew. Chem. Int. Ed..

[76] Sugahara T, Murakami K, Yorimitsu H, Osuka A (2014). Palladium-catalyzed amination of aryl sulfides with anilines. Angew. Chem. Int. Ed..

[77] Takenaga, N., et al. Catalytic and non-catalytic selective aryl transfer from (mesityl)iodonium(III) salts to diarylsulfide compounds. *Arkivoc* 7–18 (2022).

[78] Mu, L., et al. ^18^F-Radiolabeling of aromatic compounds using Triarylsulfonium salts. *Eur. J. Org. Chem*. 889–892 (2012).

[79] Colquhoun HM, Hodge P, Paoloni FPV, McGrail PT, Cross P (2009). Reversible, nondegradative conversion of crystalline aromatic Poly(ether ketone)s into Organo-Soluble Poly(ether dithioketal)s. Macromolecules.

[80] Gunaratne HQN, Langrick CR, Puga AV, Seddon KR, Whiston K (2013). Production of polyetheretherketone in ionic liquid media. Green. Chem..

[81] Wang J, Liu Z (2012). An efficient synthetic strategy for high performance polysulfone: ionic liquid/zwitterion as reaction medium. Green. Chem..

[82] Park D-Y, Kohl PA, Beckham HW (2013). Anion-conductive multiblock aromatic copolymer membranes: structure−property relationships. J. Phys. Chem. C..

[83] García JM (2014). Meisenheimer complex inspired catalyst- and solvent-free synthesis of noncyclic Poly(aryl ether sulfone)s. Macromolecules.

[84] Yang Y, Muhich CL, Green MD (2020). Kinetics and mechanisms of polycondensation reactions between aryl halides and bisphenol A. Polym. Chem..

[85] Park S-A (2019). Sustainable and recyclable super engineering thermoplastic from biorenewable monomer. Nat. Commun..

[86] Hoshi T, Bae B, Watanabe M, Miyatake K (2012). Synthesis and properties of sulfonated Poly(arylene ether) block copolymers as proton conductive membranes. Bull. Chem. Soc. Jpn..

[87] Miyame J, Watanabe M, Miyatake K (2013). Sulfonated Poly(arylene ether phosphine oxide ketone) block copolymers as oxidatively stable proton conductive membranes. ACS Appl. Mater. Interfaces.

[88] Miyame J, Watanabe M, Miyatake K (2014). Intrapolymer Heck reaction for proton conductive ladder-type aromatic block copolymers. RSC Adv..

[89] Hoshi T, Miyake J, Watanabe M, Miyatake K (2015). Synthesis and properties of sulfonated and brominated Poly(arylene ether)s as proton conductive membranes. Bull. Chem. Soc. Jpn..

[90] Choi J (2018). Application of spirobiindane-based microporous poly(ether sulfone)s as polymeric binder on solid alkaline exchange membrane fuel cells. J. Membr. Sci..

[91] Faye A, Furtos A, Brisson J (2016). Synthesis of high molecular weight polyetherethersulfone-allyl copolymers of controlled glass transition. Macromol. Chem. Phys..

[92] Pirali-Hamedani M, Mehdipour-Ataei S (2017). Effect of sulfonation degree on molecular weight, thermal stability, and proton conductivity of poly(arylene ether sulfone)s membrane. Des. Monomers Polym..

[93] Wu Z (2017). Multi-sulfonated polyhedral oligosilsesquioxane (POSS) grafted poly(arylene ether sulfone)s for proton conductive membranes. Polymer.

