# Supplementary material for: Catalytic thiolation-depolymerization-like decomposition of oxyphenylene-type super engineering plastics via selective carbon–oxygen main chain cleavages
Source: Commun Chem. 2024 Feb 20;7:37. doi: 10.1038/s42004-024-01120-7 (PMC10879179; doi:10.1038/s42004-024-01120-7)
Supplement: Supplementary file 2 — Supplementary Information [file 42004_2024_1120_MOESM2_ESM.pdf]

Supplementary Information

Catalytic thiolation-depolymerization-like decomposition of oxyphenylene-type super engineering plastics *via* selective carbon–oxygen main chain cleavages

Yasunori Minami,[1,2] Sae Imamura,[1] Nao Matsuyama,[1] Yumiko Nakajima,[1] and Masaru Yoshida[1]

[1] Interdisciplinary Research Center for Catalytic Chemistry (IRC3), National Institute of Advanced Industrial Science and Technology (AIST), Tsukuba Central 5, 1-1-1 Higashi, Tsukuba, Ibaraki 305-8565, Japan

[2] PRESTO, Japan Science and Technology Agency (JST), 1-1-1 Higashi, Tsukuba, Ibaraki 305-8565, Japan

email: yasu-minami@aist.go.jp

Table of Contents

Supplementary Methods

1. General information..S3
2. Chemicals..S3
3. **Supplementary Table S1 |** Optimization of catalytic chemical decomposition.....................S4
4. **Supplementary Table S2 |** Decomposition of PSUs having different M_n and M_w....................S5
5. **Supplementary Table S3 |** Reaction of PSU pellets with 4-*tert*-butylbenzenethiol.................S5
6. **Supplementary Table S4 |** Reaction of PEEK powder with 4-*tert*-butylbenzenethiol..................S6
7. Experimental procedures...S7
8. Spectrum data of products..S13
9. Additional experiments and data
   - 9-1. Time course study...S19
   - 9-2. Checking weight change of PEEK sample by solvent at 150 °C.......................................S20
   - 9-3. NMR and ESI-TOF-MS analyses of the combination of arylthiol, P_4-*t*Bu, and K_3PO_4....S21
   - 9-4. NBO charges of phenylthiolate and phenylthiolate-K_3PO_4 complex by DFT calculation..S25
   - 9-5. Effect of TEMPO toward the catalytic decomposition.......................................S26
   - 9-6. Gel Permeation Chromatography (GPC) analysis of resins....................................S27

Supplementary references..S28

Supplementary Methods

1. General. All manipulations of oxygen- and moisture-sensitive materials were conducted in a dry box under an argon atmosphere. Flash column chromatography was performed using Biotage Sfär Silica D - Duo 60 µm. Analytical TLC was performed on Merck Kieselgel 60 F254 (0.25 mm) plates. Visualization was accomplished with UV light (254 nm). HPLC was performed by JAI LC-9210NEXT. ^{1}H and ^{13}C{^{1}H} NMR spectra in $CDCl_3$ or acetone-d_6 solution were recorded with Bruker AVANCE III HD 600 spectrometer. The ^{1}H NMR (600 MHz) and ^{13}C{^{1}H} NMR (151 MHz) chemical shifts were reported in δ (ppm). ^{1}H NMR and ^{13}C{^{1}H} NMR spectra were referenced to the residual solvent signals. ^{1}H NMR data are reported as follows: chemical shift, multiplicity (s = singlet, d = doublet, t = triplet, q = quartet, quint = quintet, sext = sextet, sept = septet, br = broad, m = multiplet), coupling constants (Hz), and integration. Melting points were measured by a MPA100 Optimelt Automated Melting Point System. High-resolution mass spectra (HRMS) were measured on a Bruker micrOTOF II mass spectrometer under positive electrospray ionization (ESI^{+}) or negative electrospray ionization (ESI^{-}) conditions.

2. Chemicals. All reactions were carried out under an argon atmosphere. Unless otherwise noted, commercially available reagents were used without further purification. Powder and pellet forms of polyetheretherketone (PEEK) (Powder: mean particle size 80micron, Cat. No. GF75065755. Granule: nominal granule size 2-3 mm, Cat. No. GF95232786.), polysulfone (PSU) (pellet (Transparent), average M_w ~35,000 by LS, average M_n ~16,000 by MO, Cat. No. GF75065755), poly(1,4-phenylene ether ether sulfone) (Pellet, Cat. No. 440965), Poly(oxy-1,4-phenylenesulfonyl-1,4-phenylene) (PESU) (Pellet (Transparent), Cat. No. 191094), and polyetherimide (PEI) (Pellet (Transparent), Cat. No. 700193) were purchased from Sigma–Aldrich Japan. PSU (pellet (Transparent), average M_w ~60,000, Cat. No. 178910050) was purchased from Nakarai. Carbon or glass fiber (30wt%)-reinforced PEEK material made from Ensinger (TECAPEEK CF30 or TECAPEEK GF30) was used after roughly ground, which were purchased from Monotaro (Cat. No. 3-3094-02 and 3-3095-01). PPSU plate was purchased from Standard-Testpiece (Cat. No. RMOLDED0050) and was grinded down to a powder form. PEEK plate was purchased from Az one (150 mm × 245 mm × 1 mm, Cat. No. 2-9239-01). Dehydrated 1,3-dimethyl-2-imidazolidinone (Cat. No. 11208-00) was purchased from the Kanto Chemicals. Dehydrated *N*,*N*-dimethylacetamide (Cat. No. 042-32353), and xylene (Cat. No. 240-00865) were purchased from the FUJIFILM Wako Chemicals.

3. **Supplementary Table S1 |** Optimization of catalytic chemical decomposition[a]

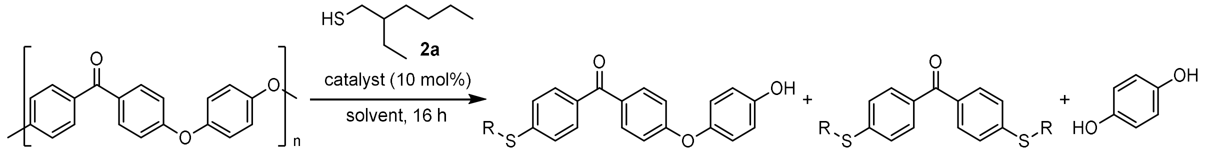

1 (powder) **3** (R = 2-Ethyl-Hexyl) **4a** (R = 2-Ethyl-Hexyl) **5**

Entry	**2a** (equiv.)	catalyst	solvent	Temp.	Time	**3** (%)	**4a** (%)	**5** (%)
1	2	KOH	DMI	150 °C	16 h	17	41	41
2	2	K_3PO_4	DMI	150 °C	16 h	12	54	56
3	2	KO*t*Bu	DMI	150 °C	16 h	12	62	62
4	2	NaO*t*Bu (20 mol%)	DMI	150 °C	20 h	(4)	(65)	49
5	2	Cs_2CO_3	DMI	150 °C	16 h	13	67	67
6	2	DBU	DMI	150 °C	16 h	10	3	3
7	2	P_1-*t*Bu-TP	DMI	150 °C	16 h	7	2	1
8	2	P_2-Et	DMI	150 °C	16 h	19	52	51
9	2	P_2-*t*Bu	DMI	150 °C	16 h	14	61	66
10	2	P_4-*t*Bu	DMI	150 °C	16 h	16	67	72
11	2	P_4-*t*Bu (20 mol%)	DMI	150 °C	16 h	13	65	59
12	2	P_4-*t*Oct	DMI	150 °C	16 h	26	33	30
13	2	P_4-*t*Bu (20 mol%)	DMAc	150 °C	21 h	11	70	75
14	2.5	P_4-*t*Bu	DMI	150 °C	16 h	7	74	73
15	2.5	P_4-*t*Bu (5 mol%)	DMI	150 °C	16 h	12	58	58
16	2.5	P_4-*t*Bu	DMI	120 °C	16 h<br>39 h	11<br>18	50<br>72	45<br>64
17	2.5	P_4-*t*Bu	DMI	100 °C	16 h<br>107 h	12<br>19	37<br>67	31<br>48
18	2.5	P_4-*t*Bu	DMAc	150 °C	16 h	<1	95	84
19	2.5	P_4-*t*Bu	NMP	150 °C	16 h	11	68	57
20	2.5	P_4-*t*Bu	DMF	150 °C	16 h	3	65	52
21	2.5	P_4-*t*Bu	PhCN	150 °C	16 h	28	32	12
22	2.5	P_4-*t*Bu	$(EtOCH_2CH_2)_2O$	150 °C	16 h	19	10	3
23	2.5	P_4-*t*Bu	1,4-dioxane	100 °C	16 h	24	6	1
24	2.5	P_4-*t*Bu	Xylene	150 °C	16 h	19	11	2
25	2.5	P_4-*t*Bu + K_3PO_4 (5 mol%)	DMI	150 °C	16 h	5	95	94
26	2.5	P_4-*t*Bu + K_3PO_4 (5 mol%)	DMAc	150 °C	16 h	<1	>99<br>(85)	>99<br>(61)
27	2.5	P_4-*t*Bu + K_3PO_4 (5 mol%)	$(EtOCH_2CH_2)_2O$	150 °C	16 h	13	22	6
28	2.5	P_4-*t*Bu + K_3PO_4 (5 mol%)	Xylene	150 °C	16 h	10	13	2
29	2.5	P_4-*t*Bu + K_3PO_4 (5 mol%)	DMAc	65 °C	16 h	15	12	13
30	2.5	P_4-*t*Bu + K_3PO_4 (5 mol%)	DMAc	rt	16 h	2	5	0.5
31	2.1	P_4-*t*Bu + K_3PO_4 (5 mol%)	DMAc	150 °C	16 h	2	79	82
32	2.1	P_4-*t*Bu (5 mol%)<br>+ K_3PO_4 (5 mol%)	DMAc	150 °C	16 h	8	79	75
33	2	KO*t*-Bu + Benzo-18-C-6	DMI	150 °C	16 h	10	90	78

[a] A mixture of **1** (powder, 0.1 mmol relative to the molecular weight of the monomer), **2a** (0.2 mmol), catalyst (0.02 mmol), and solvent (0.2 mL) was stirred for 16 h. Yields were determined by ^1H NMR. Numbers in parentheses are isolated yields.

4. **Supplementary Table S2 |** Decomposition of PSUs having different M_n and M_w.[a]

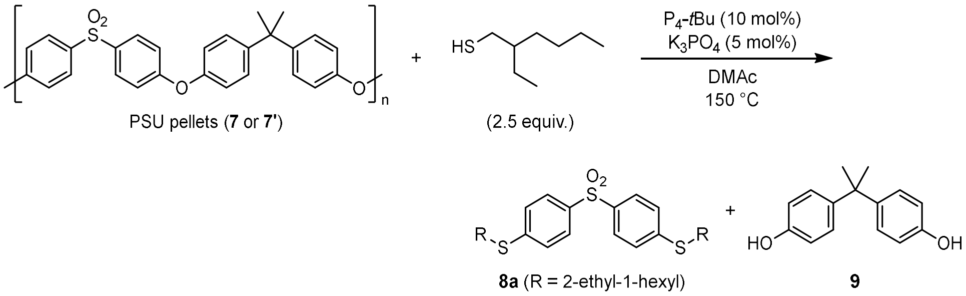

Entry	PSU	Time	**8a** (%)	**9** (%)
1	pellet (M_w 35000, M_n 16000, catalog spec) (**6**)	20 min	50	50
		1 h	79	76
2	pellet (M_w 60000, catalog spec) (**6'**)	20 min	56	53
		1 h	78	73

[a] A mixture of PSU pellets (0.2 mmol relative to the molecular weight of the monomer), 2-ethylhexanethiol (0.5 mmol), P_4-*t*Bu (0.02 mmol), K_3PO_4 (0.01 mmol), and DMAc (0.4 mL) was stirred at 150 °C. Yields were determined by ^{1}H NMR.

5. **Supplementary Table S3 |** Reaction of PSU pellets with 4-*tert*-butylbenzenethiol[a]

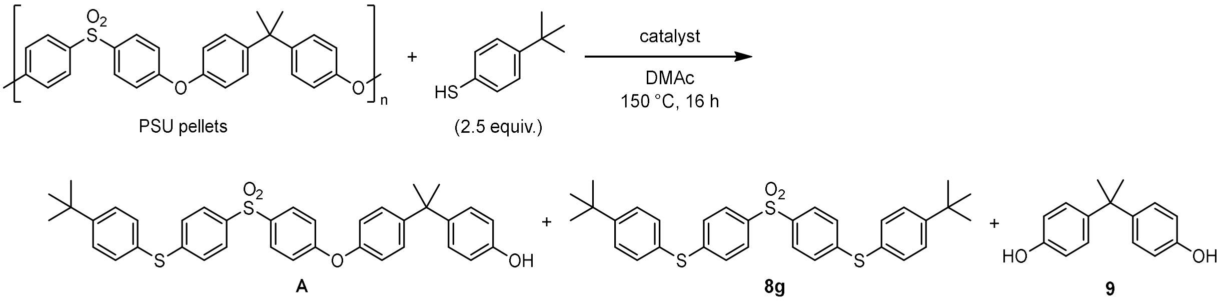

Entry	catalyst	**A** (%)	**8g** (%)	**9** (%)
1	P_4-*t*Bu (10 mol%) K_3PO_4 (5 mol%)	10	90	90
2	NaO*t*Bu (20 mol%)	<1	(98)	(>99)

[a] A mixture of **1** (pellets, 0.1 mmol relative to the molecular weight of the monomer), 4-*tert*-butylbenzenethiol (0.25 mmol), catalyst, and DMAc (0.2 mL) was stirred at 150 °C for 16 h. Yields were determined by ^{1}H NMR. Numbers in parentheses are isolated yields.

6. **Supplementary Table S4 |** Reaction of PEEK powder with 4-*tert*-butylbenzenethiol[a]

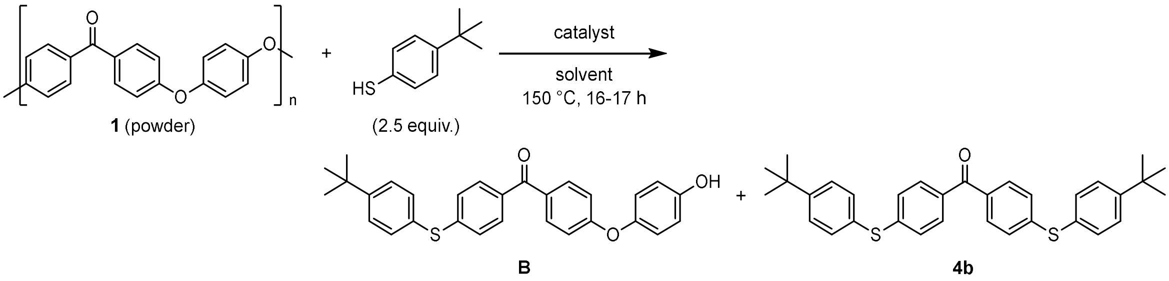

Entry	catalyst	solvent	time	**B** (%)	**4b** (%)
1	NaO*t*Bu (10 mol%)	DMAc	17 h	31	13
2	P_4-*t*Bu (10 mol%)	DMAc	17 h	35	31
3	K_3PO_4 (5 mol%)		64 h	5	60
4	P_4-*t*Bu (20 mol%)<br>K_3PO_4 (10 mol%)	DMAc	64 h	(17)	(58)
5	P_4-*t*Bu (10 mol%)<br>K_3PO_4 (5 mol%)	DMAc[b]	42 h	33	17
6	P_4-*t*Bu (10 mol%)	DMAc[b]	42 h	24	34
7	Cs_2CO_3 (5 mol%)		64 h	35	49
8	P_4-*t*Bu (10 mol%)<br>K_3PO_4 (10 mol%)	DMI[b]	112 h	21	79
9	P_4-*t*Bu (10 mol%)<br>Cs_2CO_3 (5 mol%)	DMI[b]	42 h<br>109 h	37<br>16	47<br>81
10	P_4-*t*Bu (10 mol%)	DMI[b]	42 h	28	72
11[c]	Cs_2CO_3 (10 mol%)		109 h	4	96 (86)

[a] A mixture of **1** (powder, 0.1 mmol relative to the molecular weight of the monomer), 4-*tert*-butylbenzenethiol (0.25 mmol), catalyst, and solvent (0.2 mL) was stirred for 16 h. Yields were determined by ^{1}H NMR. Numbers in parentheses are isolated yields. [b] 1.0 mL. [c] 1,4-Hydroquinone was obtained in 75% NMR yield.

7. Experimental procedures

7-1. Depolymerization-like chemical decomposition of PEEK (1) powder by 2-ethylhexanethiol and a catalytic amount of *t*Bu-P_4 and K_3PO_4 (Table 1, Entry 16, Supplementary Table S1, Entry 26).

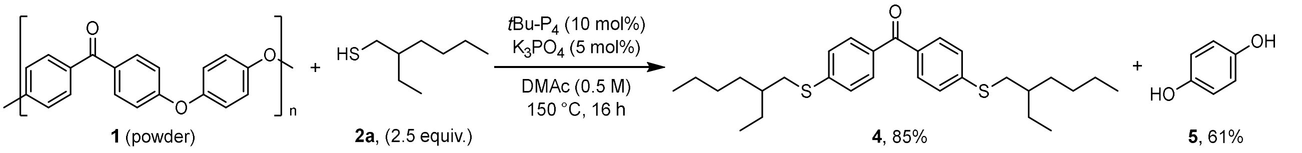

A general procedure for the decomposition/thiolation of super engineering plastics.

To a mixture of PEEK powder (28.8 mg, 0.100 mmol relative to the molecular weight of the monomer), and potassium phosphate tribasic (1.1 mg, 0.0050 mmol) was added *N*,*N*-dimethylacetamide (0.20 mL), P_4-*t*Bu phosphazene base in hexane solution (1-*tert*-butyl-4,4,4-tris(dimethylamino)-2,2-bis[tris(dimethylamino)-phosphoranyliden-amino]-2λ5,4λ5-catenadi(phosphazene), 0.8 M, 0.0125 mL, 0.010 mmol), and 2-ethylhexanethiol (36.6 mg, 0.250 mmol) in a 3 mL vial under argon atmosphere. The resultant mixture was stirred at 150 °C for 16 h. The reaction mixture was cooled to room temperature. The mixture was analyzed by ^{1}H NMR in acetone-d_6 to determine the yields of the products, **4a** and hydroquinone (**5**), using 1,4-dioxane as an internal standard. The reaction mixture was concentrated in vacuo. The crude product was purified by column chromatography on silica gel (hexane/ethyl acetate 96:4 to 7:3) to give bis(4-(2-ethylhexylthio)phenyl)methanone (39.9 mg, 0.085 mmol) and 1,4-hydroquinone (6.7 mg, 0.061 mmol) in 85% and 61% yields, respectively.

As above, other super engineering plastics; PSU, PPSU, PEES, PESU, and PEI, were degraded under the general procedure.

7-2. Chemical decomposition of PSU (7) pellets by 2-phenylethanethiol and a catalytic amount of *t*Bu-P_4 and K_3PO_4 (Fig. 6).

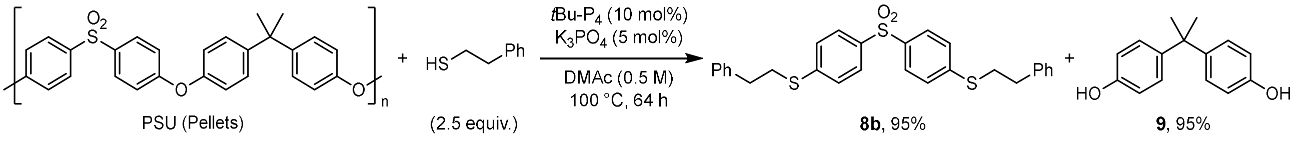

To a mixture of PSU pellets (44.6 mg, 0.101 mmol relative to the molecular weight of the monomer), and potassium phosphate tribasic (1.1 mg, 0.0050 mmol) was added *N*,*N*-dimethylacetamide (0.20 mL), phosphazene base P_4-*t*Bu in hexane solution (1-*tert*-butyl-4,4,4-tris(dimethylamino)-2,2-bis[tris(dimethylamino)-phosphoranylidenamino]-2λ5,4λ

5-catenadi(phosphazene), 0.8 M, 0.0125 mL, 0.010 mmol), and 2-phenylethanethiol (35.0 mg, 0.25 mmol) in a 3 mL vial under argon atmosphere. The resultant mixture was stirred at 150 °C for 16 h. The reaction mixture was cooled to room temperature. The reaction mixture was concentrated in vacuo. The crude product was purified by column chromatography on silica gel (hexane/ethyl acetate 96:4 to 7:3) to give bis(4-(phenethylthio)phenyl)methanone (**8b**) (46.9 mg, 0.096 mmol) and 22-bis(4-hydroxyphenyl) propane (**9**) (21.9 mg, 0.096 mmol) in both 95% yields.

7-3. Chemical decomposition of PSU (7) pellets by 4-*tert*-butylbenzenethiol and a catalytic amount of NaO*t*Bu (Fig. 6).

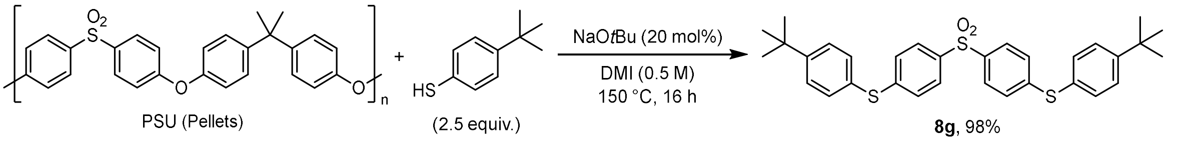

To a mixture of PSU pellets (44.3 mg, 0.10 mmol relative to the molecular weight of the monomer), and sodium *tert*-butoxide (2.0 mg, 0.020 mmol) was added *N*,*N*-dimethylacetamide (0.2 mL) and 4-*tert*-butylbenzenethiol (41.4 mg, 0.25 mmol) in a 4 mL vial under argon atmosphere. The resultant mixture was stirred at 150 °C for 16 h. The reaction mixture was cooled to room temperature and concentrated in vacuo. The crude product was purified by column chromatography on silica gel (hexane/ethyl acetate 96:4 to 7:3) to give bis(4-(*tert*-butylphenylthio)phenyl)sulfone (**8g**) (53.8 mg, 0.098 mmol) and 2,2-bis(4-hydroxyphenyl) propane (**9**) (23.2 mg) in 98% and >99% yields, respectively.

7-4. Chemical decomposition of PEEK (1) powder by 4-*tert*-butylbenzenethiol and a catalytic amount of P_4-*t*Bu and Cs_2CO_3 (Fig. 6).

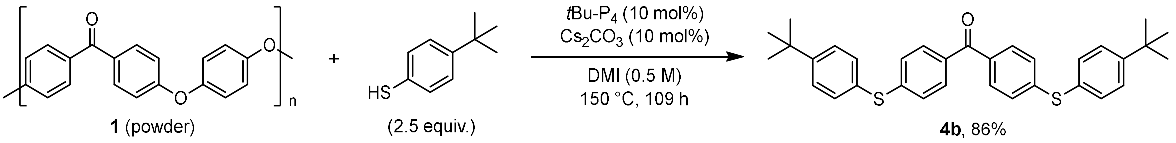

To a mixture of PEEK powder (29.0 mg, 0.101 mmol relative to the molecular weight of the monomer), and cesium carbonate (4.34 mg, 0.013 mmol) was added 1,3-dimethyl-2-imidazolidinone (1.0 mL), phosphazene base P_4-*t*Bu in hexane solution (1-*tert*-butyl-4,4,4-tris(dimethylamino)-2,2-bis[tris(dimethylamino)-phosphoranylidenamino]-2λ5,4λ 5-catenadi(phosphazene), 0.8 M, 0.0125 mL, 0.010 mmol), and 4-*tert*-butylbenzenethiol (40.5 mg, 0.24 mmol) in a 4 mL vial under argon atmosphere. The resultant mixture was stirred at 150 °C for 109 h. The reaction mixture was cooled to room temperature and concentrated in vacuo. The crude

product was purified by column chromatography on silica gel (hexane/ethyl acetate 96:4 to 7:3) to give bis(4-(*tert*-butylphenylthio)phenyl)methanone (**4b**) (44.1 mg, 0.0863 mmol) in 86% yield.

7-5. Gram scale chemical decomposition of PSU (7) pellets by cyclopentanethiol and a catalytic amount of P_4-*t*Bu and K_3PO_4 (Fig. 7a).

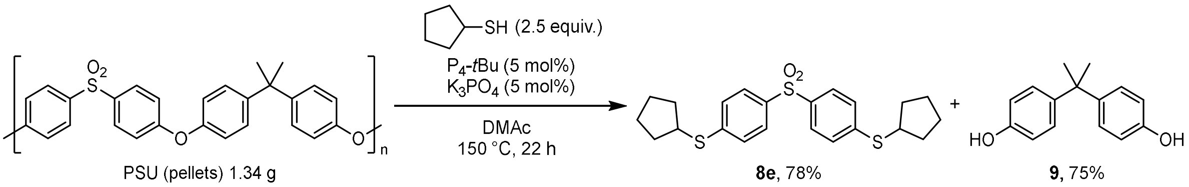

To a mixture of PSU pellets (1.34 g, 3.04 mmol relative to the molecular weight of the monomer), and potassium phosphate tribasic (33.0 mg, 0.16 mmol) was added *N*,*N*-dimethylacetamide (6.0 mL), phosphazene base P_4-*t*Bu in hexane solution (1-*tert*-butyl-4,4,4-tris(dimethylamino)-2,2-bis[tris(dimethylamino)-phosphoranylidenamino]-2λ5,4λ 5-catenadi(phosphazene), 0.8 M, 0.190 mL, 0.15 mmol), and cyclopentanethiol (0.80 mL, 764 mg, 7.5 mmol) in a 20 mL test tube under argon atmosphere. The resultant mixture was stirred at 150 °C for 22 h. The reaction mixture was cooled to room temperature. The reaction mixture was concentrated in vacuo. The crude product was purified by column chromatography on silica gel (hexane/ethyl acetate 93:7 to 7:3 and toluene) to give bis(4-(cyclopentylthio)phenyl)sulfone (**8e**) (991 mg, 2.37 mmol) and 2,2-bis(4-hydroxyphenyl)propane (**9**) (522 mg, 2.09 mmol) in 78% and 75% yields, respectively.

7-6. Chemical decomposition of 30 wt% carbon fiber reinforced PEEK (roughly ground) by 2-ethylhexanethiol and a catalytic amount of P_4-*t*Bu and K_3PO_4 (Fig. 7b).

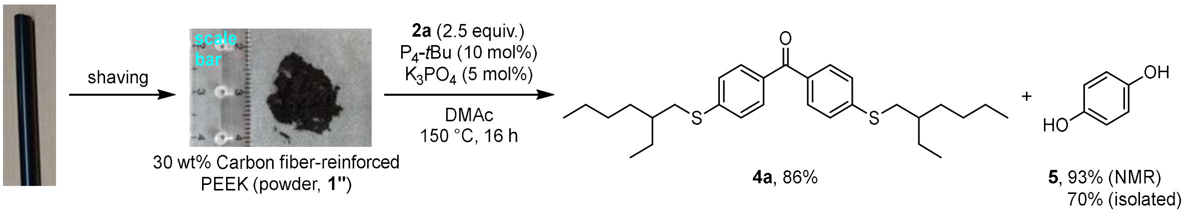

To a mixture of roughly ground 30 wt% carbon fiber-reinforced PEEK (41.2 mg, 0.100 mmol relative to the molecular weight of the monomer), and potassium phosphate tribasic (1.1 mg, 0.0050 mmol) was added *N*,*N*-dimethylacetamide (0.20 mL), phosphazene base P_4-*t*Bu in hexane solution (1-*tert*-butyl-4,4,4-tris(dimethylamino)-2,2-bis[tris(dimethylamino)-phosphoranylidenamino]-2λ5, 4λ5-catenadi(phosphazene), 0.8 M, 0.0125 mL, 0.010 mmol), and 2-ethylhexanethiol (43.0 µL, 0.250 mmol) in a 4 mL vial under argon atmosphere. The resultant mixture was stirred at 150 °C for 16 h. The reaction mixture was cooled to room temperature. This mixture was analyzed by ^{1}H NMR

to determine the yields of products **4a** and **5** in 98% and 93% yields, respectively, by using acetone-d_6 and 1,4-dioxane as an internal standard. The reaction mixture was concentrated in vacuo. The crude product was purified by column chromatography on silica gel (hexane/ethyl acetate 96:4 to 7:3) to give bis(4-(2-ethylhexylthio)phenyl)methanone (40.5 mg, 0.0860 mmol) and hydroquinone (7.7 mg, 0.0700 mmol) in 86% and 70% yields, respectively.

The same decomposition was examined using 30 wt% glass fiber-reinforced PEEK (roughly ground). As a result, **4a** was isolated in 76% yield together with the formation of **5** in 89% NMR yield.

7-7. Chemical decomposition of small pieces of baby bottle made up of PPSU by 2-ethylhexanethiol and a catalytic amount of P_4-*t*Bu and K_3PO_4 (Fig. 7b).

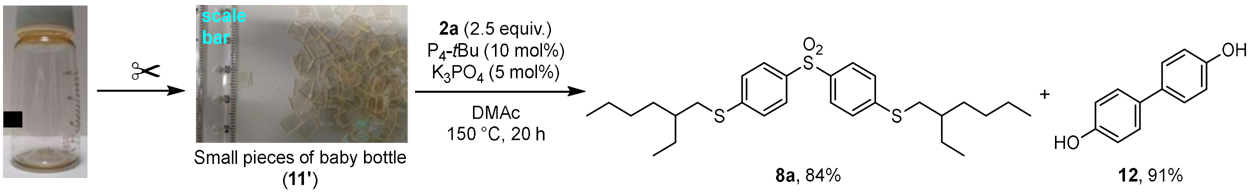

To a mixture of PPSU small pieces derived from a baby bottle (81.4 mg, 0.203 mmol relative to the molecular weight of the monomer), and potassium phosphate tribasic (2.12 mg, 0.010 mmol) was added *N*,*N*-dimethylacetamide (0.40 mL), phosphazene base P_4-*t*Bu in hexane solution (1-*tert*-butyl-4,4,4-tris(dimethylamino)-2,2-bis[tris(dimethylamino)-phosphoranylidenamino]-2λ5,4λ 5-catenadi(phosphazene), 0.8 M, 0.025 mL, 0.020 mmol), and 2-ethylhexanethiol (86.0 μL, 0.500 mmol) in a 4 mL vial under argon atmosphere. The resultant mixture was stirred at 150 °C for 20 h. The reaction mixture was cooled to room temperature. The reaction mixture was concentrated in vacuo. The crude product was purified by column chromatography on silica gel (hexane/ethyl acetate 96:4 to 7:3) to give bis(4-(2-ethylhexylthio)phenyl)methanone (**8a**, 86.7 mg, 0.0171 mmol) and 4,4'-dihydroxybiphenyl (**12**, 34.3 mg, 0.0184 mmol) in 84% and 91% yields, respectively.

7-8. Cross-coupling of 8e with *p-norm*-decylaniline (Fig. 8a).[S2]

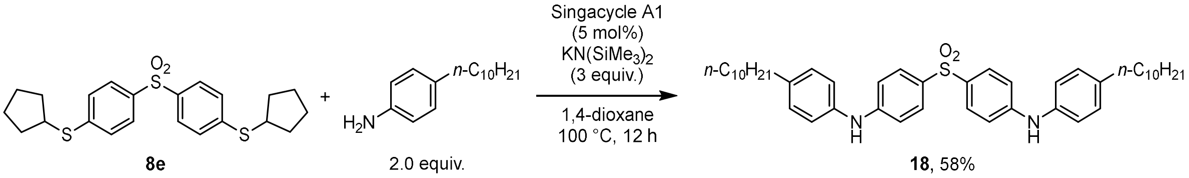

To a mixture of Chloro[[1,3-bis(2,6-diisopropylphenyl)imidazol-2-ylidene](*N*,*N*-dimethylbenzyl-amine)palladium(II)] (SingaCycle-A1, 3.3 mg, 0.0050 mmol), **8e** (41.4 mg, 0.10 mmol), and potassium bistrimethylsilylamide (64.0 mg, 0.32 mmol) was added 1,4-dioxane (2.0 mL) and

4-decylaniline (47.6 mg, 0.204 mmol) in a 4 mL vial under argon atmosphere. The resultant mixture was stirred at 100 °C for 12 h. Water was added to the reaction mixture and the organic layer was extracted with ethyl acetate and washed by brine. The organic layer was concentrated in vacuo. The crude product was purified by column chromatography on silica gel (hexane/ethyl acetate, 94:6 to 50:50) an eluent to give the desired product, 4,4'-sulfonylbis(*N*-(4-decylphenyl)aniline) (**18**) (38.7 mg, 0.057 mmol) in 58% yield.

7-9. Disulfonation of 4b (Fig. 8b left).[S3]

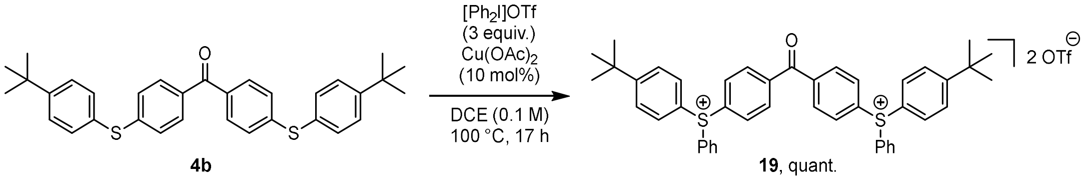

To a mixture of **4b** (103 mg, 0.202 mmol), copper diacetate (3.67 mg, 0.020 mmol), and diphenyliodonium trifluoromethanesulfonate (261 mg, 0.606 mmol) was added 1,2-dichloroethane (2.0 mL) in a 4 mL vial under argon atmosphere. The resultant mixture was stirred at 100 °C for 17 h. The reaction mixture was filtered through Celite, and the filtrate was concentrated in vacuo. The obtained crude oil was washed with hexane. The desired product, (carbonylbis(4,1-phenylene))-bis((4-(*tert*-butyl)phenyl)(phenyl)sulfonium) bis(trifluoromethane-sulfonate) (**19**) was obtained quantitatively (194 mg, 0.202 mmol) including a trace amount of inseparable impurities.

7-10. Diflorination of 19 (Fig. 8b right).[S4]

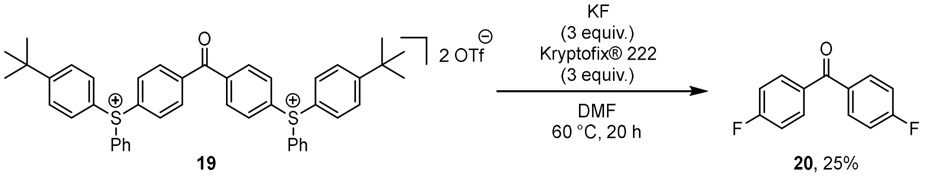

To a mixture of **19** (194 mg, 0.202 mmol), potassium fluoride (35.0 mg, 0.602 mmol), and Kryptofix@ 222 (227 mg, 0.602 mmol) was added *N*,*N*-dimethylformamide (2.0 mL) in a 4 mL vial under argon atmosphere. The resultant mixture was stirred at 60 °C for 20 h. The reaction mixture was concentrated in vacuo. The crude product was purified by column chromatography on silica gel (hexane/ethyl acetate, 100:0 to 90:10) an eluent followed by HPLC to afford 4,4’-difluoro-benzophenone (**20**) (11.1 mg, 0.051 mmol) in 25% yield.

7-11. Disulfonation of 8g (Fig. 8c left).[S3]

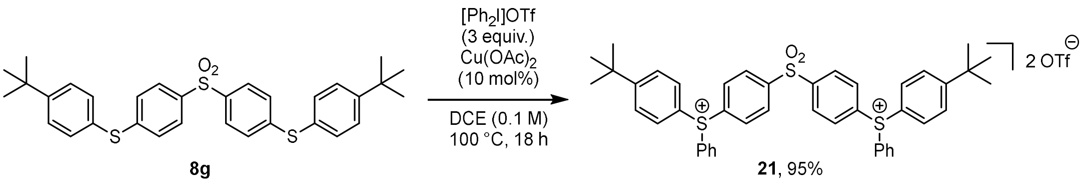

To a mixture of **8g** (202 mg, 0.37 mmol), copper diacetate (6.8 mg, 0.037 mmol), and diphenyliodonium trifluoromethanesulfonate (477 mg, 1.11 mmol) was added 1,2-dichloroethane (3.7 mL) in a 4 mL vial under argon atmosphere. The resultant mixture was stirred at 100 °C for 18 h. After ethyl acetate and hexane were added, the generated oil was separated from the solution. The obtained crude oil was washed with hexane. Then the desired product, (sulfonylbis(4,1-phenylene))bis((4-(tert-butyl)phenyl)(phenyl)sulfonium) bis(trifluoromethane-sulfonate) **21** was obtained quantitatively (350 mg, 0.35 mmol) in 95% yield.

7-12. Diflorination of 21 (Fig. 8c right-upper).[S5]

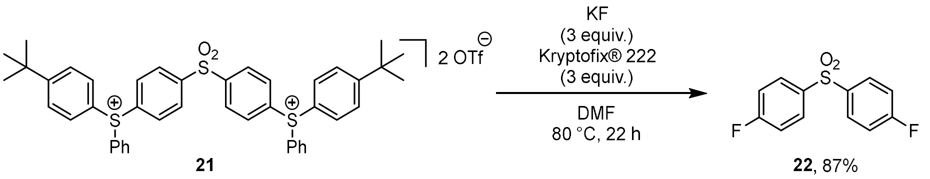

To a mixture of **21** (99.7 mg, 0.100 mmol), potassium fluoride (17.7 mg, 0.30 mmol), and Kryptofix@ 222 (114 mg, 0.30 mmol) was added *N*,*N*-dimethylformamide (0.2 mL) in a 4 mL vial under argon atmosphere. The resultant mixture was stirred at 80 °C for 22 h. The reaction mixture was filtered through celite and concentrated in vacuo. The crude product was purified by preparative thin layer chromatography on silica gel (hexane/ethyl acetate, 8:2) to afford 4,4'-difluorodibenzosulfone (**22**) (22.1 mg, 0.087 mmol) in 87% yield.

7-13. Dietherification of 21 (Fig. 8c right-lower).[S6]

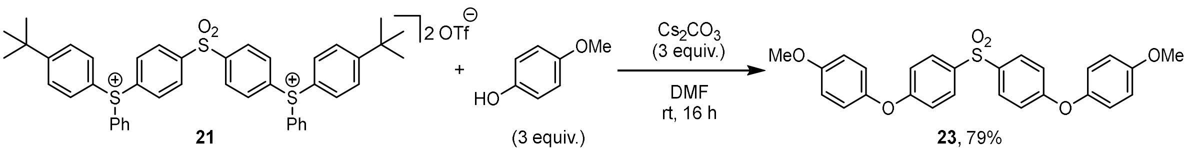

To a mixture of **21** (100 mg, 0.100 mmol), *p*-methoxyphenol (39.0 mg, 0.31 mmol), and cesium carbonate (97.7 mg, 0.30 mmol) was added *N*,*N*-dimethylformamide (0.5 mL) in a 4 mL vial under argon atmosphere. The resultant mixture was stirred at room temperature for 16 h. The reaction mixture was cooled to room temperature. The reaction mixture was subjected to purification by column chromatography on silica gel (hexane/ethyl acetate, 92:8 to 34:66) as an eluent to afford 4,4'-sulfonylbis((4-methoxyphenoxy)benzene) (**23**) (36.6 mg, 0.080 mmol) in 79% yield.

8. Spectrum data of the products.

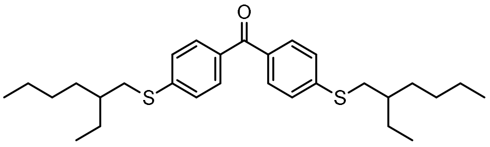

Bis(4-(2-ethyl-*n*-hexylthio)phenyl)methanone (4a). A yellow oil. Rf = 0.40 (hexane/AcOEt = 10/1 (v/v)). ^{1}H NMR (600 MHz, $CDCl_3$) δ 0.89-0.93 (m, 12H, methyl), 1.29-1.31 (m, 8H, methylene), 1.39-1.53 (m, 8H, methylene), 1.64 (sept, *J* = 6.2 Hz, 2H, methyne), 2.98 (d, *J* = 6.4 Hz, 4H, SCH_2), 7.32 (d, *J* = 8.4 Hz, 4H, aromatic), 7.69 (d, *J* = 8.4 Hz, 4H, aromatic). ^{13}C NMR (151 MHz, $CDCl_3$) δ 10.8, 14.1, 23.0, 25.7, 28.8, 32.5, 36.5, 38.7, 126.3, 130.4, 134.1, 144.6, 195.0. IR (neat) 2958, 2926, 2858, 1651, 1589, 1459, 1398, 1312, 1287, 1179, 1088, 926, 845, 755, 673 cm^{-1}. HRMS calcd for $C_{29}H_{42}OS_2Na$ (M + Na) 493.2575, found 493.2569.

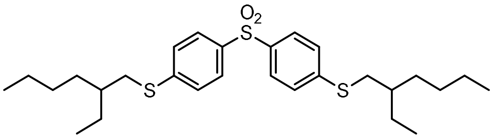

Bis(4-(2-ethyl-*n*-hexylthio)phenyl)sulfone (8a). A colorless oil. Rf = 0.55 (hexane/AcOEt = 10/1 (v/v)). ^{1}H NMR (600 MHz, $CDCl_3$) δ 0.87-0.90 (m, 12H, methyl), 1.24-1.31 (m, 8H, methylene), 1.36-1.49 (m, 8H, methylene), 1.61 (sept, *J* = 6.3 Hz, 2H, methyne), 2.92 (d, *J* = 6.4 Hz, 4H, SCH_2), 7.30 (AA'BB', 4H, aromatic), 7.77 (AA'BB', 4H, aromatic). ^{13}C NMR (151 MHz, $CDCl_3$) δ 10.7, 14.1, 22.9, 25.7, 28.7, 32.4, 36.3, 38.6, 126.7, 127.7, 137.6, 146.2. IR (neat) 2958, 2927, 2858, 1577, 1458, 1394, 1320, 1159, 1077, 819, 764, 626 cm^{-1}. HRMS calcd for $C_{28}H_{42}O_2S_3Na$ (M + Na) 529.2239, found 529.2222.

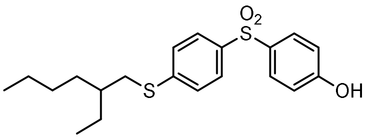

4-((4-((2-Ethylhexyl)thio)phenyl)sulfonyl)phenol (14). A pale yellow oil. Rf = 0.40 (hexane/AcOEt = 1/1 (v/v)). ^{1}H NMR (600 MHz, $CDCl_3$) δ 0.88 (t, *J* = 7.0 Hz, 3H, methyl), 0.89 (t, *J* = 7.5 Hz, 3H, methyl), 1.25-1.29 (m, 4H, methylene), 1.37-1.48 (m, 4H, methylene), 1.61 (sept, *J* = 6.3 Hz, 1H, methyne), 2.92 (d, *J* = 6.2 Hz, 2H, SCH_2), 6.89 (AA'BB', 2H, aromatic), 7.30 (AA'BB', 2H, aromatic), 7.76 (AA'BB', 2H, aromatic), 7.80 (AA'BB', 2H, aromatic). ^{13}C NMR (151 MHz, $CDCl_3$) δ 10.7, 14.1, 22.9, 25.7, 28.7, 32.4, 36.3, 38.6, 116.0, 126.7, 127.6, 130.0, 133.6, 137.9, 146.0, 159.8. IR (neat) 3363, 2959, 2926, 2857, 1585, 1500, 1439, 1301, 1148, 1111, 1083, 837, 757,

673 cm^{-1}. HRMS calcd for $C_{20}H_{26}O_3S_2Na$ (M + Na) 401.1216, found 401.1209.

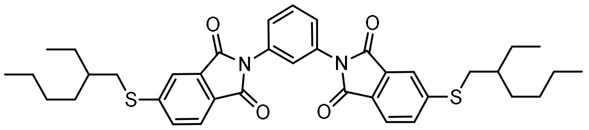

2,2'-(1,3-Phenylene)bis(5-((2-ethylhexyl)thio)isoindoline-1,3-dione) (17). A yellow oil. Rf = 0.30 (hexane/AcOEt = 10/1 (v/v)). ^{1}H NMR (600 MHz, $CDCl_3$) δ 0.90-0.95 (m, 12H, methyl), 1.30-1.34 (m, 8H, methylene), 1.43-1.53 (m, 8H, methylene), 1.68 (sept, *J* = 6.4 Hz, 2H, methyne), 3.04 (d, *J* = 6.4 Hz, 4H, SC*H*2), 7.52 (dd, *J* = 2.0, 8.0 Hz, 2H, aromatic), 7.59 (dd, *J* = 1.7, 7.9 Hz, 2H, aromatic), 7.62-7.63 (m, 1H, aromatic), 7.67 (t, *J* = 2.0 Hz, 1H, aromatic), 7.76 (d, *J* = 1.3 Hz, 2H, aromatic), 7.79 (dd, *J* = 0.26, 7.9 Hz, 2H, aromatic). ^{13}C NMR (151 MHz, $CDCl_3$) δ 10.8, 14.1, 22.9, 25.7, 28.8, 32.5, 36.7, 38.6, 120.7, 123.78, 123.85, 125.4, 127.3, 129.4, 131.8, 132.4 (two signals), 148.7, 166.6, 166.7. IR (neat) 2958, 2930, 2858, 1773, 1718, 1606, 1495, 1456, 1424, 1354, 1179, 1103, 910, 743, 667, 633 cm^{-1}. HRMS calcd for $C_{38}H_{44}N_2O_4S_2Na$ (M + Na) 679.2635, found 679.2607.

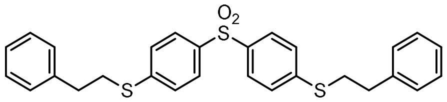

Bis(4-(2-phenylethylthio)phenyl)sulfone (8b). A pale yellow solid. Rf = 0.20 (hexane/AcOEt = 10/1 (v/v)). m.p. = 94-100 °C. ^{1}H NMR (600 MHz, $CDCl_3$) δ 2.96 (t, *J* = 7.9 Hz, 4H, methylene), 3.22 (d, *J* = 7.9 Hz, 4H, SC*H*2), 7.20 (d, *J* = 7.0 Hz, 4H, aromatic), 7.22-7.24 (m, 2H, aromatic), 7.30-7.33 (m, 8H, aromatic), 7.79 (AA'BB', 4H, aromatic). ^{13}C NMR (151 MHz, $CDCl_3$) δ 33.5, 35.0, 126.8, 126.9, 127.9, 128.5, 128.7, 138.0, 139.4, 145.0. IR (neat) 2922, 2854, 1575, 1454, 1306, 1156, 1077, 817 cm^{-1}. HRMS calcd for $C_{28}H_{26}O_2S_3Na$ (M + Na) 513.0987, found 513.1025.

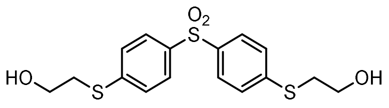

Bis(4-(2-hydroxyethylthio)phenyl)sulfone (8c). A colorless oil. Rf = 0.40 (AcOEt). ^{1}H NMR (600 MHz, acetone-*d*6) δ 3.21 (t, *J* = 6.6 Hz, 4H, SC*H*2), 3.76-3.78 (m, 4H, HOC*H*2), 4.12 (br, 2H, OH), 7.50 (AA'BB', 4H, aromatic), 7.84 (AA'BB', 4H, aromatic). ^{13}C NMR (151 MHz, acetone-*d*6) δ 35.0, 61.1, 127.7, 128.7, 139.2, 146.3. IR (neat) 3423, 2929, 1733, 1574, 1396, 1306, 1151, 1091, 1078, 1007, 823, 765, 630cm^{-1}. HRMS calcd for $C_{16}H_{18}O_4S_3Na$ (M + Na) 393.0259, found 393.0251.

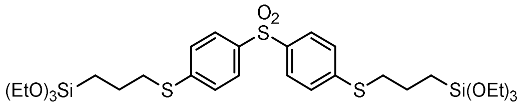

Bis(4-(3-(triethoxysilyl)propylthio)phenyl)sulfone (8d). A pale brown oil. ^{1}H NMR (600 MHz, $CDCl_3$) δ 0.75-0.78 (m, 4H, methylene), 1.19 (t, J = 7.0 Hz, 18H, methyl), 1.79 (tt, J = 7.7 Hz, 4H, methylene), 2.99 (t, J = 7.5 Hz, 4H, SCH_2), 3.79 (q, J = 7.0 Hz, 12H, OCH_2), 7.30 (AA'BB', 4H, aromatic), 7.76 (AA'BB', 4H, aromatic). ^{13}C NMR (151 MHz, $CDCl_3$) δ 9.93, 18.3, 22.4, 34.6, 58.5, 126.7, 127.8, 137.7, 145.5. IR (neat) 2923, 1577, 1315, 115, 1077, 765, 623 cm^{-1}. HRMS calcd for $C_{30}H_{50}O_8S_3Si_2Na$ (M + Na) 713.2283, found 713.2303.

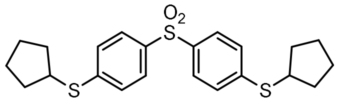

Bis(4-(cyclopentylthio)phenyl)sulfone (8e). A colorless solid. Rf = 0.30 (hexane/AcOEt = 10/1 (v/v)). m.p. = 116-118 °C. ^{1}H NMR (600 MHz, $CDCl_3$) δ 1.59-1.67 (m, 8H, methylene), 1.75-1.81 (m, 4H, methylene), 2.09-2.16 (m, 4H, methylene), 3.65-3.70 (m, 2H, SCH), 7.32 (AA'BB', 4H, aromatic), 7.77 (AA'BB', 4H, aromatic). ^{13}C NMR (151 MHz, $CDCl_3$) δ 24.9, 33.4, 44.1, 127.2, 127.7, 137.6, 146.2. IR (neat) 2951, 1577, 1478, 1396, 1317, 1158, 1119, 1089, 1075, 820, 763, 744, 630 cm^{-1}. HRMS calcd for $C_{22}H_{26}O_2S_3Na$ (M + Na) 441.0987, found 441.0967.

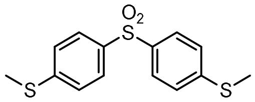

(Sulfonylbis(4,1-phenylene))bis(methylsulfane) (8f). A yellow solid. Rf = 0.10 (hexane/AcOEt = 10/1 (v/v)). m.p. = 131-133 °C. ^{1}H NMR (600 MHz, $CDCl_3$) δ 2.49 (s, 6H, methyl), 7.27 (AA'BB', 4H, aromatic), 7.79 (AA'BB', 4H, aromatic). ^{13}C NMR (151 MHz, $CDCl_3$) δ 14.8, 125.5, 127.8, 137.5, 146.5. IR (neat) 2922, 1575, 1476, 1395, 1310, 1152, 1115, 1081, 820, 763, 746, 724, 623 cm^{-1}. HRMS calcd for $C_{14}H_{14}O_2S_3Na$ (M + Na) 333.0048, found 333.0043.

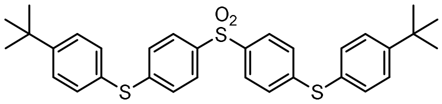

(Sulfonylbis(4,1-phenylene))bis((4-(tert-butyl)phenyl)sulfane) (8g). A colorless oil. Rf = 0.45 (hexane/AcOEt = 10/1 (v/v)). ^{1}H NMR (600 MHz, $CDCl_3$) δ 1.34 (s, 18H, *t*Bu), 7.14 (AA'BB', 4H, aromatic), 7.40-7.44 (m, 8H, aromatic), 7.70 (AA'BB', 4H, aromatic). ^{13}C NMR (151 MHz, $CDCl_3$)

δ 31.2, 34.8, 126.9, 126.99, 127.05, 127.9, 134.5, 138.1, 146.7, 152.9. IR (neat) 2962, 1576, 1476, 1393, 1316, 1156, 1112, 1073, 1011, 821, 762, 734, 625 cm^{-1}. HRMS calcd for $C_{32}H_{34}O_2S_3Na$ (M + Na) 569.1613, found 569.1612.

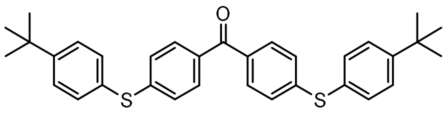

Bis(4-(*tert*-butylphenylthio)phenyl)methanone (4b). A brown solid. Rf = 0.45 (hexane/AcOEt = 10/1 (v/v)). mp = 145 – 148 °C. ^{1}H NMR (600 MHz, $CDCl_3$) δ 1.34 (s, 18H, *t*Bu), 7.19 (AA'BB', 4H, aromatic), 7.42-7.46 (m, 8H, aromatic), 7.64 (AA'BB', 4H, aromatic). ^{13}C NMR (151 MHz, $CDCl_3$) δ 31.3, 34.8, 126.7, 126.8, 128.1, 130.6, 134.0, 134.5, 144.9, 152.4, 194.9. IR (neat) 2961, 1646, 1588, 1488, 1401, 1310, 1288, 1177, 1081, 1014, 928, 848, 834, 801, 757, 671 cm^{-1}. HRMS calcd for $C_{33}H_{34}OS_2Na$ (M + Na) 533.1949, found 533.1943.

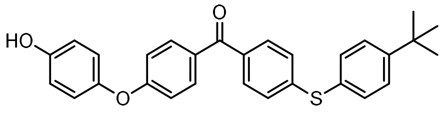

(4-((4-(Tert-butyl)phenyl)thio)phenyl)(4-(4-hydroxyphenoxy)phenyl)methanone (3b). A brown oil. Rf = 0.30 (hexane/AcOEt = 10/1 (v/v)). ^{1}H NMR (600 MHz, $CDCl_3$) δ 1.34 (s, 9H, *t*Bu), 5.12 (br, 1H, OH), 6.86 (AA'BB', 2H, aromatic), 6.95 (AA'BB', 2H, aromatic), 6.97 (AA'BB', 2H, aromatic), 7.20 (AA'BB', 2H, aromatic), 7.42-7.46 (m, 4H, aromatic), 7.65 (AA'BB', 2H, aromatic), 7.75 (AA'BB', 2H, aromatic). ^{13}C NMR (151 MHz, $CDCl_3$) δ 31.3, 34.8, 116.2, 116.6, 121.9, 126.77, 126.83, 128.2, 130.6, 131.4, 132.4, 134.0, 134.8, 144.7, 148.6, 152.3, 152.7, 162.5, 194.8. IR (neat) 2961, 1646, 1590, 1499, 1312, 1238, 1167, 1083, 1013, 929, 831, 760, 677 cm^{-1}. HRMS calcd for $C_{29}H_{26}O_3SNa$ (M + Na) 477.1500, found 477.1495.

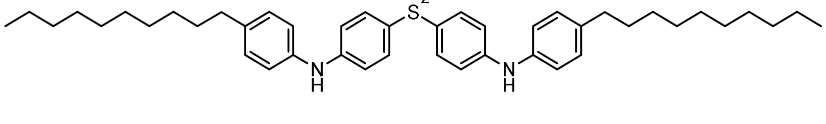

4,4'-Sulfonylbis(*N*-(4-decylphenyl)aniline) (18). A colorless solid. Rf = 0.16 (hexane/AcOEt = 3/1 (v/v)). m.p. = 123-129 °C. ^{1}H NMR (600 MHz, $CDCl_3$) δ 0.88 (t, *J* = 6.9 Hz, 6H, methyl), 1.26-1.31 (m, 28H, methylene), 1.60 (tt, *J* = 7.5, 7,0 Hz, 4H, methylene), 2.57 (t, *J* = 7.6 Hz, 4H, methylene), 5.97 (br, 2H, NH), 6.91 (d, *J* = 8.8 Hz, 4H, aromatic), 7.05 (d, *J* = 8.3 Hz, 4H, aromatic), 7.14 (d, *J* = 8.3 Hz, 4H, aromatic), 7.71 (d, *J* = 8.8 Hz, 4H, aromatic). ^{13}C NMR (151 MHz, $CDCl_3$) δ 14.1, 22.7, 29.3, 29.4, 29.5, 29.62, 29.64, 31.6, 31.9, 35.4, 114.4, 121.6, 129.2, 129.5, 131.8, 137.7, 138.9, 148.6.

IR (neat) 3344, 2956, 2921, 2851, 1594, 1520, 1354, 1281, 816, 740, 641, 612 cm^{-1}. HRMS calcd for $C_{36}H_{60}O_2SN_2Na$ (M + Na) 703.4268, found 703.4274.

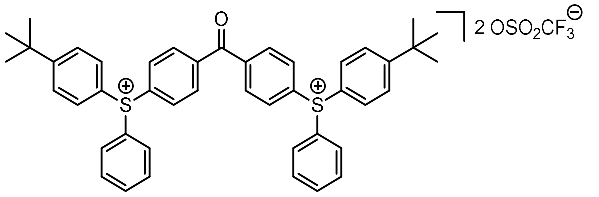

(Carbonylbis(4,1-phenylene))bis((4-(tert-butyl)phenyl)(phenyl)sulfonium) bis(trifluormethane-sulfonate) (19). A brown oil. H NMR (600 MHz, $CDCl_3$) δ 1.35 (s, 18H, *t*Bu), 7.73-7.76 (br-m, 12H, aromatic), 7.78-7.81 (br, 10H, aromatic), 8.11-8.12 (br, 4H, aromatic). ^{13}C NMR (151 MHz, $CDCl_3$) δ 30.8, 35.5, 119.7, 120.7 (q, J = 321 Hz), 124.0, 129.2, 129.5, 131.1, 131.4, 131.5, 131.9, 132.5, 135.0, 140.6, 159.6, 193.1. IR (neat) 2966, 1672, 1590, 1400, 1259, 1224, 1158, 1070, 1030, 929, 836, 756, 684, 669, 638 cm^{-1}. HRMS calcd for $C_{46}H_{44}O_4F_3S_3$ (M - OTf) 813.2348, found 813.2335.

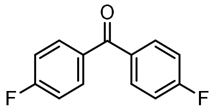

4,4'-Carbonylbis(fluorobenzene) (20).[S5] Known chemical (CAS registry number: 345-92-6). ^1H NMR (600 MHz, $CDCl_3$) δ 7.16-7.18 (m, 4H, aromatic), 7.80-7.83 (m, 4H, aromatic).

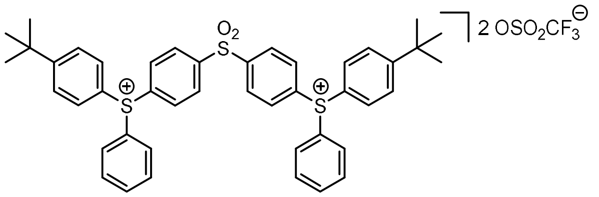

(Sulfonylbis(4,1-phenylene))bis((4-(tert-butyl)phenyl)(phenyl)sulfonium) bis(trifluoromethane-sulfonate) (21). A brown oil. ^1H NMR (600 MHz, $CDCl_3$) δ 1.33 (s, 18H, *t*Bu), 7.71-7.81 (br-m, 22H, aromatic), 8.20 (br, 4H, aromatic). ^{13}C NMR (151 MHz, $CDCl_3$) δ 30.9, 35.6, 119.3, 120.7 (q, J = 319 Hz), 123.7, 129.3, 130.8, 131.5, 131.7, 131.77, 131.79, 132.0, 135.2, 144.8, 159.9. IR (neat) 3096, 2960, 1396, 1258, 1225, 1161, 1029, 1008, 836, 766, 637, 619 cm^{-1}. HRMS calcd for $C_{45}H_{42}O_5F_3S_4$ (M - OTf) 849.2018, found 849.1980.

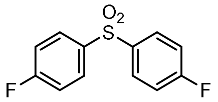

4,4'-Sulfonylbis(fluorobenzene) (22).[S5] Known chemical (CAS registry number: 383-29-9). ^{1}H NMR (600 MHz, $CDCl_3$) δ 7.17-7.21 (m, 4H, aromatic), 7.93-7.96 (m, 4H, aromatic).

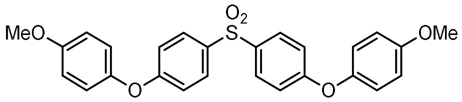

4,4'-Sulfonylbis((4-methoxyphenoxy)benzene) (23).[S6] Known chemical (CAS registry number: 34018-55-8). ^{1}H NMR (600 MHz, $CDCl_3$) δ 3.82 (s, 6H, OMe), 6.91 (AA'BB', 4H, aromatic), 6.95 (AA'BB', 4H, aromatic), 6.98 (AA'BB', 4H, aromatic), 7.82 (AA'BB', 4H, aromatic). ^{13}C NMR (151 MHz, $CDCl_3$) δ 55.7, 115.2, 116.9, 121.8, 129.7, 135.0, 148.1, 157.0, 162.8.

9. Additional experiments and data

9-1. Time course study

Time courses were run for the PEEK to monitor the yields of the products. Reactions were set up according to the conditions shown below (Supplementary Table S5). After the stirring for appropriate time (0.5 h, 1 h, 3 h or 4 h) at 150 °C, the reaction mixture was analyzed by ^{1}H NMR to determine the yields of **3**, **4a**, and **5**. These experiments were conducted three times in total except of the case using 5 mol% of K_3PO_4. As shown in the manuscript, the catalytic conditions using both P_4-*t*Bu and K_3PO_4 are the most effective for this decomposition reaction. Even after 0.5 h, the total yields **3** and **4a** reached nearly 70%. The reaction rate under the single use of P_4-*t*Bu catalyst (10 mol%) was faster the that of K_3PO_4 catalyst (10 mol%).

Supplementary Table S5 | Yields of products from PEEK decomposition time course.

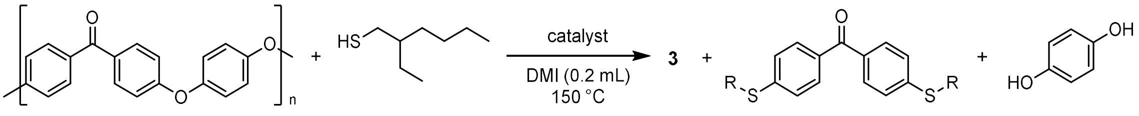

1 ("0.1 mmol", powder) **2a** (2.5 equiv.) **4a** (R = 2-Ethyl-Hexyl) **5**

Entry	Conditions	Time	**4a** (%)	**5** (%)
1	*t*Bu-P_4 (10 mol%) K_3PO_4 (5 mol%)	0.5 h	38,[c] 20,[d] 14[e]	36,[c] 17,[d] 12[e]
		1 h	58,[c] 52,[d] 48[e]	55,[c] 53,[d] 46[e]
		3 h	96,[c] 81,[d] 81[e]	87,[c] 84,[d] 80[e]
2	*t*Bu-P_4 (10 mol%)	0.5 h	32,[c] 11,[d] 12[e]	27,[c] 9,[d] 11[e]
		1 h	41,[c] 46,[d] 40[e]	40,[c] 44,[d] 39[e]
		3 h	71,[c] 79,[d] 73[e]	64,[c] 74,[d] 73[e]
3[f]	K_3PO_4 (10 mol%)	0.5 h	17,[c] 8,[d] 13[e]	16,[c] 7,[d] 11[e]
		1 h	27,[c] 21,[d] 31[e]	25,[c] 21,[d] 32[e]
		4 h	39,[c] 38,[d] 59[e]	40,[c] 40,[d] 61[e]
4[f]	K_3PO_4 (5 mol%)	0.5 h	11[c]	10[c]
		1 h	15[c]	14[c]
		3 h	22[c]	18[c]

[a] Determined by GC. [b] Determined by ^{1}H NMR. [c] 1st run. [d] 2nd run. [e] 3rd run. [f] DMAc was used as a solvent.

9-2. Checking weight change of PEEK samples by solvents at 150 °C

Seven PEEK granules (approximately 2 mm × 3 mm column forms) were used in this experiment. Purchased PEEK plate was cut to form approximately 5 mm × 6 mm × 1 mm small plates which were used in this experiment.

These PEEK samples were added to the solvent (DMAc, DMF, PhCN, $(EtOCH_2CH_2)_2O$, or xylene). The resultant mixture was heated at 150 °C for 19 h. After the decantation, recovered resins were washed with hexane and dried at 115 °C for 2 h. The mass of the recovered resins was measured. The results were shown in Supplementary Table S6 and Fig. S1. All used solvents increased the mass of PEEK resins (105~109 wt%) whereas the resins were apparently unchanged. At least, the increase in mass affected the solvents, but not the morphology of the PEEK sample.

Supplementary Table S6 | Weight change of PEEK samples by solvents at 150 °C. In the pictures, 5 mm grids are shown.

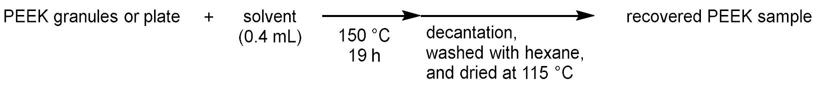

Entry	PEEK sample	weight	solvent	Recovered weight of PEEK sample
1	7 granules	120.4 mg	DMAc	130.9 mg (109 wt%)
2	Plate	57.37 mg	DMAc	62.23 mg (108 wt%)
3	7 granules	111.2 mg	DMF	120.1 mg (108 wt%)
4	Plate	56.78 mg	DMF	61.05 mg (108 wt%)
5	7 granules	111.3 mg	PhCN	121.7 mg (109 wt%)
6	Plate	54.58 mg	PhCN	59.44 mg (109 wt%)
7	7 granules	125.1 mg	$(EtOCH_2CH_2)_2O$	130.8 mg (105 wt%)
8	Plate	59.55 mg	$(EtOCH_2CH_2)_2O$	62.29 mg (105 wt%)
9	7 granules	121.9 mg	xylene	130.3 mg (107 wt%)
10	Plate	57.95 mg	xylene	61.84 mg (107 wt%)

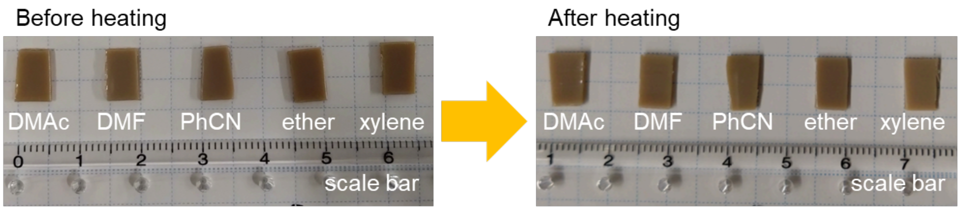

Supplementary Fig. S1 | Photo images of PEEK plates used in the experiment. After heating in solvents, the appearance of each resin did not change.

9-3. NMR and ESI-TOF-MS analyses of the combination of arylthiol, P_4-*t*Bu, and K_3PO_4

To get the information about the combination between thiols and catalysts, we examined the NMR experiments using DMF-d_7 solvent (Supplementary Fig. S2, S3, S4, S5).

Compared to the ^{1}H NMR and ^{13}C NMR chemical of 4-*tert*-butylphenylthiol (**2b**) (Supplementary Fig. S1) with the ones of the combination between **2b** and P_4-*t*Bu (Supplementary Fig. S3), $[P_4\text{-}t\text{Bu-H}]^+\cdot[S(C_6H_4\text{-}t\text{Bu})]^-$ phosphazenium salt was formed by the reaction of **2b** with P_4-*t*Bu (Supplementary Eq. S1).[S7] At ^{31}P NMR, signals (δ 12.7 (d), -23.5 (q)) derived from $[P_4\text{-}t\text{Bu-H}]^+$ were detected. A mass peak was observed at m/z 634 in ESI-TOF-(+)-MS mass spectra, supporting that $[P_4\text{-}t\text{Bu-H}]^+$ was generated.

On the other hand, ^{1}H NMR analysis of **2b** and K_3PO_4 in DMF-d_7 showed two broad peaks (δ 7.21-7.59, 4.99) derived from aryl and thiol groups (Supplementary Fig. S4). The signal of the thiol group was disappeared after 1 h. At least, this result suggests that potassium arylthiolate was generated (Supplementary Eq. S2), whose ^{1}H NMR signals were different from the case in Supplementary Fig. S3.

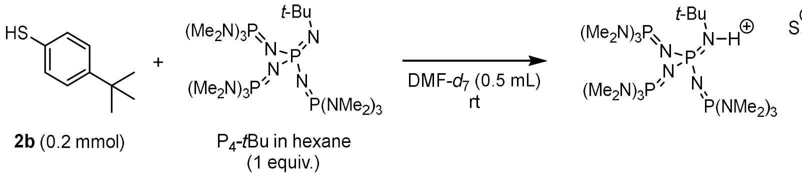

(S1)

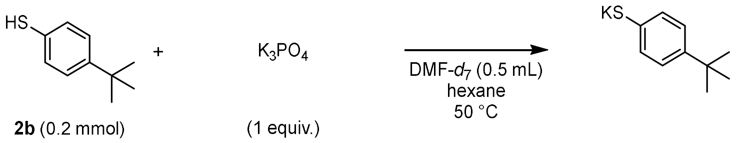

(S2)

Then, we performed the NMR analyses of the reaction of **2b** with P_4-*t*Bu in the presence of K_3PO_4 in DMF-d_7. As shown in Supplementary Fig. S5, chemical shifts of aryl protons in 4-*tert*-butylphenylthiolate at ^{1}H NMR were same as $[P_4\text{-}t\text{Bu-H}]^+\cdot[S(C_6H_4\text{-}t\text{Bu})]^-$ but the signals were broadened. Corresponding ^{13}C signals of aryl carbons at ^{13}C NMR were also broadened. However, two $[P_4\text{-}t\text{Bu-H}]^+$ signals were detected as in Supplementary Fig. S3 at ^{31}P NMR. A mass peak was also observed at m/z 634 in ESI-TOF-(+)-MS and m/z 165 in ESI-TOF-(-)-MS mass spectra, supporting that $[P_4\text{-}t\text{Bu-H}]^+\cdot[S(C_6H_4\text{-}t\text{Bu})]^-$ was generated. These results indicated that soluble $[P_4\text{-}t\text{Bu-H}]^+\cdot[S(C_6H_4\text{-}t\text{Bu})]^-$ was initially formed and $[S(C_6H_4\text{-}t\text{Bu})]$ anion interacted with K_3PO_4 in the equilibrium state (Supplementary Eq. S4).

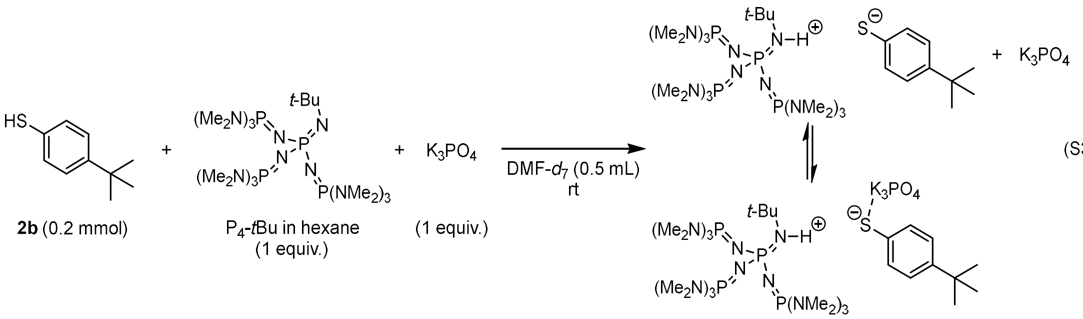

(S3)

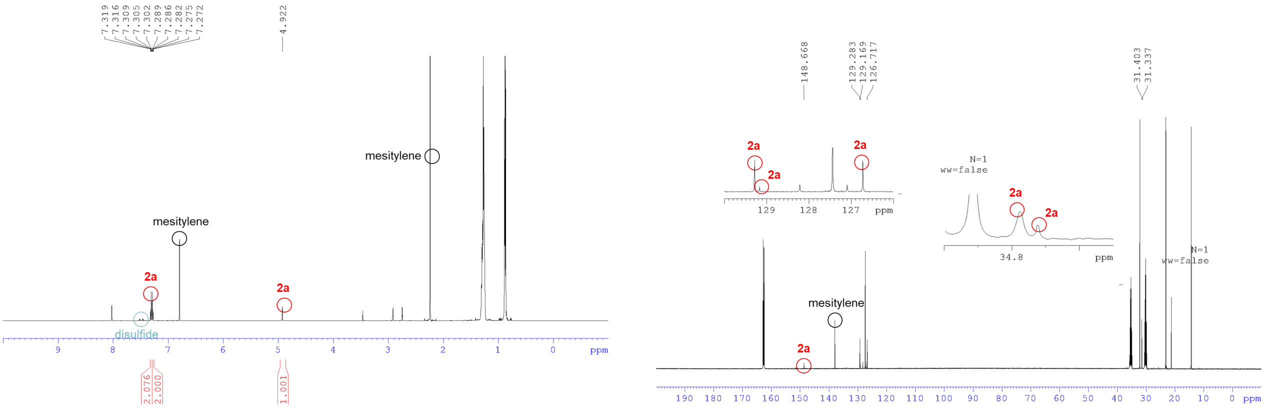

Supplementary Fig. S2 | ^{1}H NMR (600 MHz) and ^{13}C NMR (151 MHz) spectra of 4-*tert*-butylphenylthiol (**2b**, 0.019 mmol) in DMF-d_7 (0.5 mL) and hexane (25 µL) with mesitylene (5.0 µL, 0.036 mmol) as an internal standard.

Chemical shifts of 4-*tert*-butylphenylthiol are listed. ^{1}H NMR δ 4.92 (s, 1H, SH), 7.28 (AA'BB', 2H, aromatic), 7.31 (AA'BB', 2H, aromatic). The signal of the *t*Bu group was overlapped with the methylene signals of hexane. ^{13}C NMR δ 31.3, 31.4, 126.7, 129.2, 129.3, 148.7.

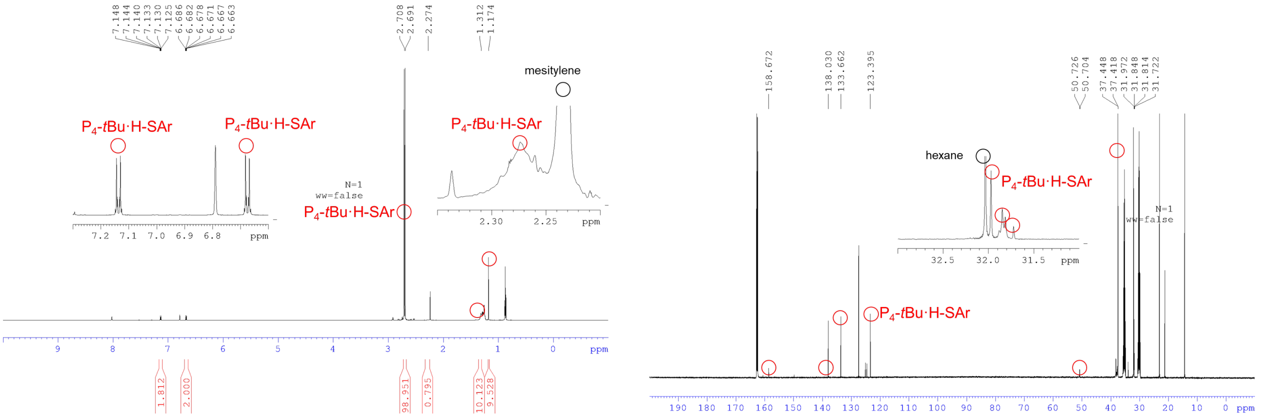

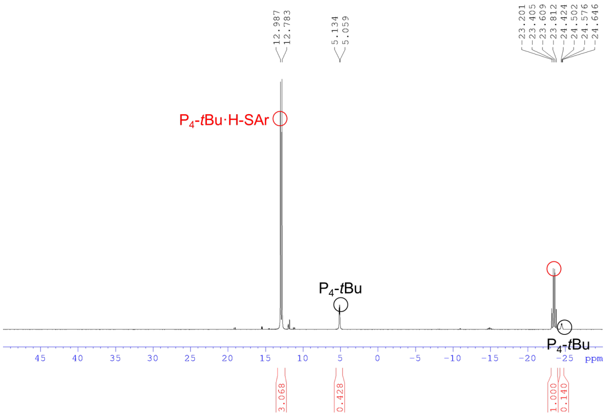

Supplementary Fig. S3 | ^{1}H NMR (600 MHz), ^{13}C NMR (151 MHz), and ^{31}P NMR (243 MHz) spectra of 4-*tert*-butylphenylthiol (**2b**, 0.019 mmol) and P_4-*t*Bu (0.020 mmol in 25 µL hexane) in DMF-d_7 (0.5 mL) with mesitylene (5.0 µL, 0.036 mmol) as an internal standard.

Chemical shifts of generated $[P_4\text{-}t\text{Bu-H}]^+\cdot[S(C_6H_4\text{-}t\text{Bu})]^-$ phosphazenium salt are listed. ^{1}H NMR δ 1.17 (s, 9H, *t*Bu), 1.31 (br, 9H, *t*Bu), 2.27 (br, 1H, NH), 2.70 (d, J = 9.9 Hz, 54H, NMe_2), 6.68 (AA'BB', 2H, aromatic), 7.14 (AA'BB', 2H, aromatic). ^{13}C NMR δ 31.7, 31.8 (d, J = 5.1 Hz), 32.0, 37.4 (d, J = 4.5 Hz), 50.7 (d, J = 3.4 Hz), 123.4, 133.7, 138.0, 158.7. ^{31}P NMR δ 12.7 (d, J = 49.5 Hz, 3P), -23.5 (q, J = 49.5 Hz, 1P).

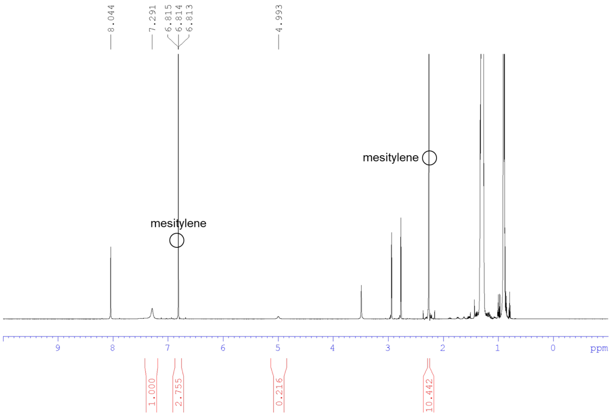

Supplementary Fig. S4 | ^{1}H NMR (600 MHz) spectrum of 4-*tert*-butylphenylthiol (**2b**, 0.02 mmol) and K_3PO_4 (0.020 mmol) in DMF-d_7 (0.5 mL) and hexane (25 µL) with mesitylene (5.0 µL, 0.036 mmol) as an internal standard.

Broad signals from 7.21ppm to 7.59ppm on the aryl region and 4.99ppm on the SH region were observed, which are clearly difference from the ^{1}H NMR analysis in Supplementary Fig. S1, S2, S4.

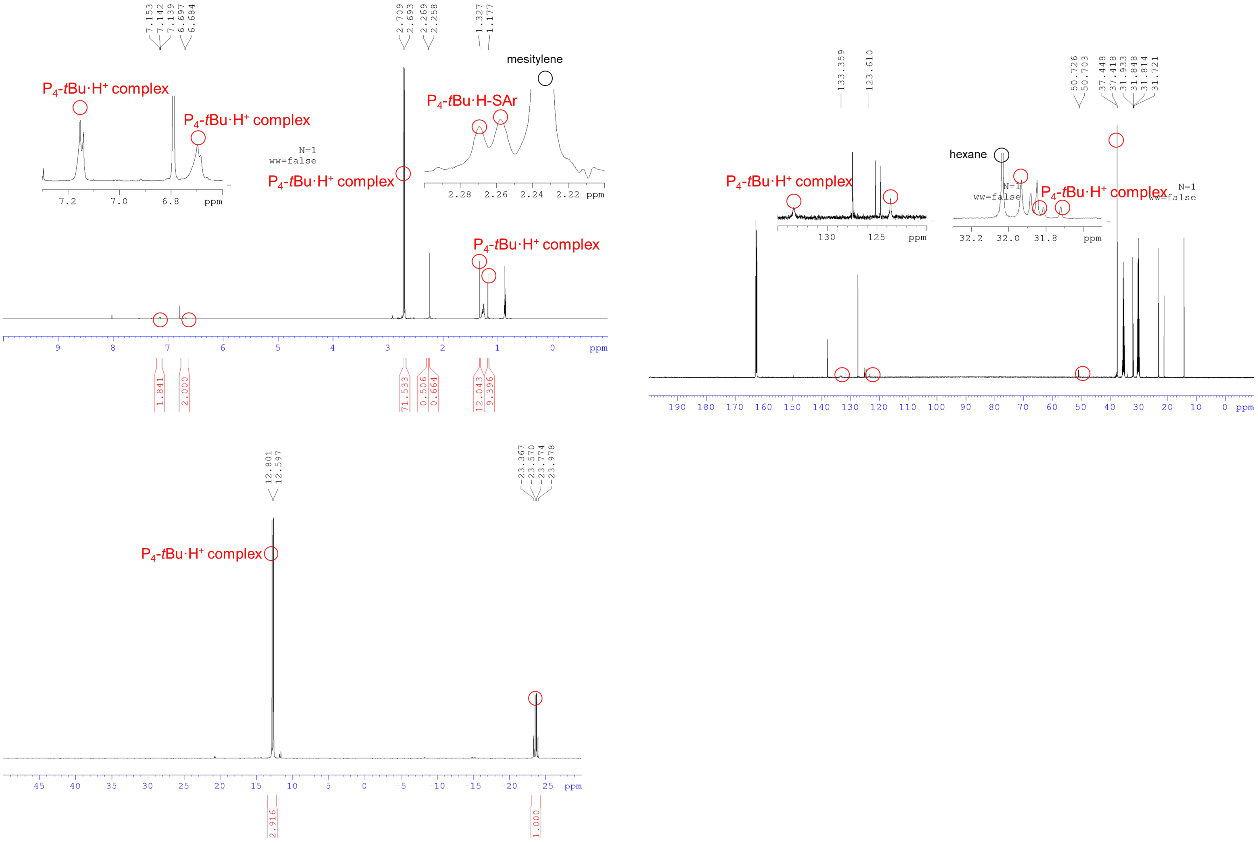

Supplementary Fig. S5 | ^{1}H NMR (600 MHz), ^{13}C NMR (151 MHz), and ^{31}P NMR (243 MHz) spectra of 4-*tert*-butylphenylthiol (**2b**, 0.019 mmol), P_4-*t*Bu (0.020 mmol in 25 μL hexane), and K_3PO_4 (0.020 mmol) in DMF-d_7 (0.5 mL) with mesitylene (5.0 μL, 0.036 mmol) as an internal standard.

Chemical shifts of the product are listed. ^{1}H NMR δ 1.18 (s, 9H, *t*Bu), 1.32 (br, 9H, *t*Bu), 2.26-2.28 (br-d, 1H, NH), 2.70 (d, J = 10.0 Hz, 54H, NMe_2), 6.69 (br-d, 2H, aromatic), 7.14 (br-d, 2H, aromatic). ^{13}C NMR δ 31.7, 31.8 (d, J = 5.1 Hz), 31.9, 37.4 (d, J = 4.5 Hz), 50.7 (d, J = 3.4 Hz), 123.6 (br), 133.4 (br). Two signals of aryl carbons were too small to be assigned. ^{31}P NMR δ 12.7 (d, J = 49.5 Hz, 3P), -23.6 (q, J = 49.5 Hz, 1P).

9-4. NBO charges of phenylthiolate and phenylthiolate-K_3PO_4 complex by DFT calculation

We investigated NBO charges of phenylthiolate, K_3PO_4, and phenylthiolate-K_3PO_4 complex by DFT calculations. All DFT calculations were performed in Gaussian 09.[S8] All structures were optimized in and characterized by a opt+freq calculation at the B3LYP/6-31G(d) level of theory.

Calculated NBO charges of phenylthiolate

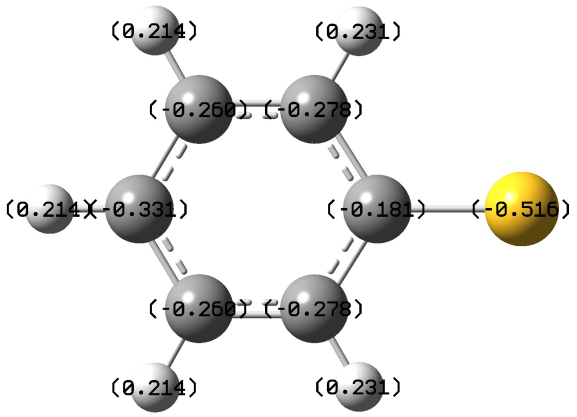

Shown numbers are NBO charges.

Calculated NBO charges of K_3PO_4

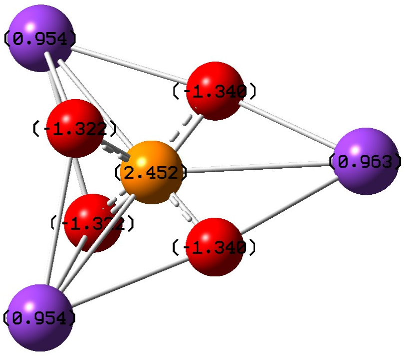

Shown numbers are NBO charges.

Calculated NBO charges of phenylthiolate-K_3PO_4 complex

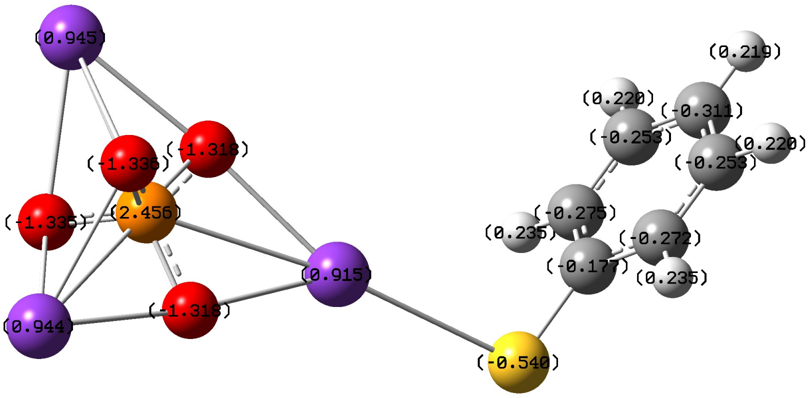

Shown numbers are NBO charges.

9-5. Effect of TEMPO toward the catalytic decomposition

The presence of a catalytic or stoichiometric amount of TEMPO interfered with the decomposition of PEEK (**1**) with 2-ethylhexanethiol (**2a**) by the base catalysts (Supplementary Table S7). In these cases, di(2-ethyl-1-hexyl)disulfide was generated. Next, we checked the conversion of **2a** into the disulfide mediated by TEMPO in the absence of PEEK. As a result, even in the presence or absence of the base catalysts, **2a** was consumed and the disulfide was generated in about a half yield (Supplementary Table S8). This means that TEMPO was not suitable radical scavenger for the catalytic decomposition.

Supplementary Table S7 | Inhibition of the decomposition by TEMPO

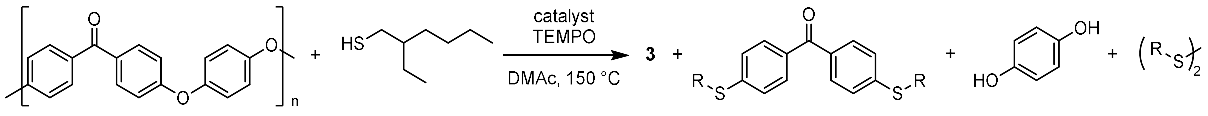

1 (powder) **2a** (2.5 equiv.) **4a** (R = 2-Ethyl-Hexyl) **5**

Entry	catalyst	TEMPO	Time	**3** (%)	**4a** (%)	**5** (%)	$(RS)_2$ (%)
1	P_4-*t*Bu (10 mol%) K_3PO_4 (5 mol%)	0.2 equiv.	17 h	26	50	49	36
2	P_4-*t*Bu (10 mol%) K_3PO_4 (5 mol%)	2.5 equiv.	16 h	2	0.2	ND	44
3[b]	P_4-*t*Bu (10 mol%) K_3PO_4 (5 mol%)	2.5 equiv.	22 h	ND	ND	ND	56
4	P_4-*t*Bu (10 mol%)	2.5 equiv.	16 h	ND	ND	ND	44
5	K_3PO_4 (10 mol%)	2.5 equiv.	21 h	ND	ND	ND	44

[a] A mixture of **1** (powder, 0.2 mmol relative to the molecular weight of the monomer), thiol (0.5 mmol), catalyst, TEMPO, and DMAc (*N*,*N*-dimethylacetamide, 0.4 mL) was stirred at 150 ºC. Yields were determined by ^{1}H NMR. [b] DMI (0.4 mL) were used instead of DMAc.

Supplementary Table S8 | Conversion of thiols into disulfides by TEMPO

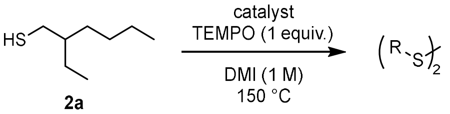

2a

Entry	catalyst	Time	Conv. Of **2a** (%)	$(RS)_2$ (%)
1	-	2 h	90	50
2	P_4-*t*Bu (10 mol%) K_3PO_4 (5 mol%)	22 h	79	48

[a] A mixture of **2a** (0.2 mmol), TEMPO (0.2 mmol), catalyst, and DMAc (*N*,*N*-dimethylacetamide, 0.2 mL) was stirred at 150 ºC. Yields were determined by ^{1}H NMR.

9-6. Gel Permeation Chromatography (GPC) analysis of resins

These PSU (**7** and **7'**), PEES (**10**), PPSU, (**11** and babe bottle **11'**) and PESU (**13**) were analyzed by high temperature GPC analysis in the previous study (Supplementary Table S9).[S9] In the case of PESU (**13**), it is assumed that soluble low-weight oligomers were only detected. PEI (**16**) pellets was newly analyzed using a Tosoh HLC-8321GPC/HT with TSKgel GMH_{HR}-H (S) HT2 column at 220 °C. 1-Chloronaphthalene was used as the eluent and number-average molecular weight (M_n), weight-average molecular weight (M_w), and dispersity ($Đ$) were calculated using refractive index (RI) chromatograms against TSK gel polystyrene standards. The results were shown as follows. Of note, PEEK (**1**, catalog spec: average $M_w \sim 20800$ and average $M_n \sim 10300$) is insoluble in organic solvents and could not be analyzed at all with 1-chloronaphthalene at 220°C.

Supplementary Table S9 | High-temperature GPC analysis of resins

Resin	M_n	M_w	M_z	M_v	M_p	$Đ$
PSU (**7**) [S9]	25126	50075	80605	50075	44428	1.99
PSU (**7**) (measured at 100 °C) [S9]	17467	37148	62832	37148	32735	2.13
PSU (**7'**) [S9]	29761	58859	96857	58859	50684	1.98
PEES (**10**) [S9]	21458	45338	72093	45338	42578	2.11
PPSU (**11**) [S9]	16501	41812	73426	41812	37898	2.53
baby bottle made up of PPSU (**11'**) [S9]	17932	43133	71962	43133	41361	2.41
PESU (**13**) [S9]	2123	4120	5610	4120	5072	1.94
PEI (**16**)	20316	48922	87027	48922	42651	2.41

Supplementary references

S1) Minami, Y., Matsuyama, N., Takeichi, Y., Watanabe, R., Mathew, S. & Nakajima, Y. Depolymerization of robust polyetheretherketone to regenerate monomer units using sulfur reagents. *Commun. Chem.* **6**, 14 (2023).

S2) Sugahara, T., Murakami, K., Yorimitsu, H. & Osuka, A. Palladium-Catalyzed Amination of Aryl Sulfides with Anilines. *Angew. Chem. Int. Ed.* **53**, 9329–9333 (2014).

S3) Takenaga, N., Yoto, Y., Hayashi, T., Miyamoto, N., Nojiri, H., Kumar, R. & Dohi, T. Catalytic and non-catalytic selective aryl transfer from (mesityl)iodonium(III) salts to diarylsulfide compounds. *Arkivoc* 7–18 (2022).

S4) Mu, L., Fischer, C. R., Holland, J. P., Becaud, J., Schubiger, P. A., Schibli, R., Ametamey, S. M., Graham, K., Stellfeld, T., Dinkelborg, L. M. & Lehmann, L. ^{18}F-Radiolabeling of Aromatic Compounds Using Triarylsulfonium Salts. *Eur. J. Org. Chem.* 889–892 (2012).

S5) Lee, H. G., Milner, P. J. & Buchwald, S. L. *Org. Lett.* **15**, 5602–5605 (2013).

S6) Faye, A., Leduc, M. & Brisson, J. Crystallization control of etherethersulfone copolymers by regular insertion of an allyl functionality. *Polym. Chem.* **5**, 2548–2560 (2014).

S7) Jardel, D., Davies, C., Peruch, F., Massip, S. & Bibal, B. Protonated Phosphazenes: Structures and Hydrogen-Bonding Organocatalysts for Carbonyl Bond Activation. *Adv. Synth. Catal.* **358**, 1110−1118 (2016).

S8) Frisch, M. J., Trucks, G. W., Schlegel, H. B., Scuseria, G. E., Robb, M. A., Cheeseman, J. R., Scalmani, G., Barone, V., Mennucci, B., Petersson, G. A., Nakatsuji, H., Caricato, M., Li, X., Hratchian, H. P., Izmaylov, A. F., Bloino, J., Zheng, G., Sonnenberg, J. L., Hada, M., Ehara, M., Toyota, K., Fukuda, R., Hasegawa, J., Ishida, M., Nakajima, T., Honda, Y., Kitao, O., Nakai, H., Vreven, T., Montgomery, J. A., Jr., Peralta, J. E., Ogliaro, F., Bearpark, M., Heyd, J. J., Brothers, E., Kudin, K. N., Staroverov, V. N., Kobayashi, R., Normand, J., Raghavachari, K., Rendell, A., Burant, J. C., Iyengar, S. S., Tomasi, J., Cossi, M., Rega, N., Millam, J. M., Klene, M., Knox, J. E., Cross, J. B., Bakken, V., Adamo, C., Jaramillo, J., Gomperts, R., Stratmann, R. E., Yazyev, O., Austin, A. J., Cammi, R., Pomelli, C., Ochterski, J. W., Martin, R. L., Morokuma, K., Zakrzewski, V. G., Voth, G. A., Salvador, P., Dannenberg, J. J., Dapprich, S., Daniels, A. D., Farkas, Ö., Foresman, J. B., Ortiz, J. V., Cioslowski, J. & Fox, D. J. *Gaussian 09* (Gaussian, Inc., Wallingford CT, 2009).

S9) Minami, Y., Inagaki, Y., Tsuyuki, T., Sato, K. & Nakajima, Y. Hydroxylation-depolymerization of oxyphenylene-based super engineering plastics to regenerate arenols. *JACS Au* **3**, 2323–2332 (2023).
