# Supplementary material for: Catalytic thiolation-depolymerization-like decomposition of oxyphenylene-type super engineering plastics via selective carbon–oxygen main chain cleavages
Source: Commun Chem. 2024 Feb 20;7:37. doi: 10.1038/s42004-024-01120-7 (PMC10879179; doi:10.1038/s42004-024-01120-7)
Supplement: Supplementary file 3 — Description of Additional Supplementary File [file 42004_2024_1120_MOESM3_ESM.pdf]

Description of Additional Supplementary Files

File Name: Supplementary Data 1
Description: original ^{1}H and ^{13}C NMR spectra

File Name: Supplementary Data 2
Description: computed energy values and optimized molecular geometries
