# Supplementary material for: Catalytic thiolation-depolymerization-like decomposition of oxyphenylene-type super engineering plastics via selective carbon–oxygen main chain cleavages
Source: Commun Chem. 2024 Feb 20;7:37. doi: 10.1038/s42004-024-01120-7 (PMC10879179; doi:10.1038/s42004-024-01120-7)

Supplementary Data 1

NMR charts of new chemicals.

^{1}H NMR (600 MHz) and ^{13}C NMR (151 MHz) spectra of **4a** ($CDCl_3$)

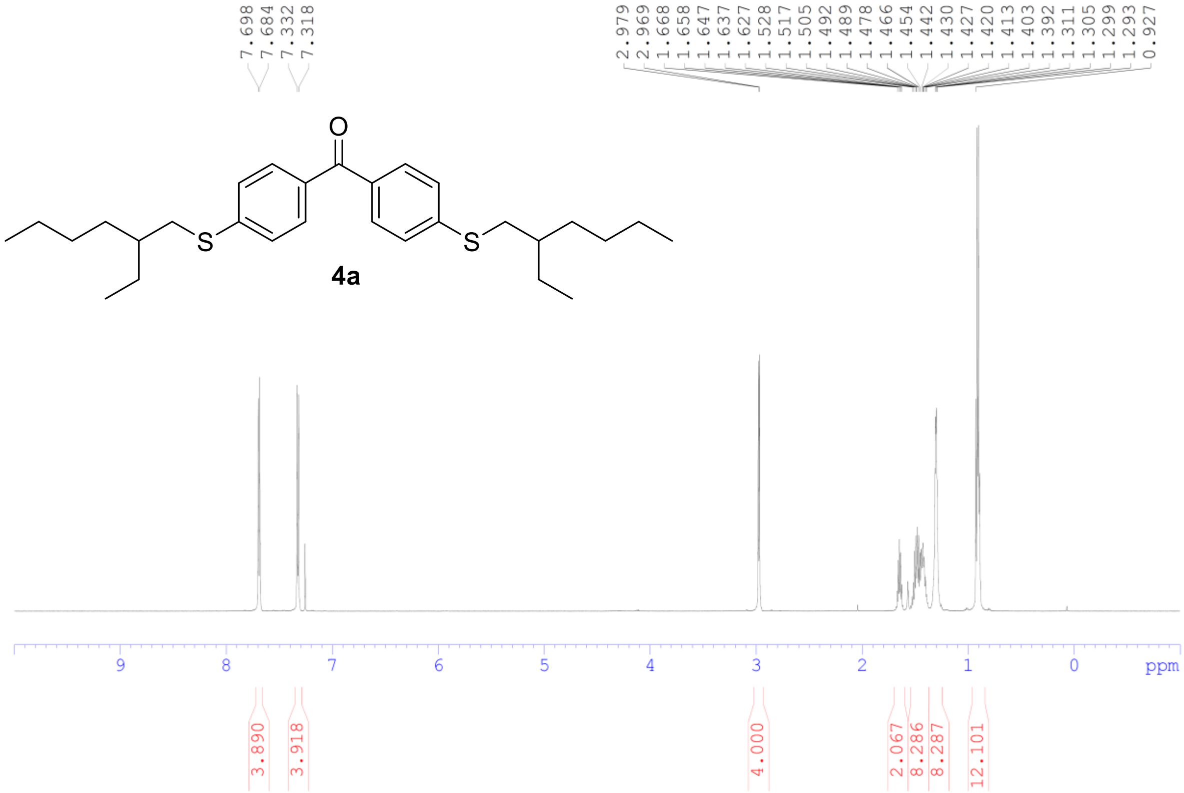

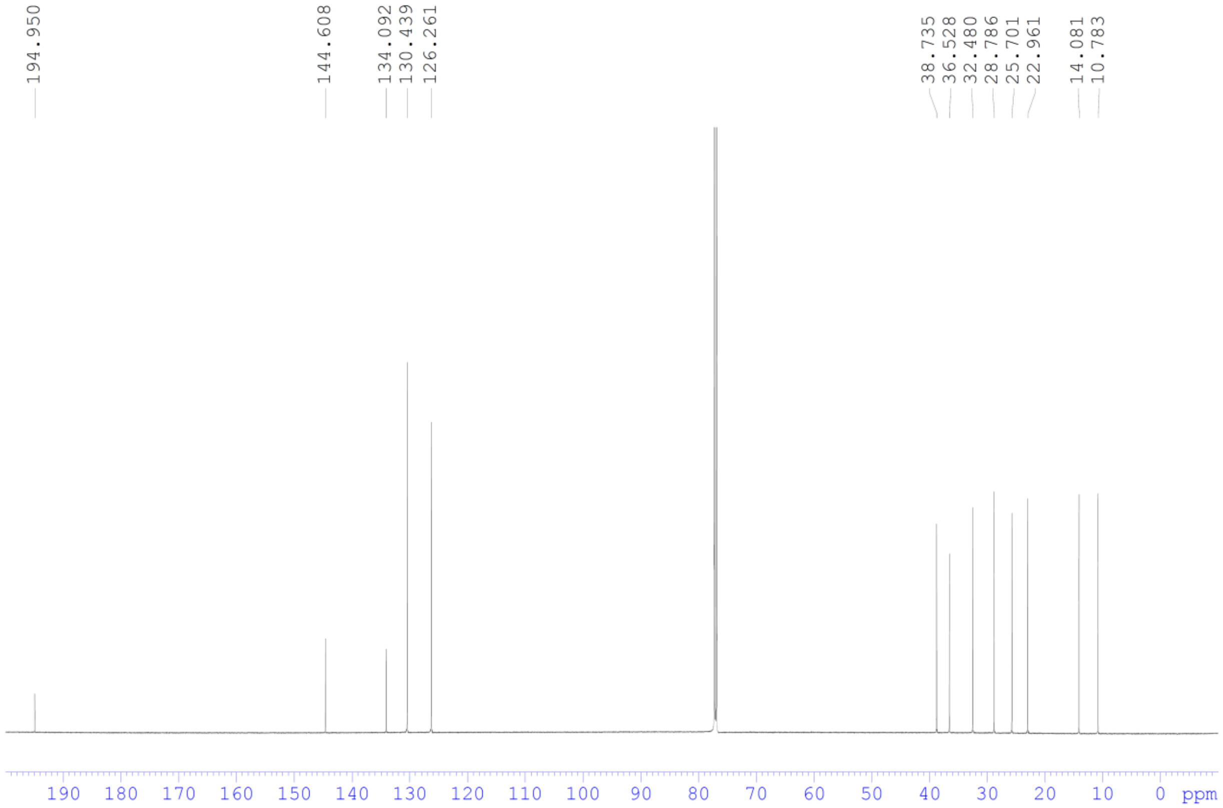

^{1}H NMR (600 MHz) and ^{13}C NMR (151 MHz) spectra of **8a** ($CDCl_3$)

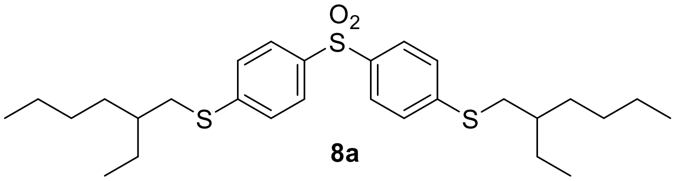

8a

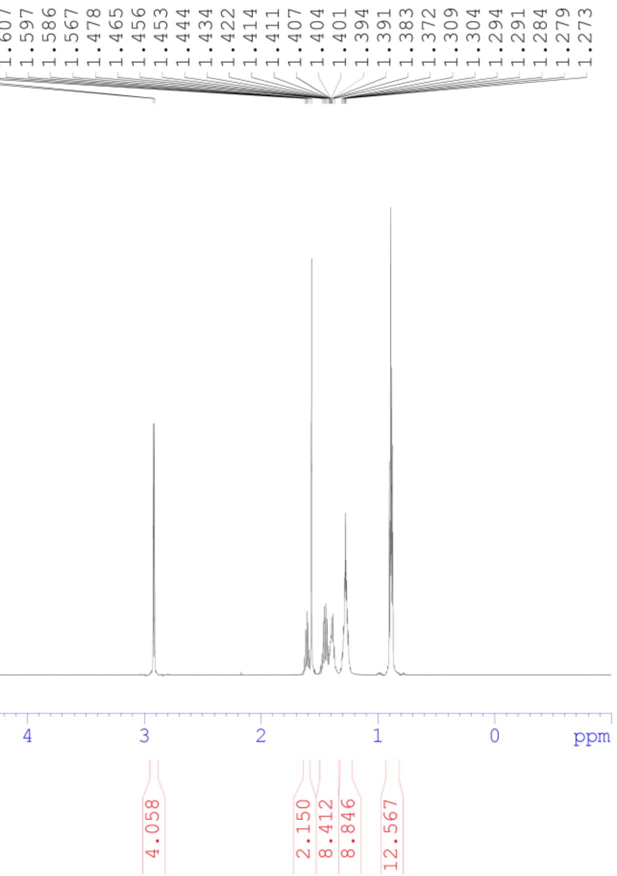

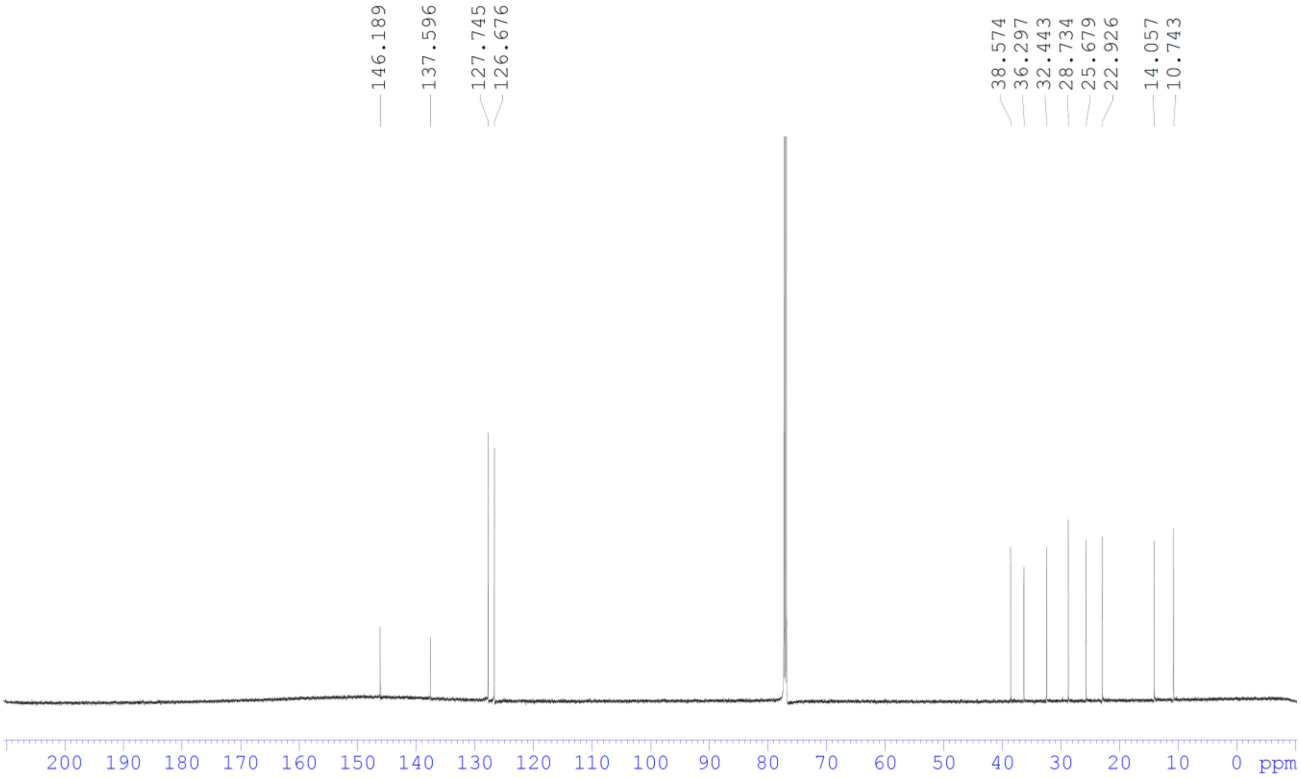

¹H NMR (600 MHz) and ¹³C NMR (151 MHz) spectra of **14** ($CDCl_3$)

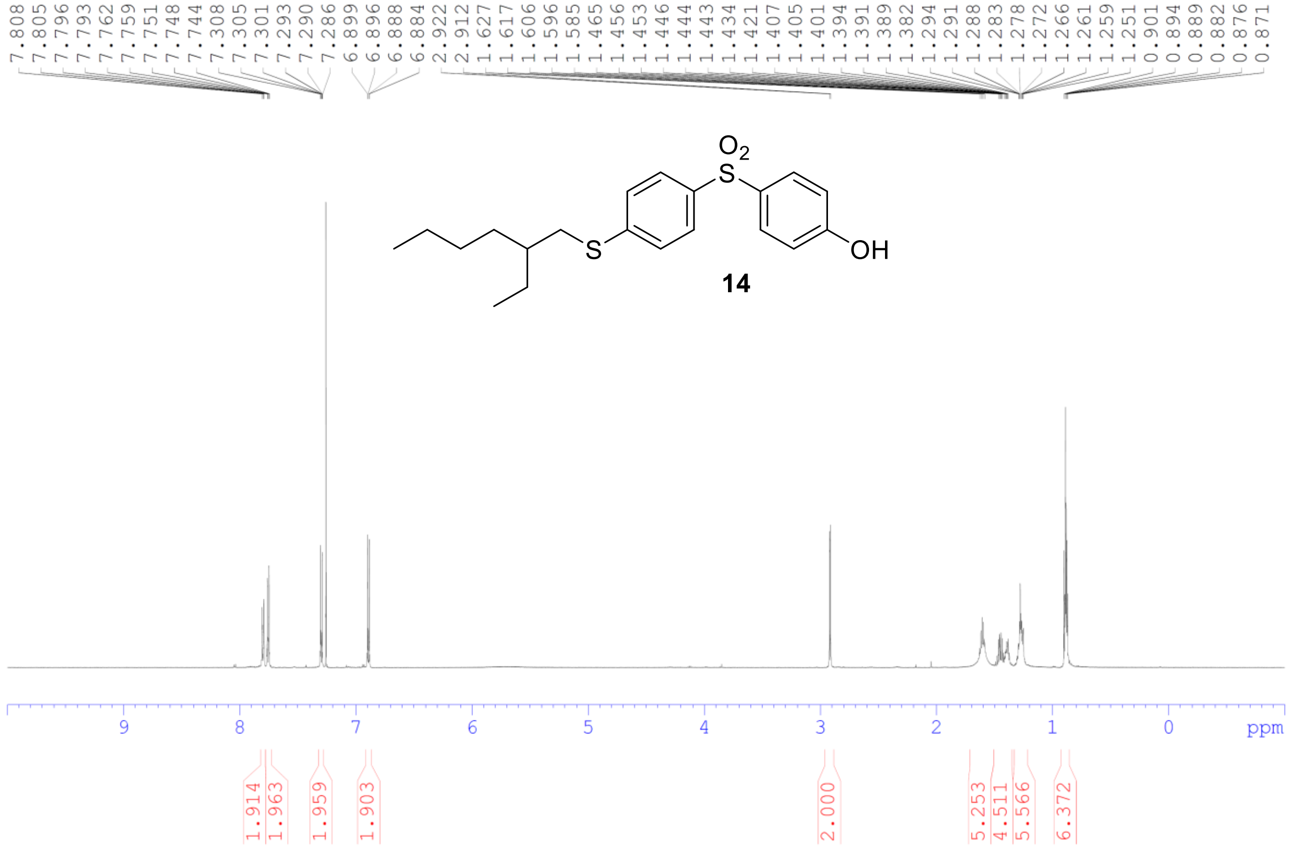

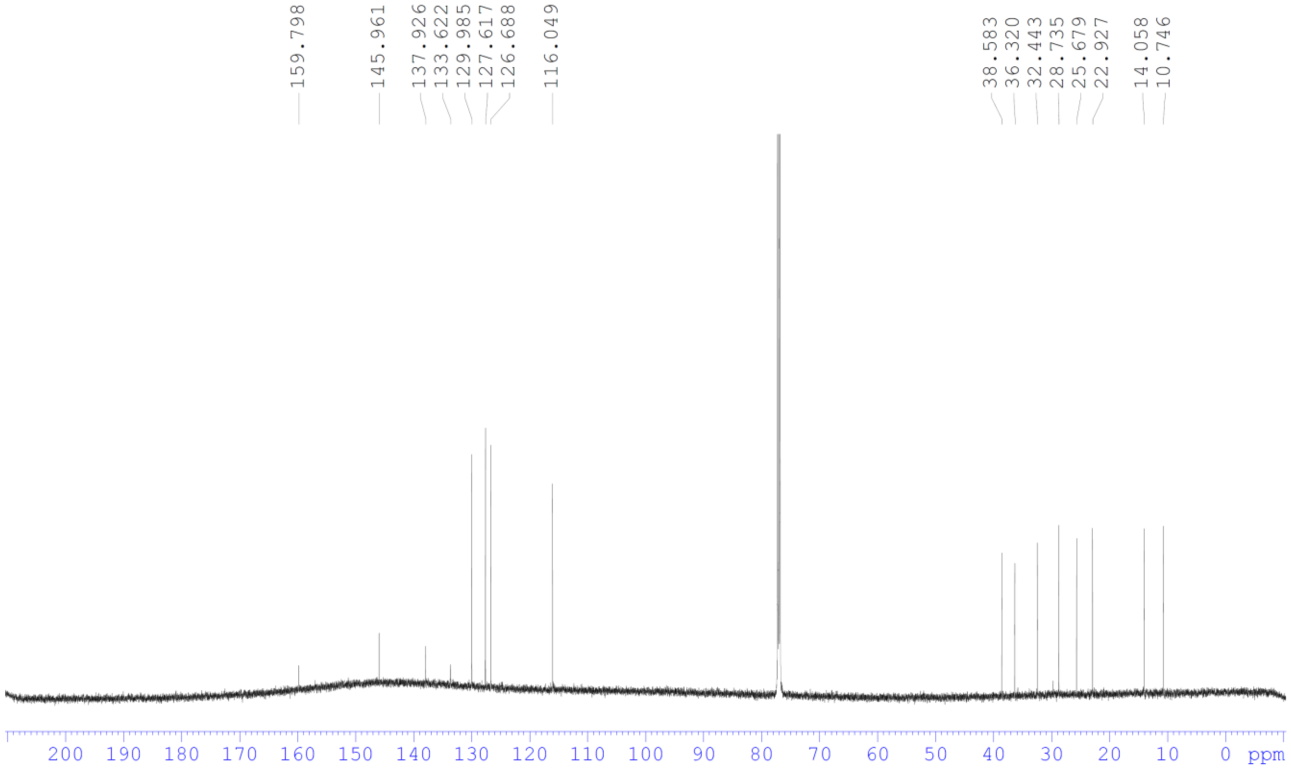

^{1}H NMR (600 MHz) and ^{13}C NMR (151 MHz) spectra of **17** ($CDCl_3$)

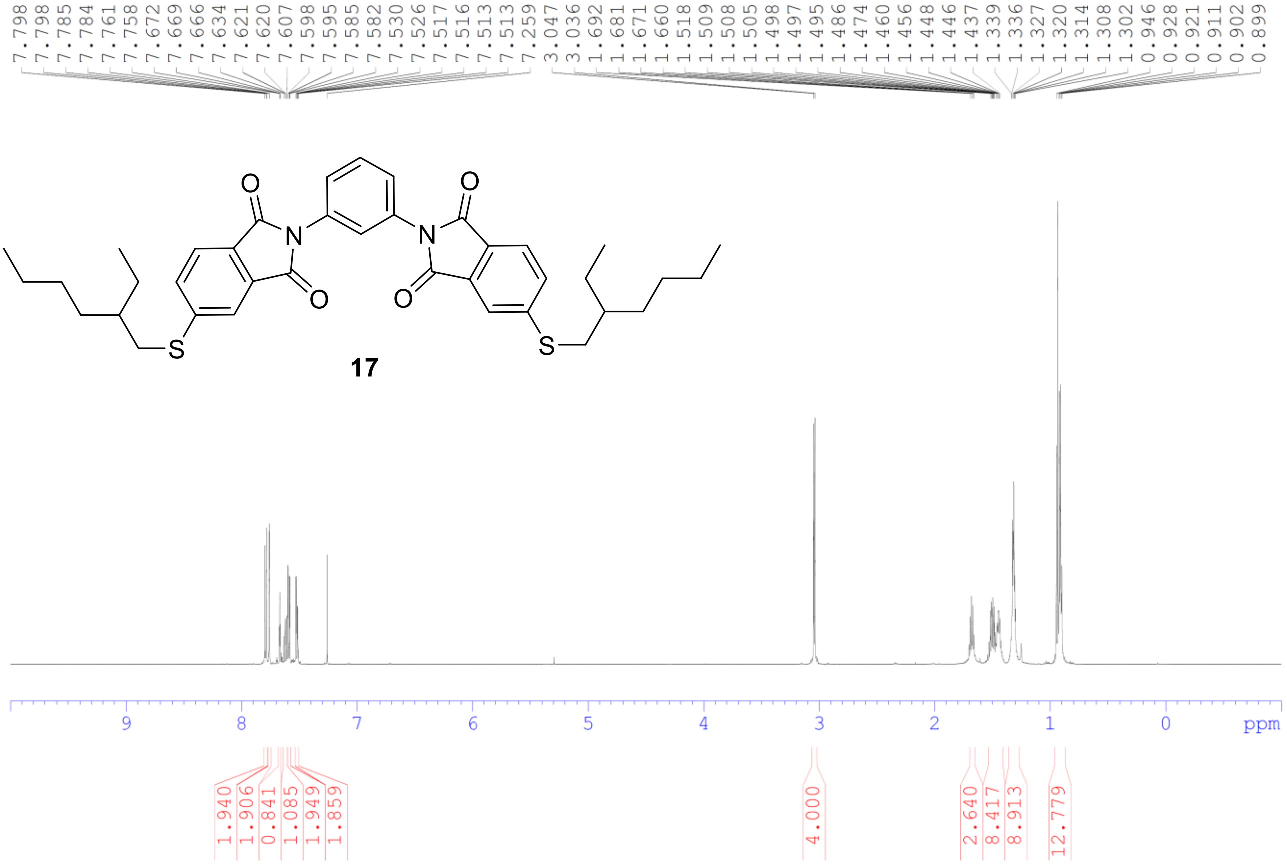

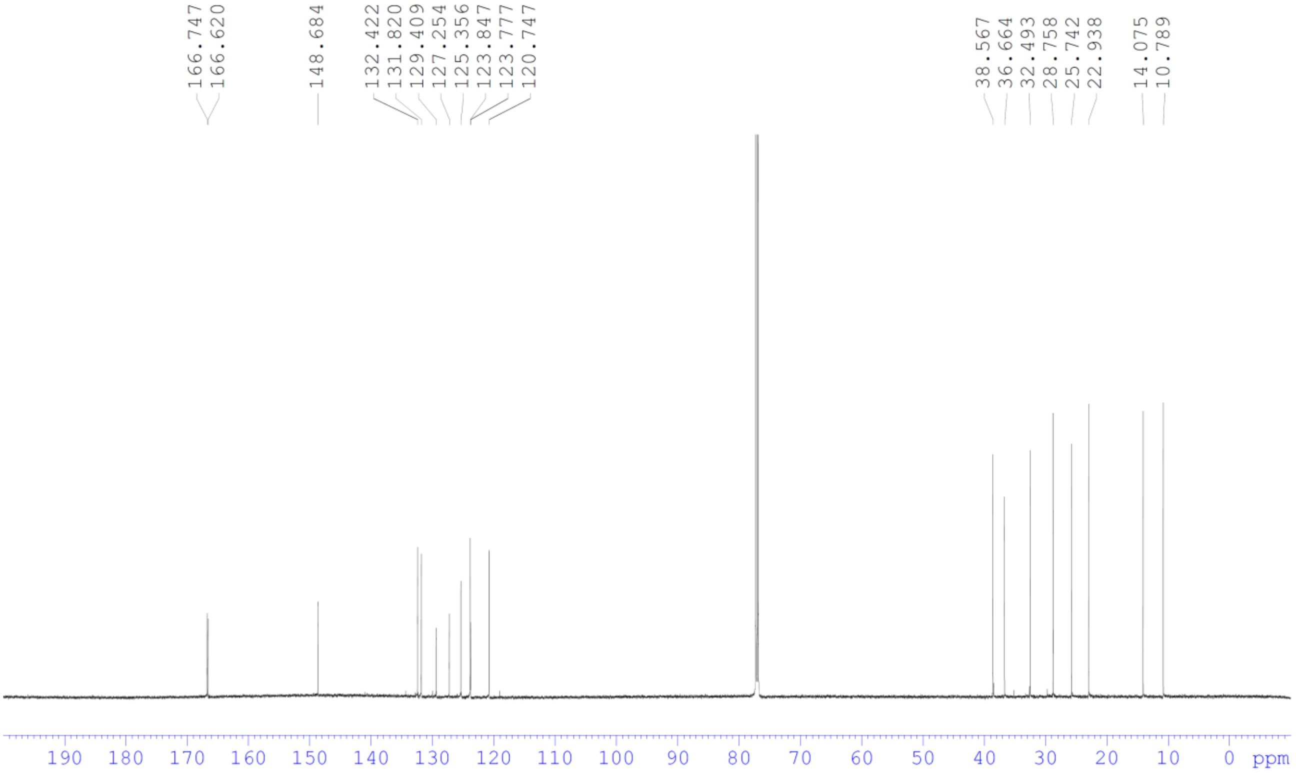

^{1}H NMR (600 MHz) and ^{13}C NMR (151 MHz) spectra of **8b** ($CDCl_3$)

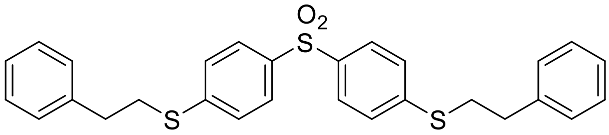

8b

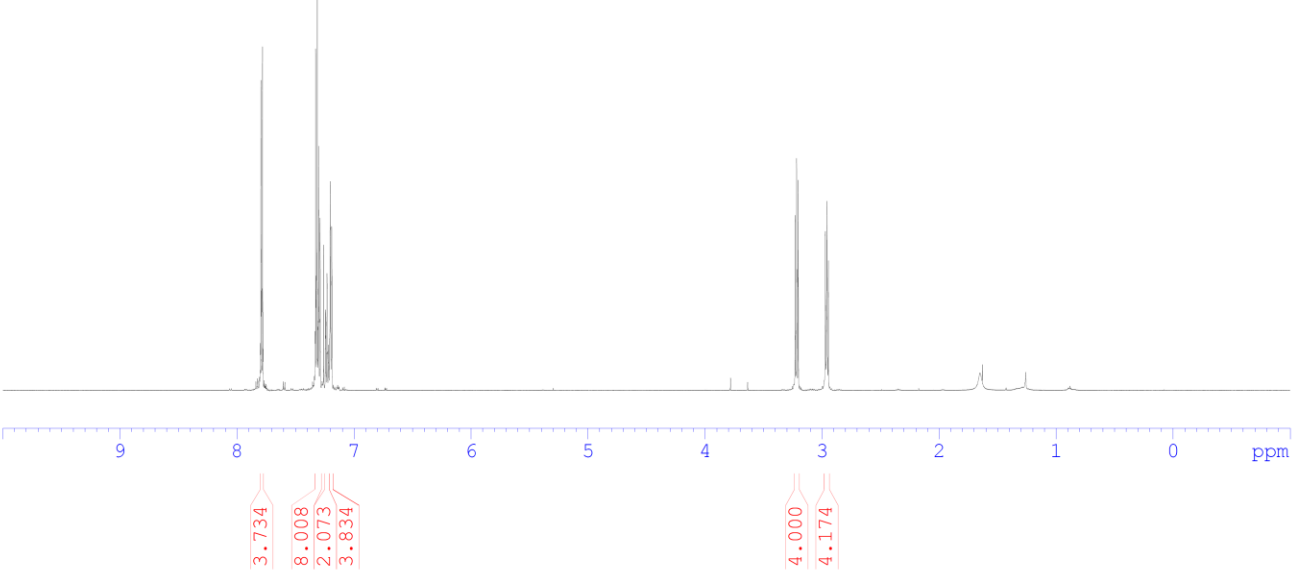

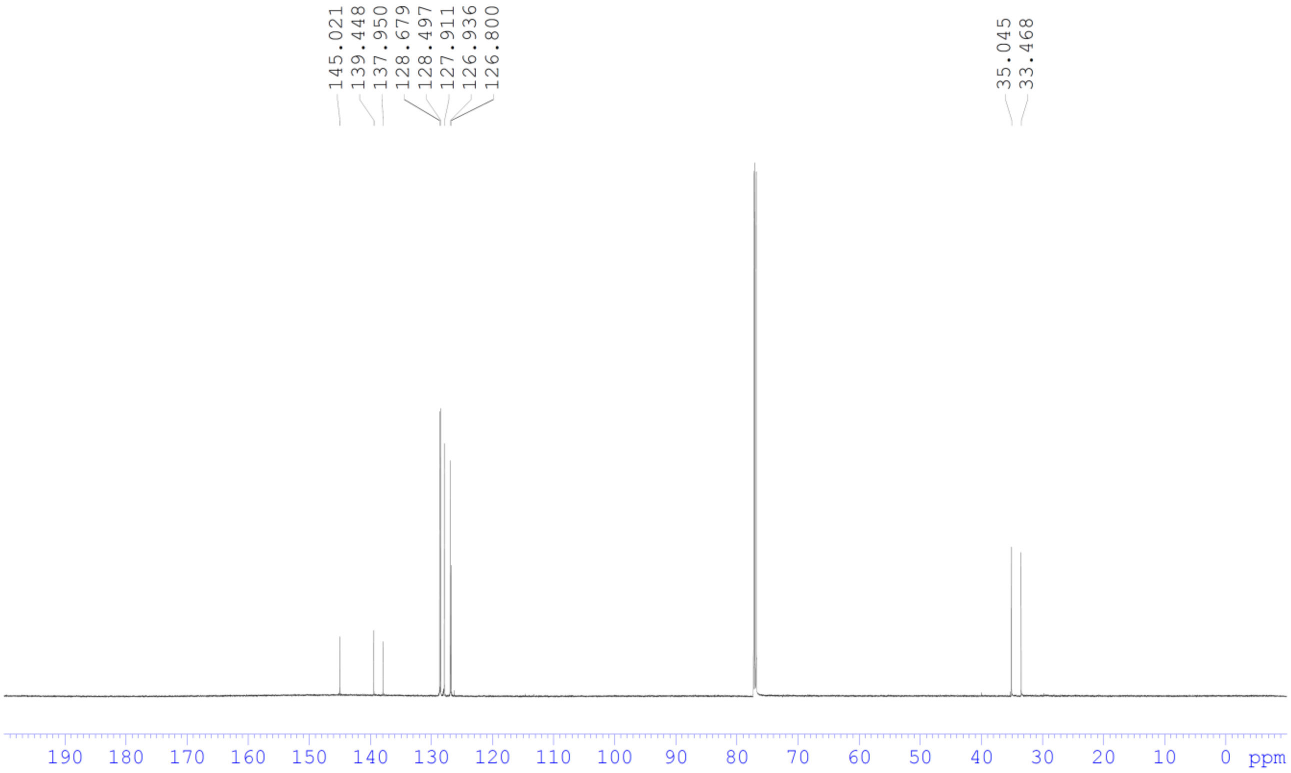

^{1}H NMR (600 MHz) and ^{13}C NMR (151 MHz) spectra of **8c** (acetone-d_6)

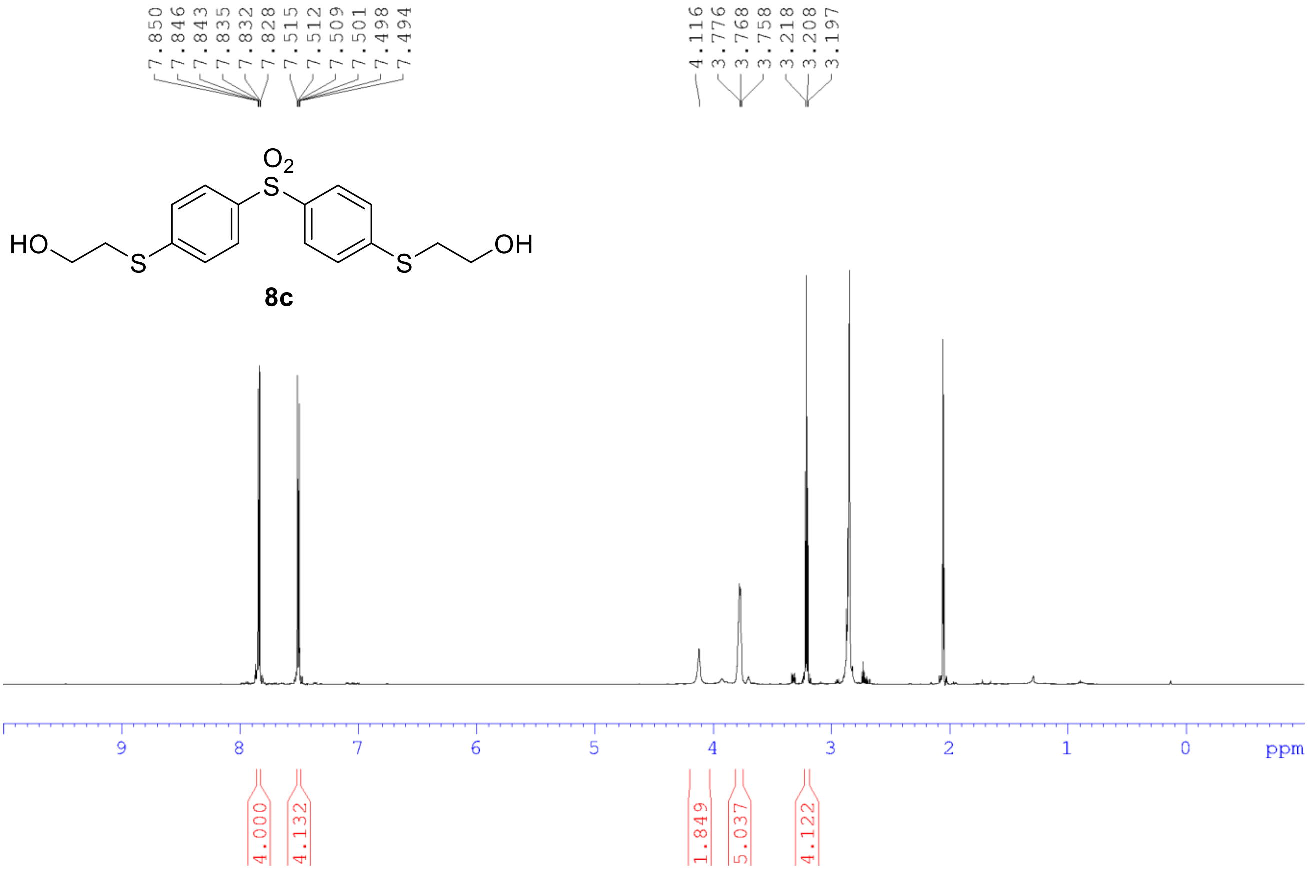

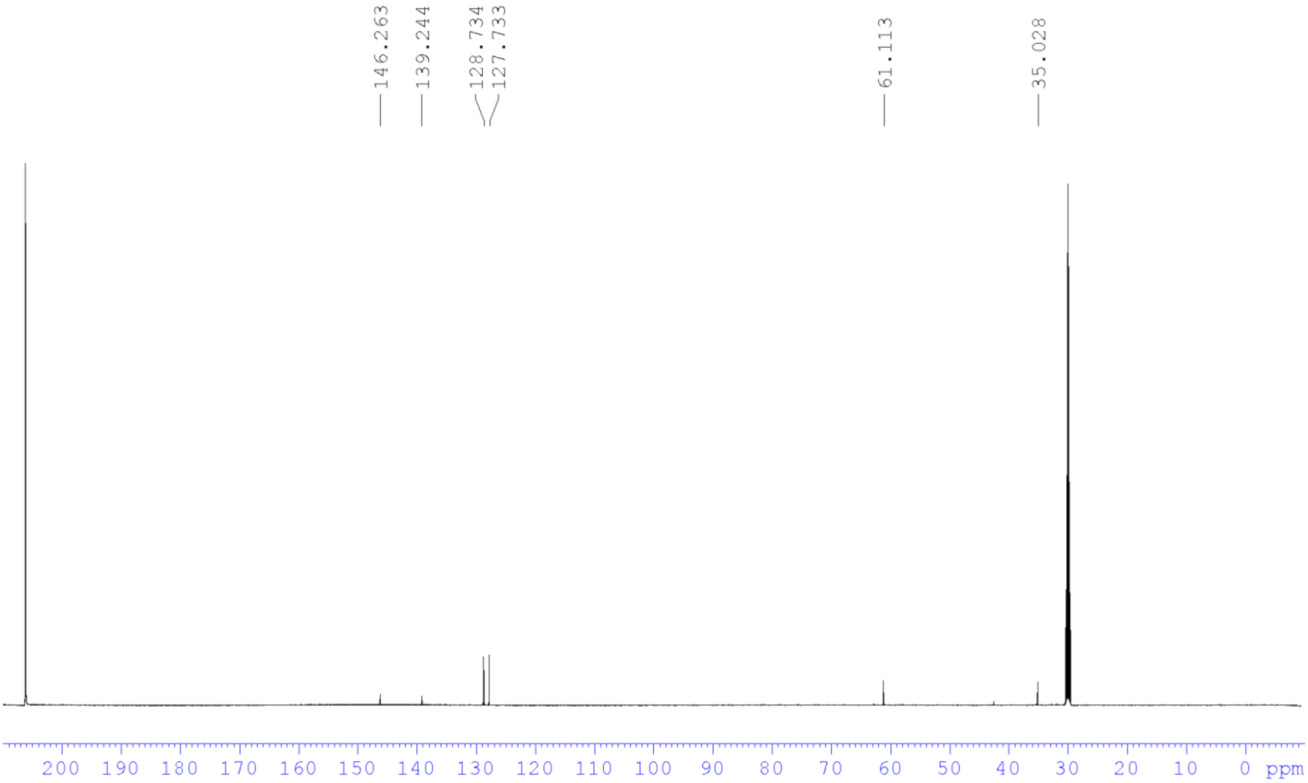

^1H NMR (600 MHz) and ^{13}C NMR (151 MHz) spectra of **8d** ($CDCl_3$)

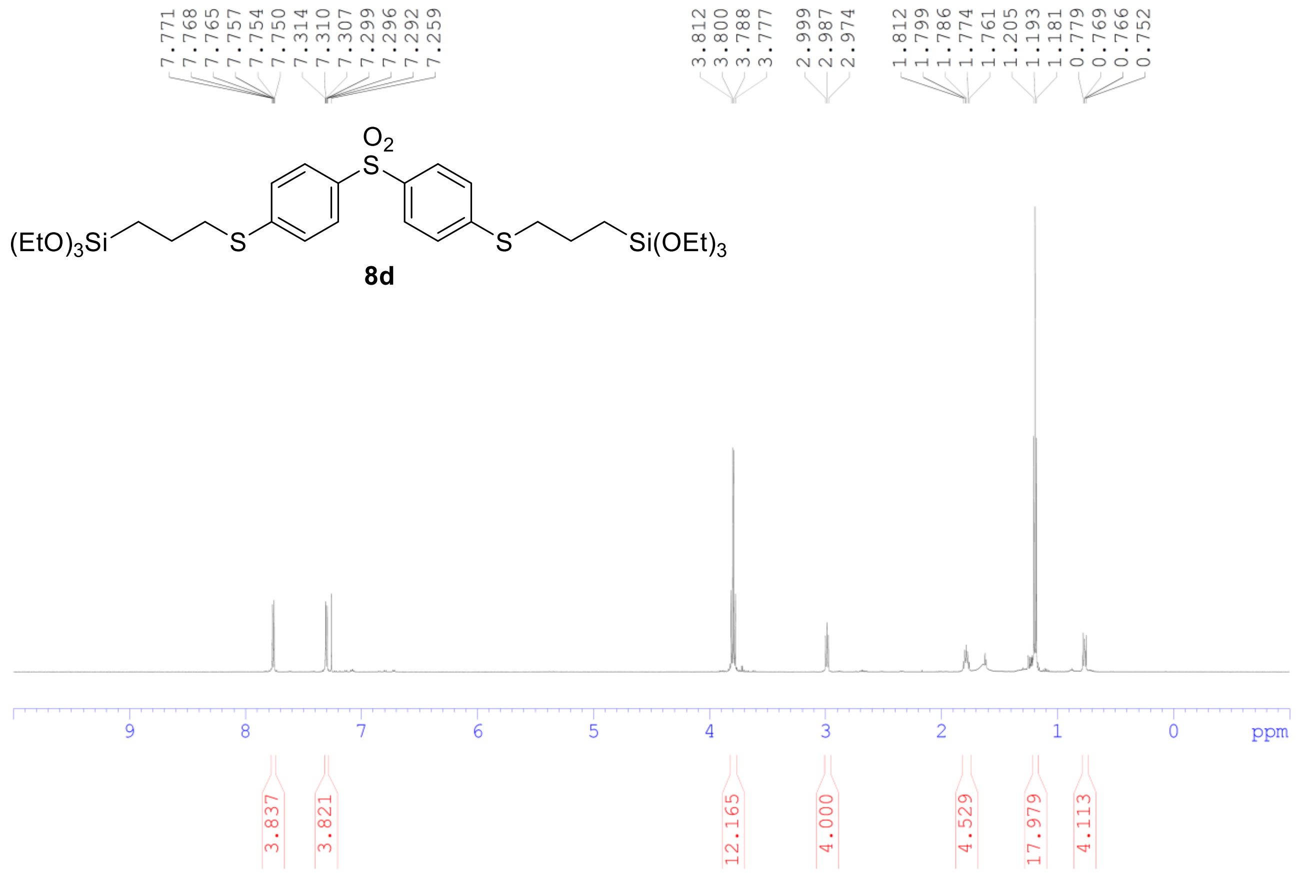

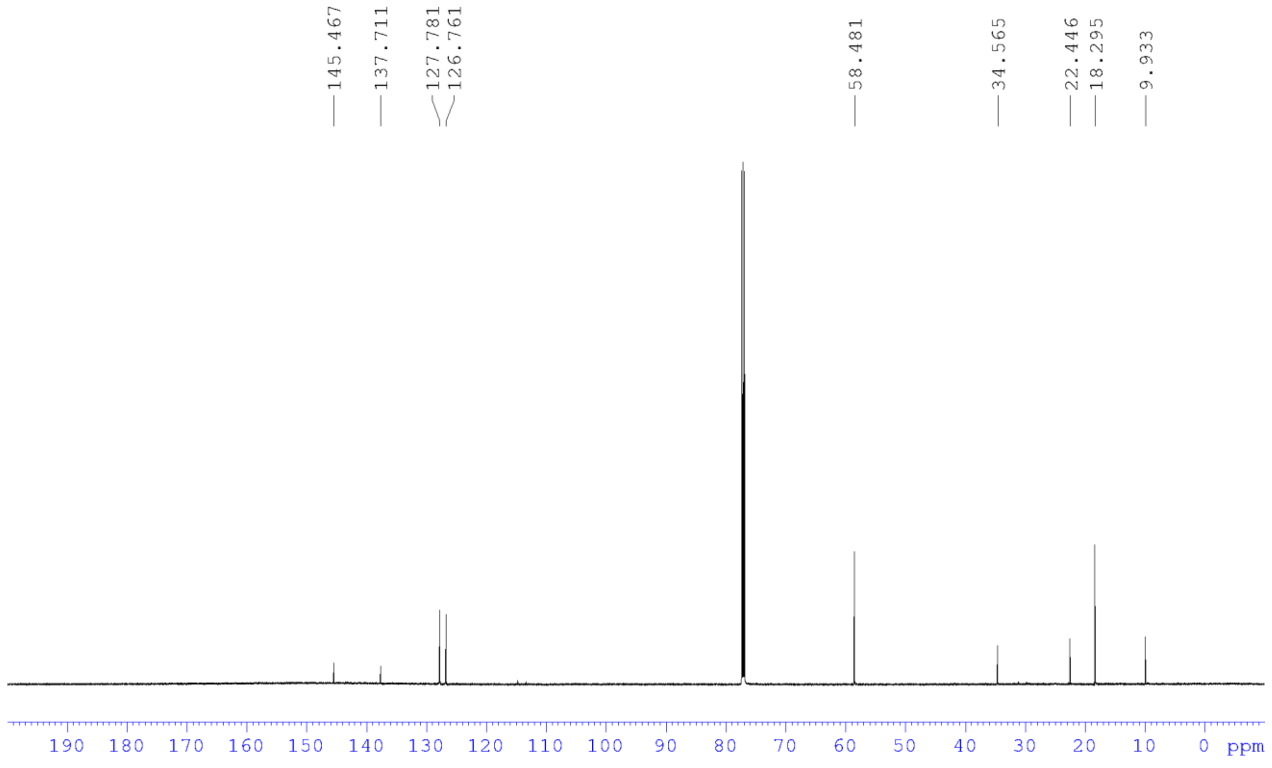

^{1}H NMR (600 MHz) and ^{13}C NMR (151 MHz) spectra of **8e** ($CDCl_3$)

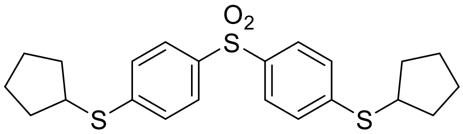

8e

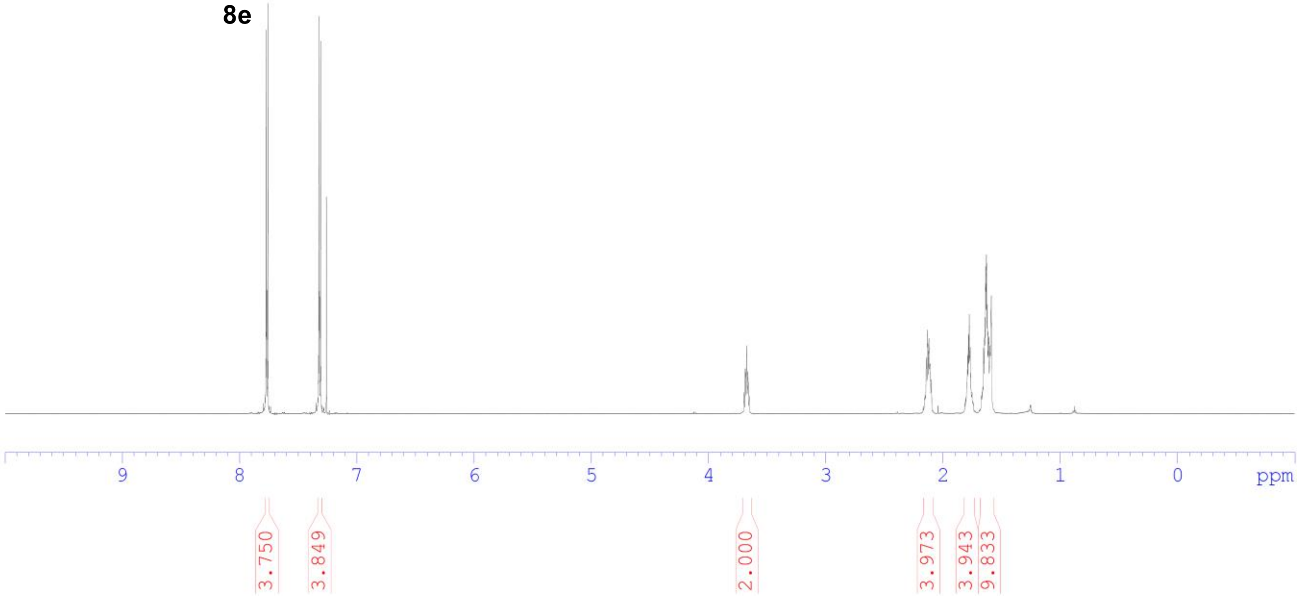

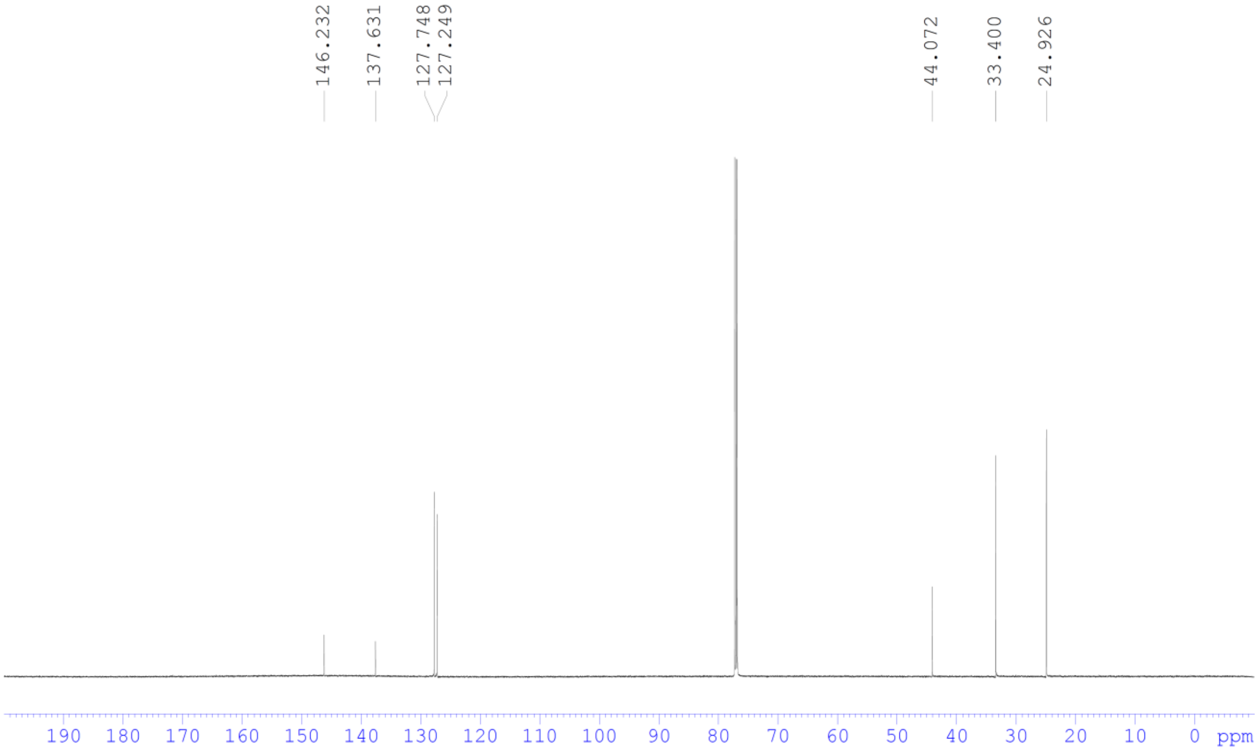

^{1}H NMR (600 MHz) and ^{13}C NMR (151 MHz) spectra of **8f** ($CDCl_3$)

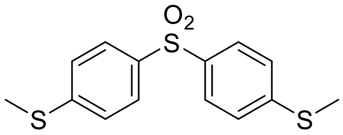

8f

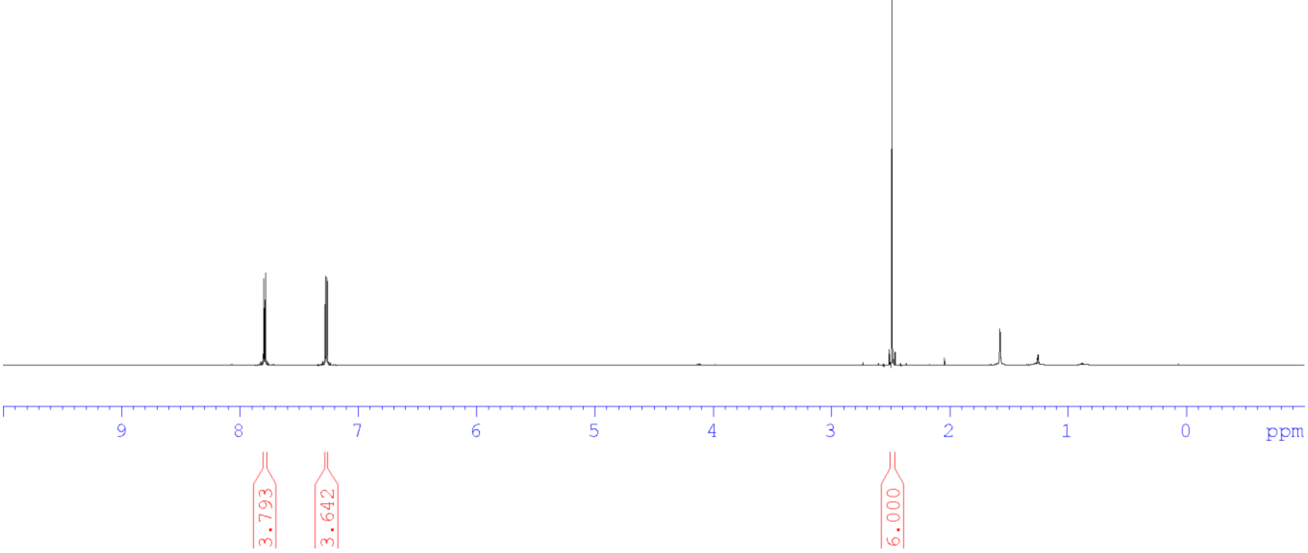

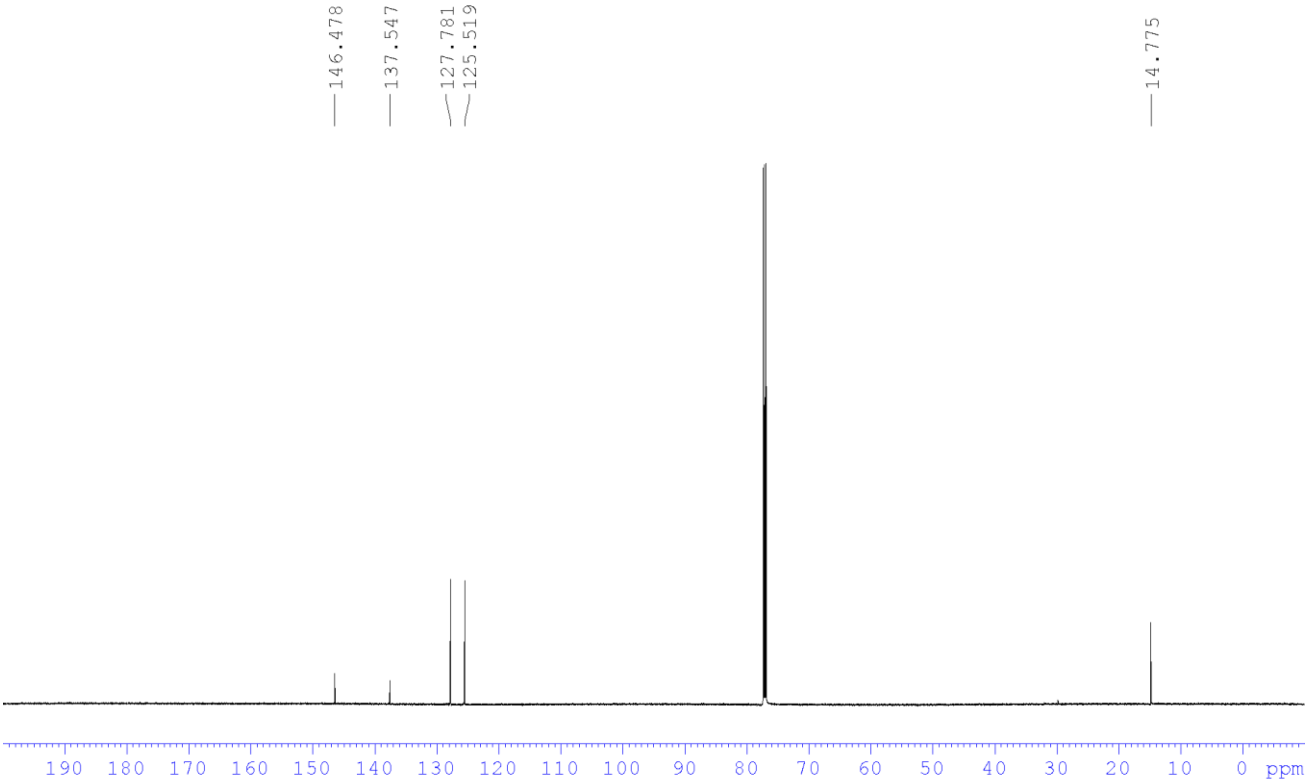

^{1}H NMR (600 MHz) and ^{13}C NMR (151 MHz) spectra of **8g** ($CDCl_3$)

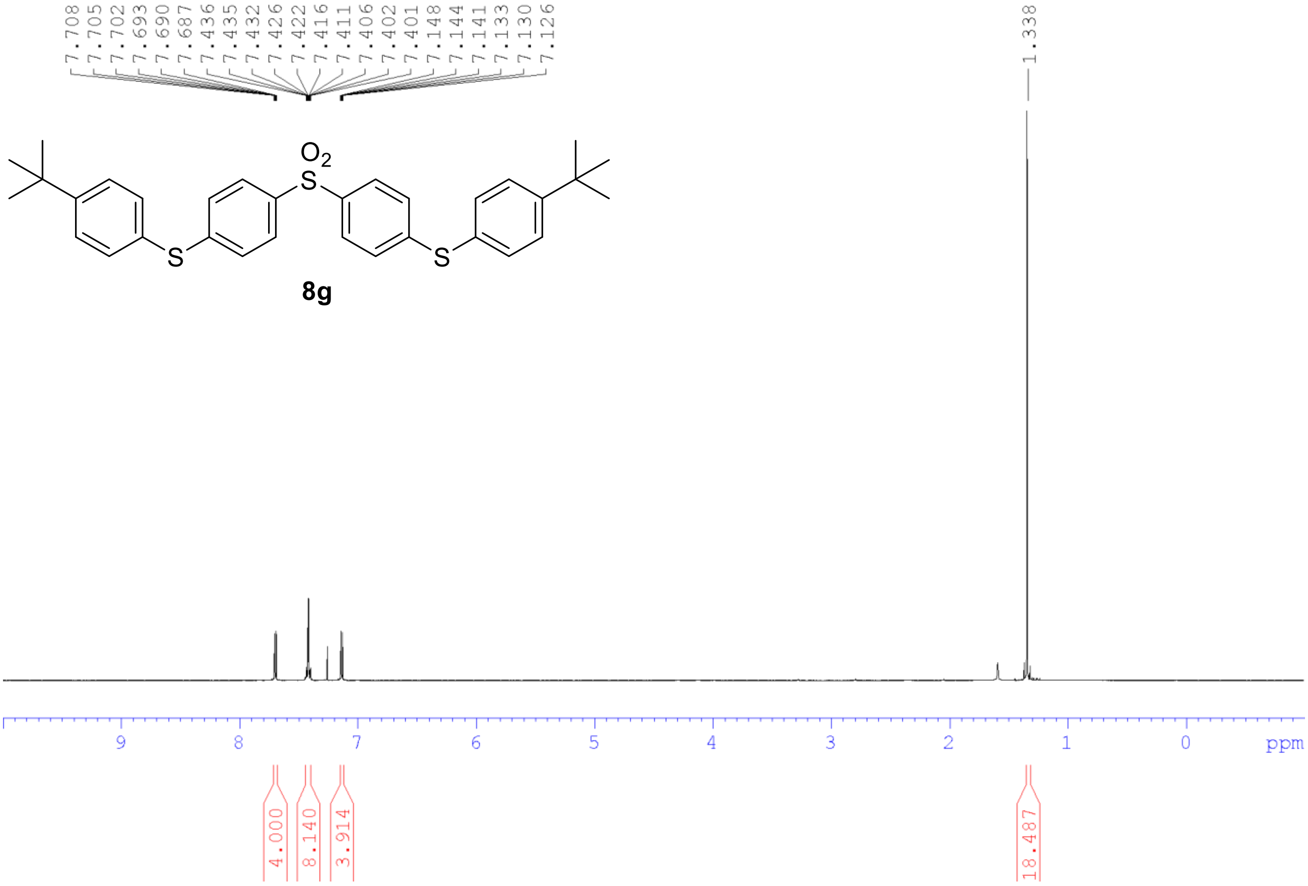

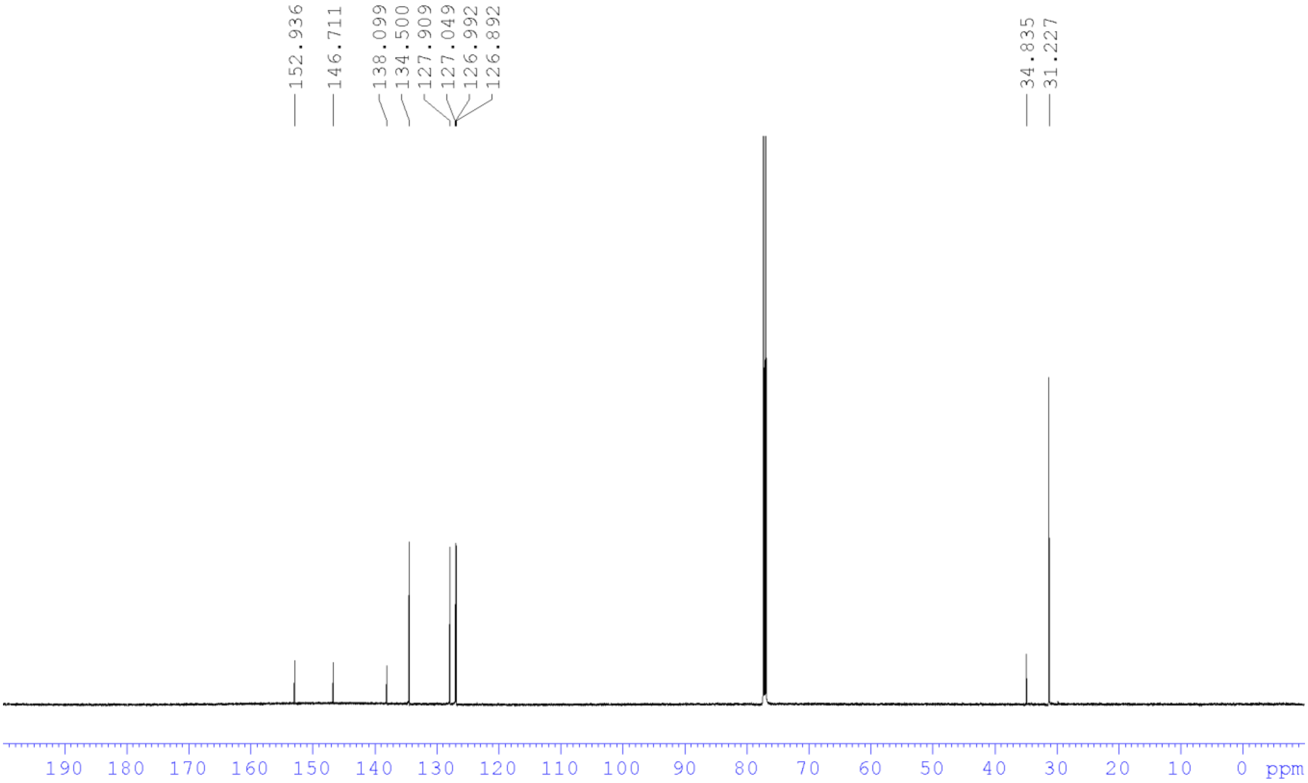

^{1}H NMR (600 MHz) and ^{13}C NMR (151 MHz) spectra of **4b** ($CDCl_3$)

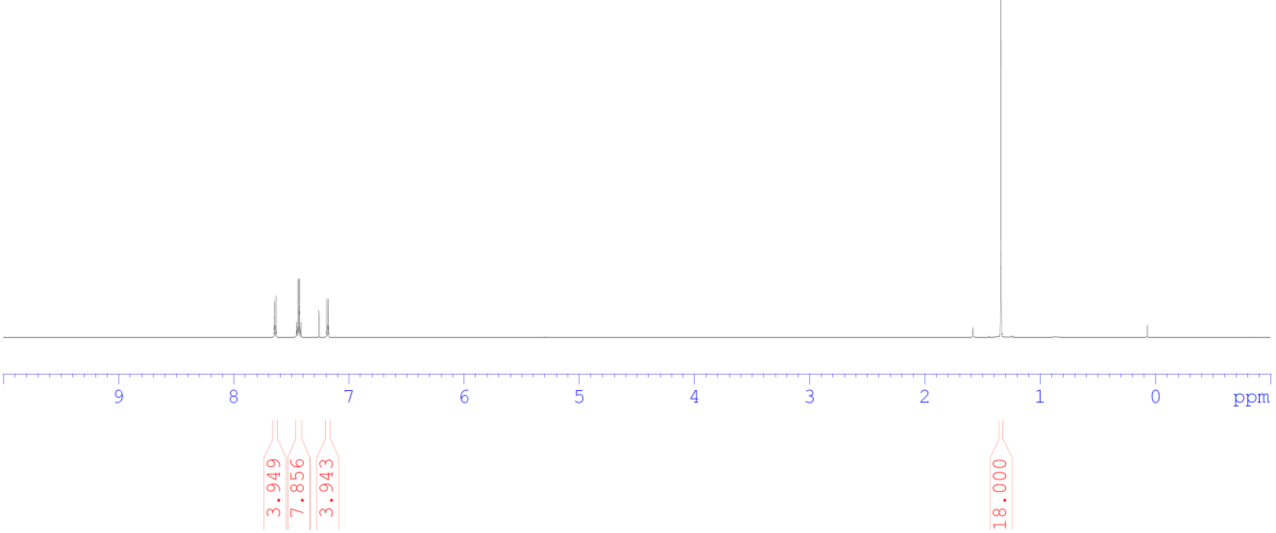

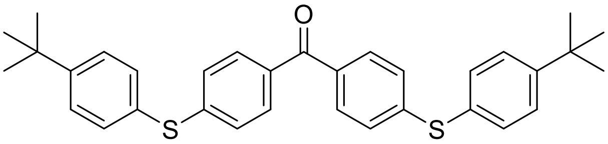

4b

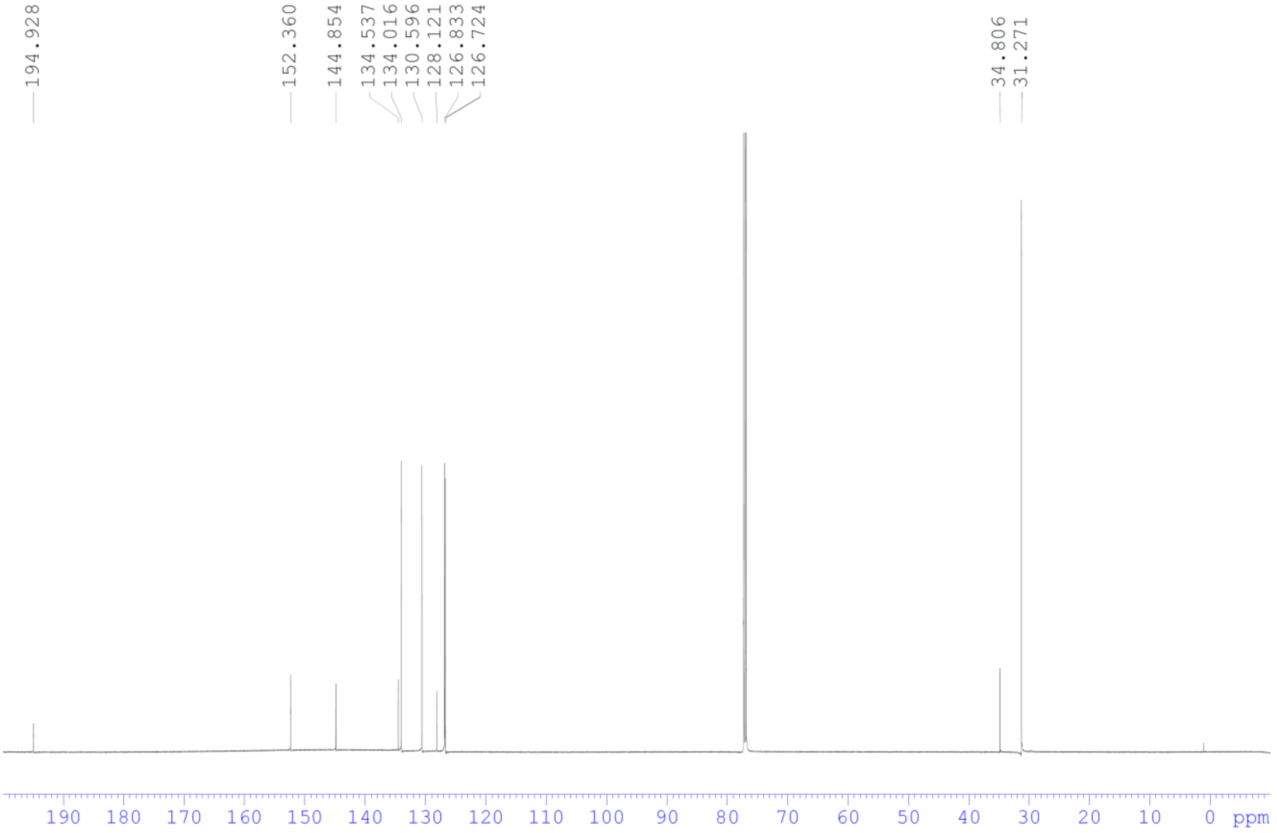

^{1}H NMR (600 MHz) and ^{13}C NMR (151 MHz) spectra of (4-((4-(Tert-butyl)phenyl)thio)phenyl)-(4-(4-hydroxyphenoxy)phenyl)methanone (**3b**) ($CDCl_3$)

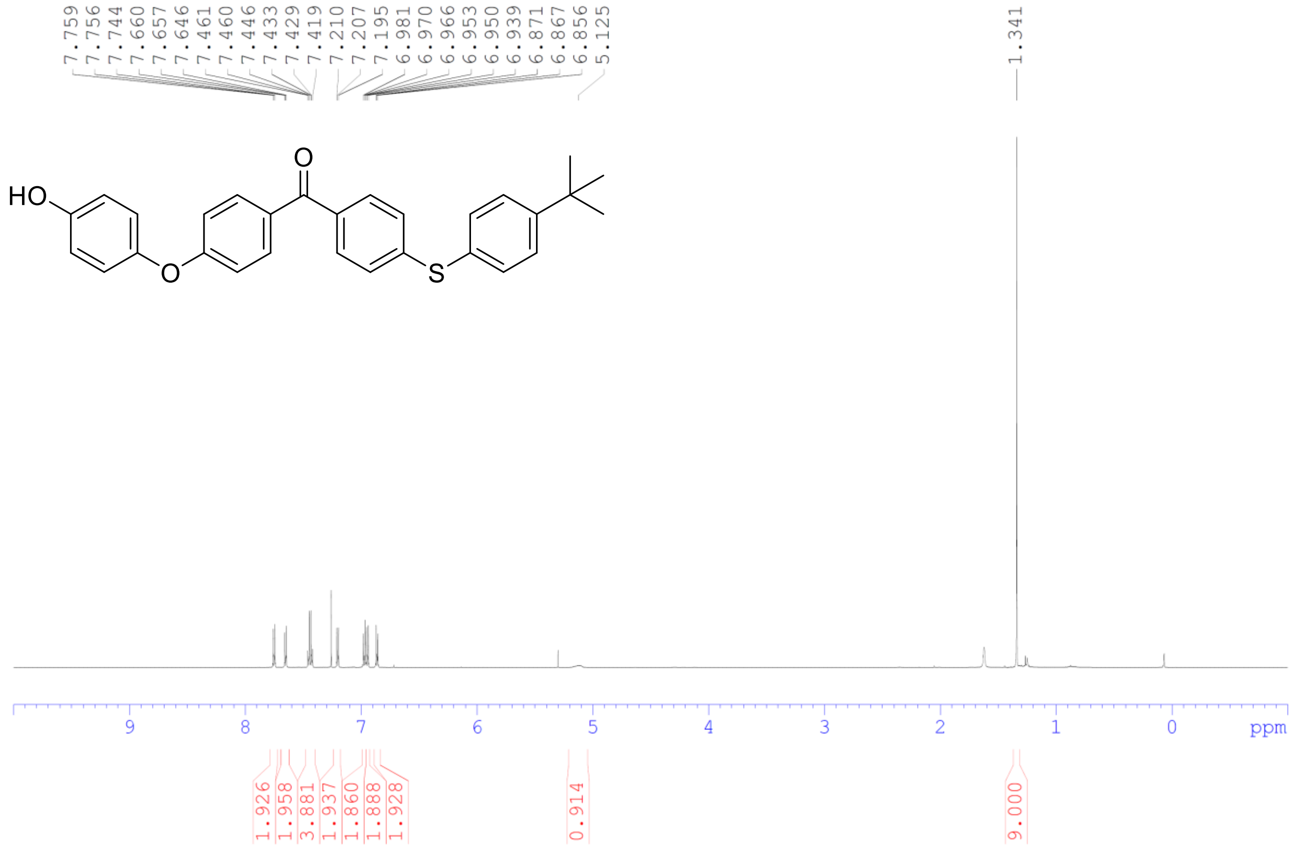

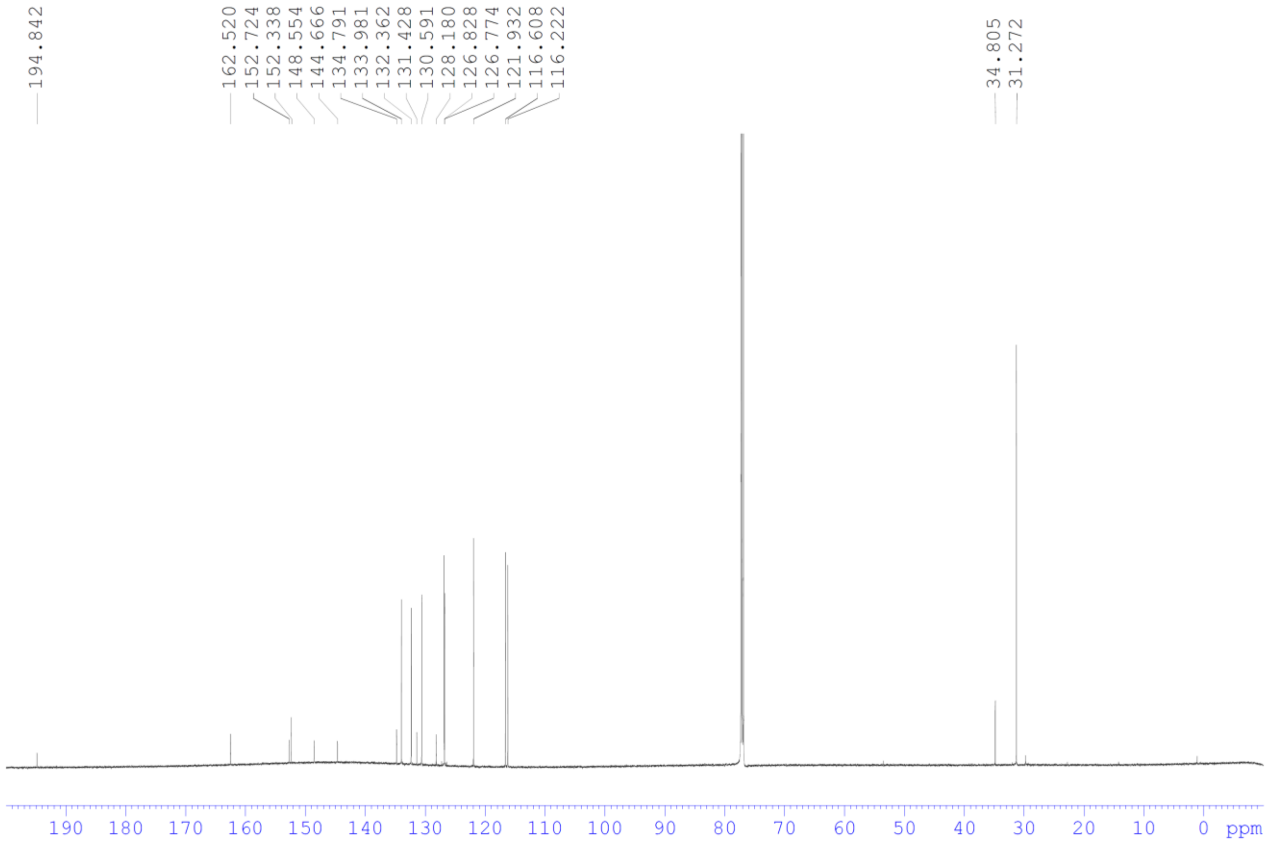

^{1}H NMR (600 MHz) and ^{13}C NMR (151 MHz) spectra of **18** ($CDCl_3$)

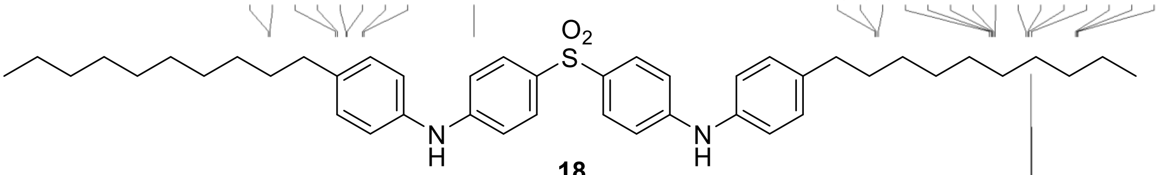

18

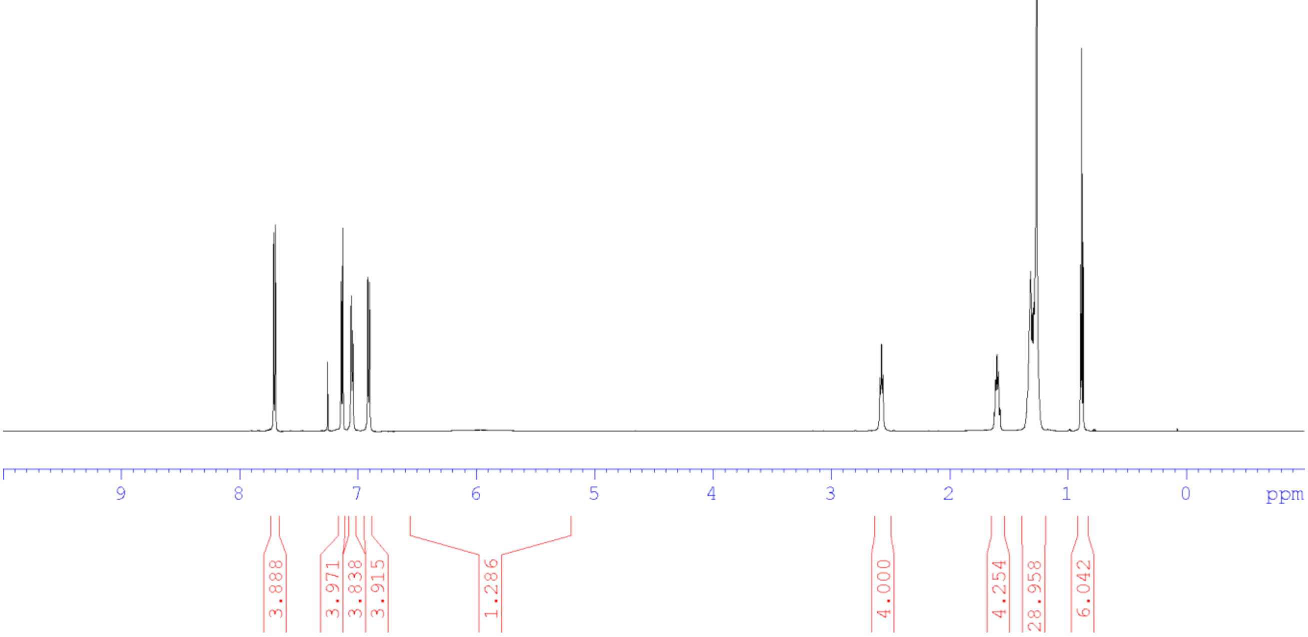

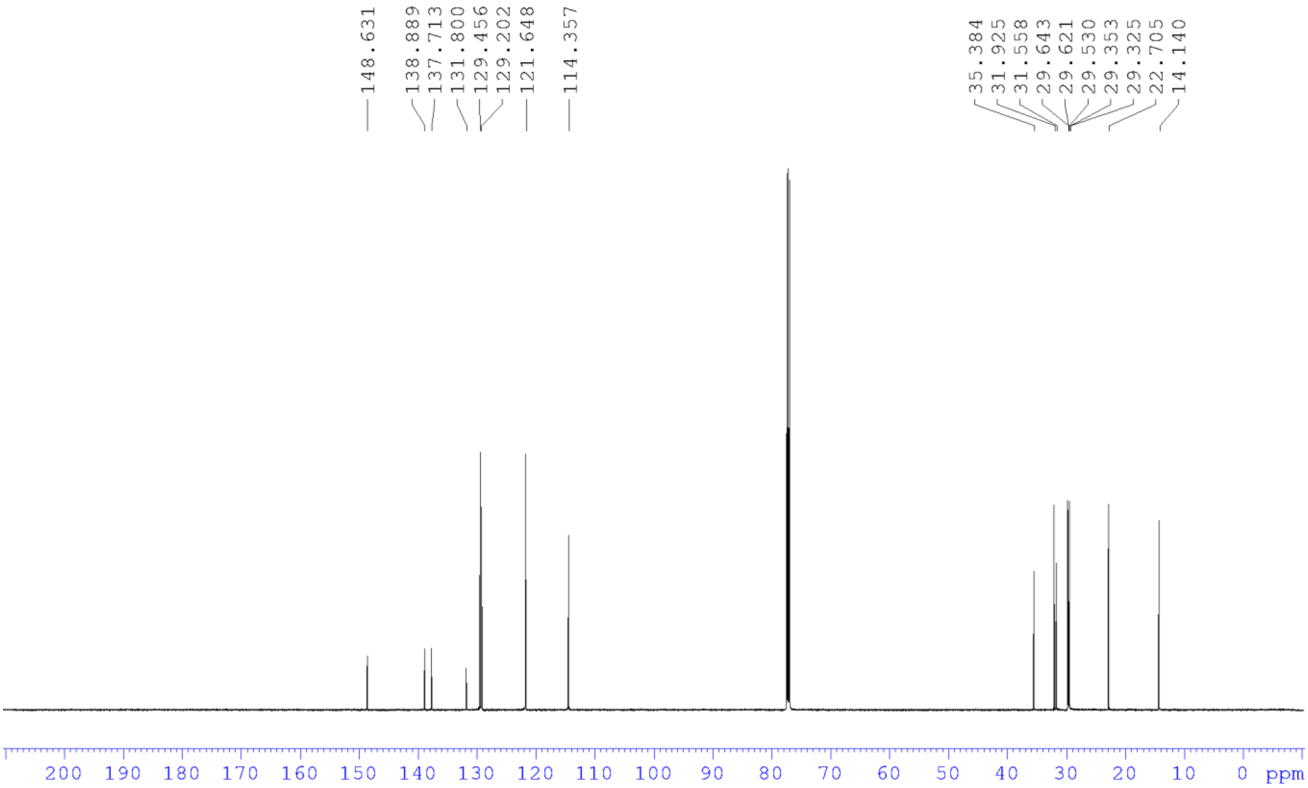

^{1}H NMR (600 MHz) and ^{13}C NMR (151 MHz) spectra of **19** ($CDCl_3$)

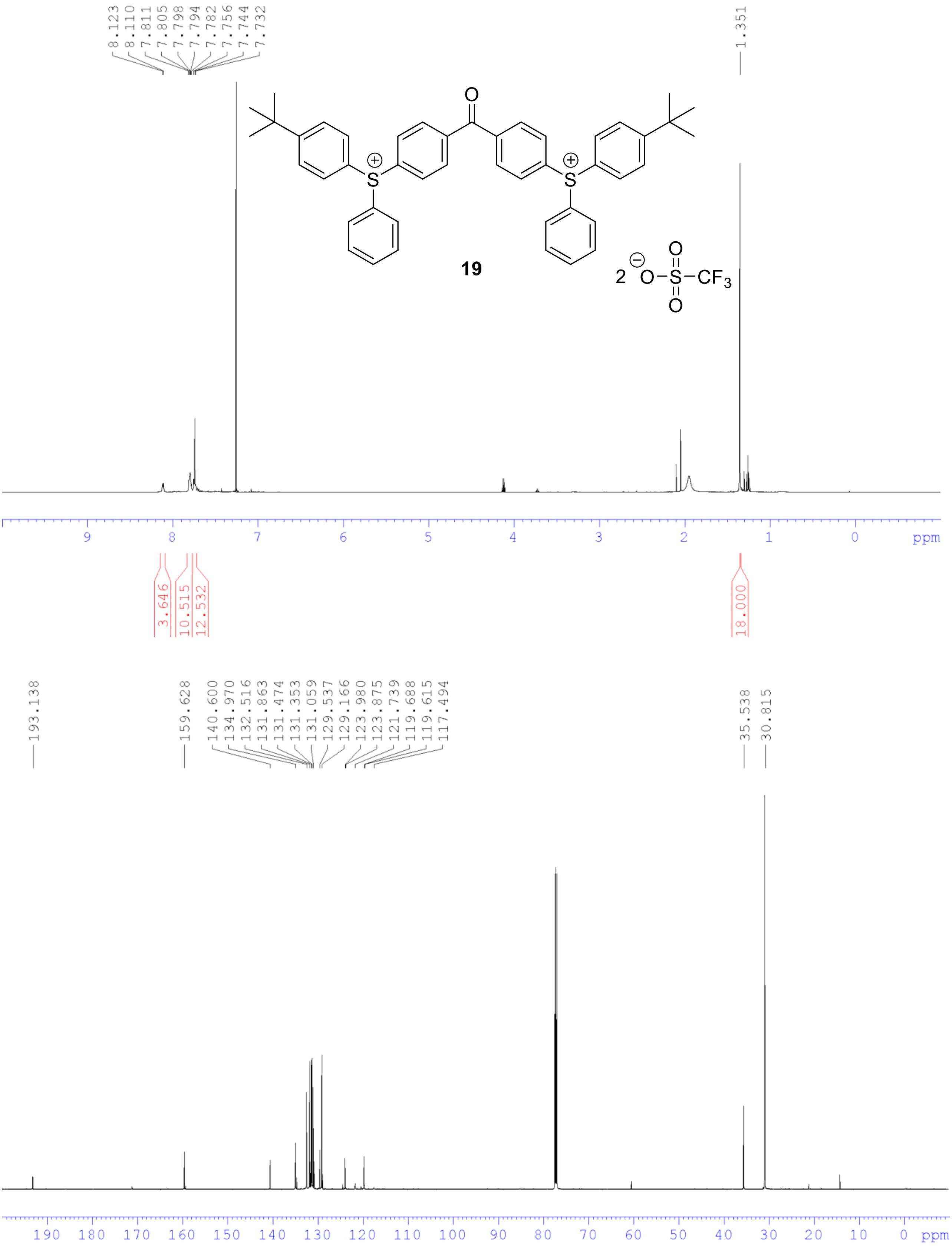

^{1}H NMR (600 MHz) spectrum of **20** ($CDCl_3$)

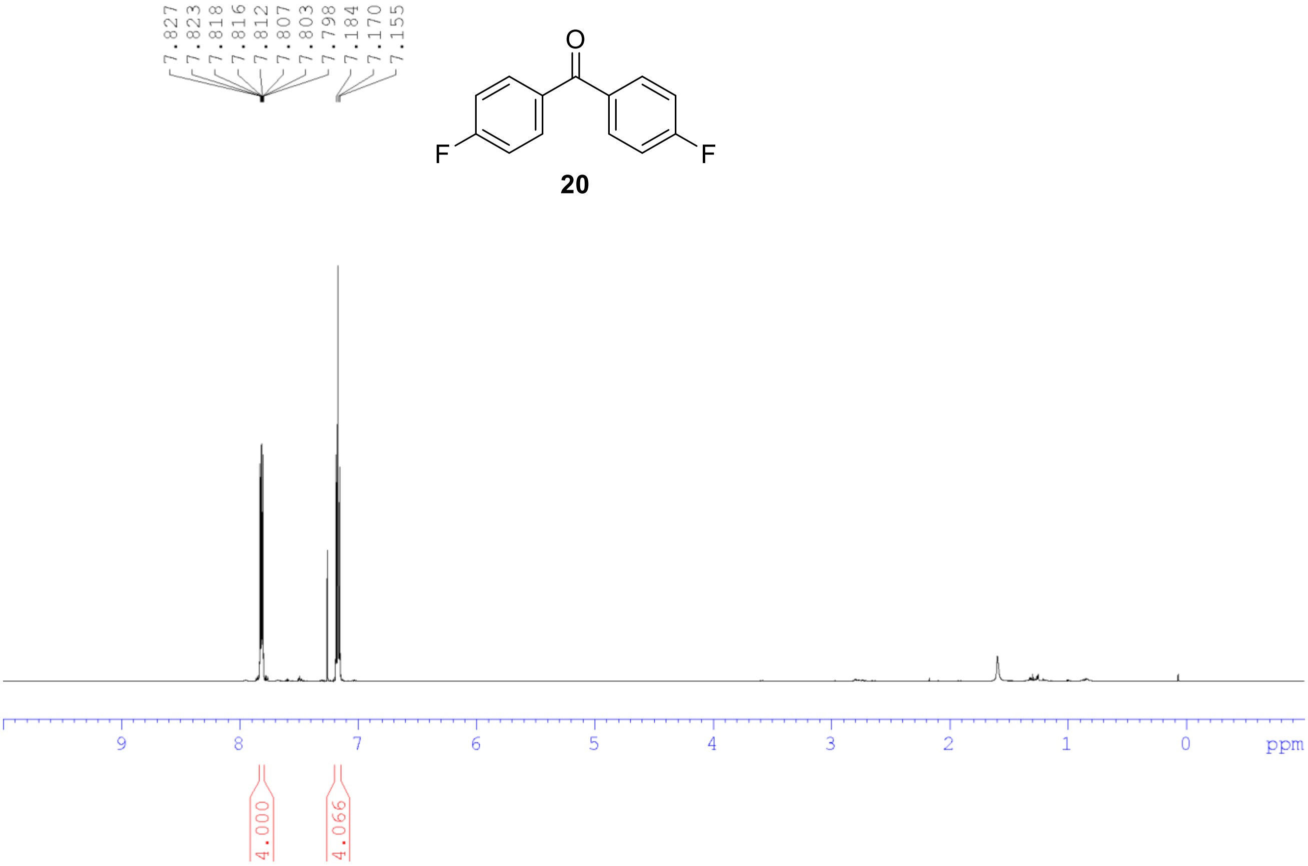

^{1}H NMR (600 MHz) and ^{13}C NMR (151 MHz) spectra of **21** ($CDCl_3$)

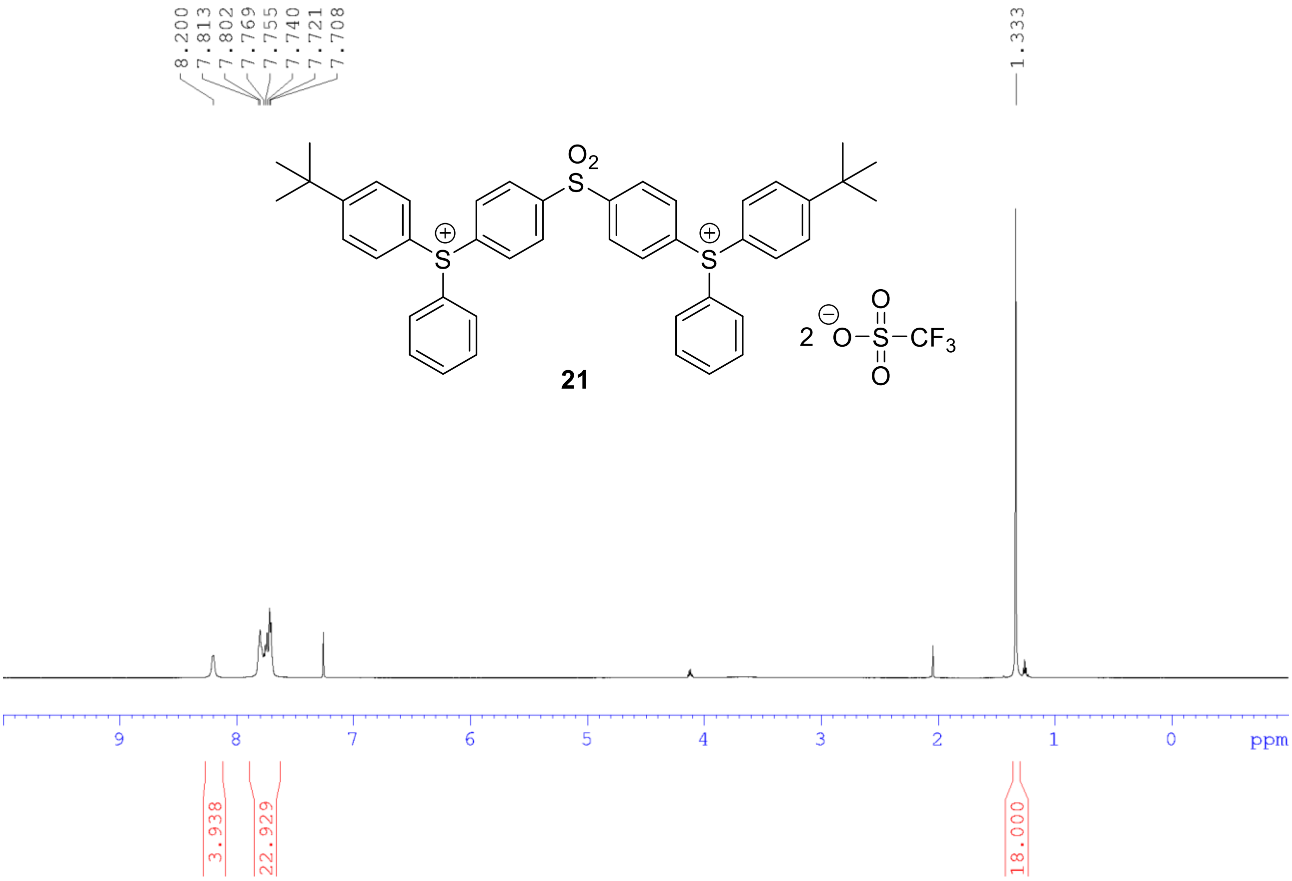

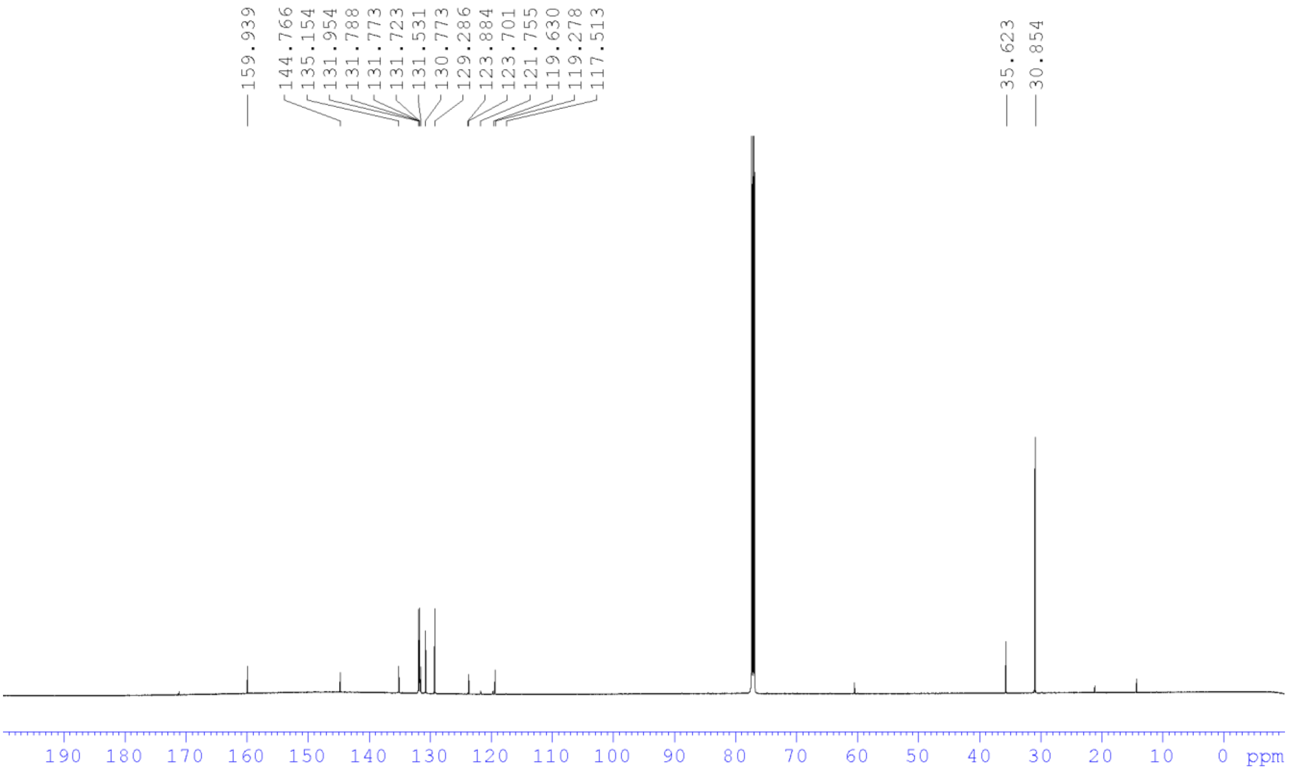

^{1}H NMR (600 MHz) spectrum of **22** ($CDCl_3$)

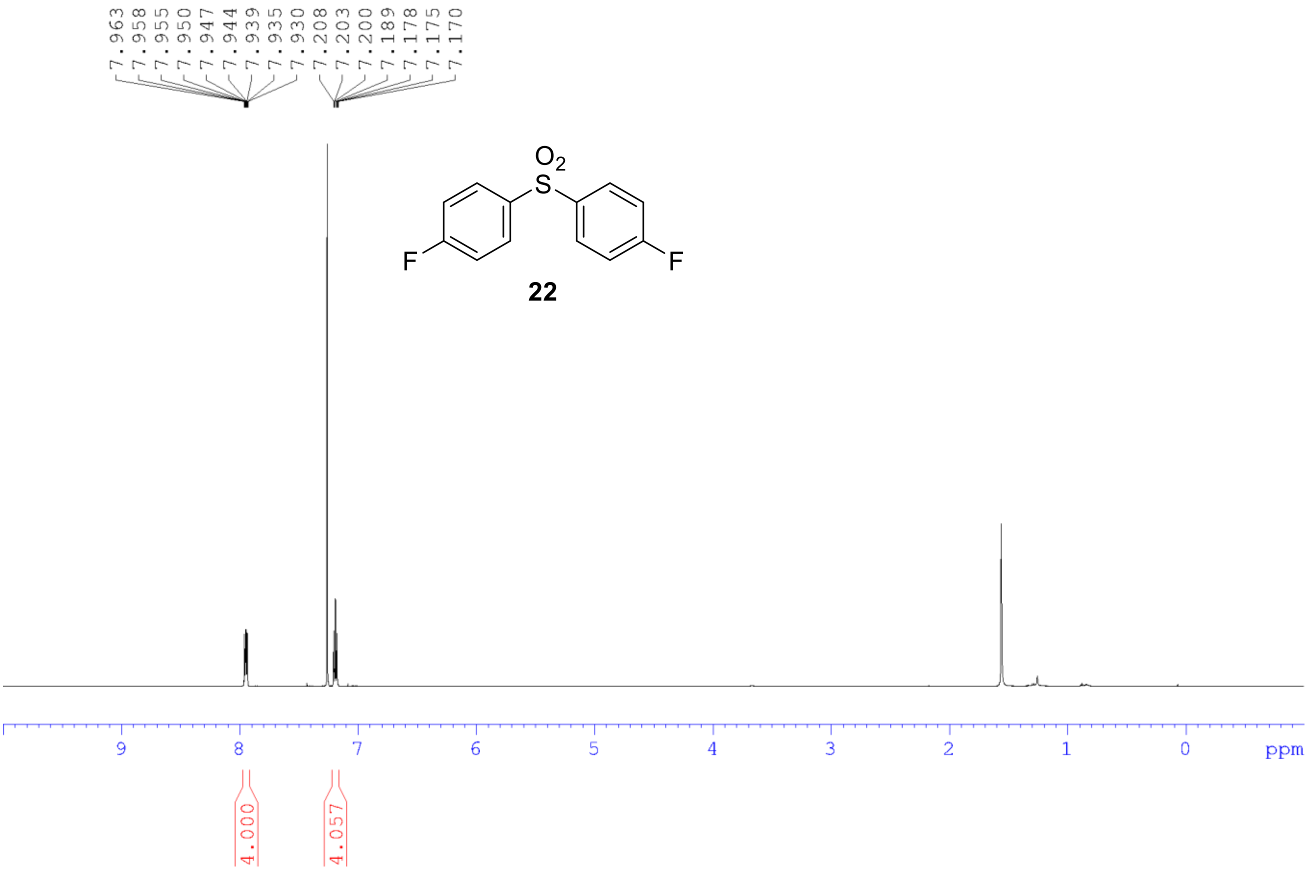

^{1}H NMR (600 MHz) and ^{13}C NMR (151 MHz) spectra of **23** ($CDCl_3$)

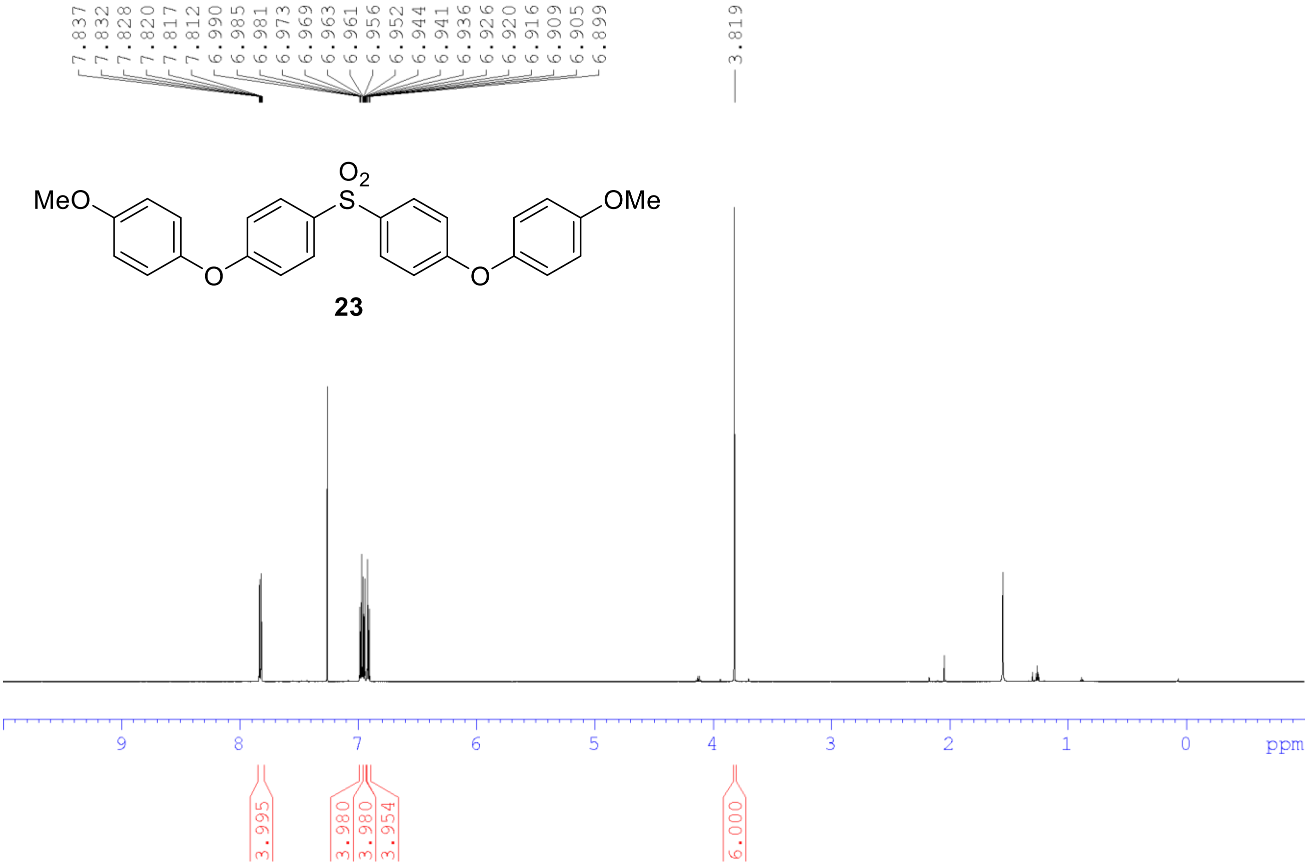

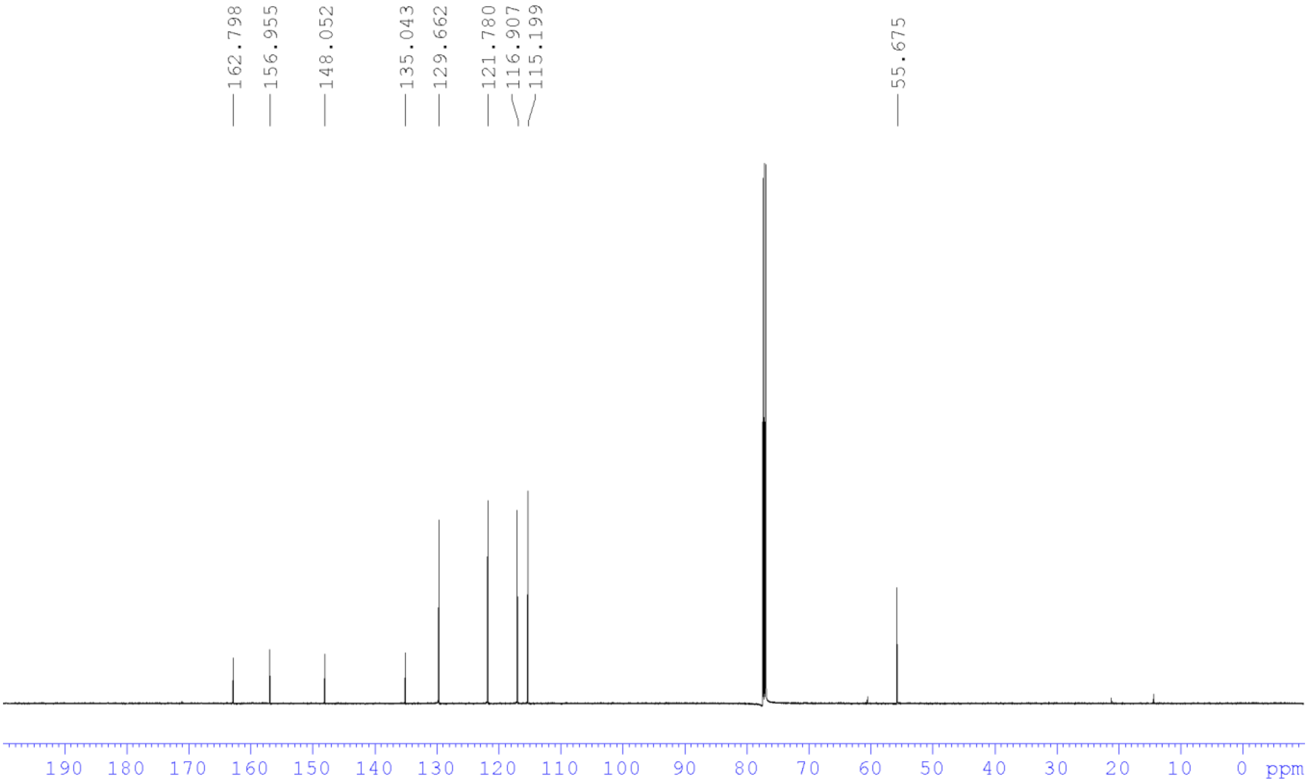

Supplement: Supplementary file 4 — Supplementary Data 1 [file 42004_2024_1120_MOESM4_ESM.pdf]
