# Supplementary material for: Catalytic thiolation-depolymerization-like decomposition of oxyphenylene-type super engineering plastics via selective carbon–oxygen main chain cleavages
Source: Commun Chem. 2024 Feb 20;7:37. doi: 10.1038/s42004-024-01120-7 (PMC10879179; doi:10.1038/s42004-024-01120-7)
Supplement: Supplementary file 5 — Supplementary Data 2 [file 42004_2024_1120_MOESM5_ESM.pdf]

Supplementary Data 2

Computed absolute electronic energies (Hartrees) and relative free Gibbs energies (kcal/mol) for all structures.

	E (B3LYP)	Correction to G (B3LYP)	G (B3LYP)
PhS anion	-629.90192770	0.059698	-629.842229
K_3PO_4	-2442.25465430	-0.021171	-2442.275825
PhS-K_3PO_4 anion complex	-3072.18720430	0.051660	-3072.135544

Optimized Molecular Geometries

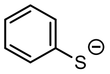

C	-2.39514	-0.25384	-0.00332
C	-0.99998	-0.25384	-0.00332
C	-0.30245	0.95391	-0.00332
C	-1.0001	2.16242	-0.00452
C	-2.39492	2.16234	-0.005
C	-3.09253	0.95413	-0.004
H	-2.9449	-1.20616	-0.00287
H	-0.45048	-1.20635	-0.00201
H	-0.4499	3.11456	-0.00458
H	-2.94505	3.11462	-0.00595
H	-4.19213	0.95432	-0.00418
S	1.47755	0.95404	-0.0023

K_3PO_4

P	-0.59294	0.10523	0.14987
O	0.29577	0.76183	-0.87164
O	-2.22723	0.46738	-0.19948
O	-0.19985	0.69054	1.70775
O	-0.36163	-1.58839	0.10216
K	-0.98	-2.50914	-2.34854
K	2.37105	0.12083	2.25732
K	-3.81589	-0.70638	1.62659

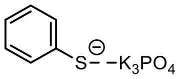

P	-2.61665	0.07754	0.00011
O	-1.41888	1.08979	-0.12542
O	-2.12852	-1.41784	0.03591
O	-3.64296	0.29076	-1.19747
O	-3.47592	0.40458	1.29979
K	0.42542	-0.78408	-0.14504
K	-3.44249	2.747	-0.06606
K	-4.72174	-1.74588	0.22922
S	3.36385	-1.99295	-0.38774
C	4.40728	-0.60107	-0.1021
C	4.77982	0.28712	-1.14417
C	4.94093	-0.31482	1.18121
C	5.61691	1.38103	-0.91802
H	4.39564	0.0954	-2.14345
C	5.77811	0.77883	1.40607
H	4.68158	-0.97789	2.00315
C	6.12772	1.6439	0.36017
H	5.87316	2.03729	-1.74998
H	6.16199	0.9592	2.41042
H	6.7787	2.49812	0.53633
H	-3.17024	1.72296	6.08322
